# The role of dopaminergic medication and specific pathway alterations in idiopathic and *PRKN/PINK1*-mediated Parkinson’s disease

**DOI:** 10.1126/sciadv.adp7063

**Published:** 2025-05-14

**Authors:** Alexander Balck, Max Borsche, Philip Campbell, Xi Luo, John Harvey, Theresa Brückmann, Charlotte Ludwig, Amy Harms, Katja Lohmann, Emmeline Brown, Huw R. Morris, Anthony H. Schapira, Thomas Hankemeier, Ronan Fleming, Silke Szymczak, Christine Klein

**Affiliations:** ^1^ Institute of Neurogenetics, University of Lübeck, Lübeck, Germany.; ^2^ Department of Neurology, University of Lübeck and University Hospital Schleswig-Holstein, Campus Lübeck, Lübeck, Germany.; ^3^ Department of Clinical and Movement Neurosciences, UCL Queen Square Institute of Neurology, London, UK.; ^4^ Digital Twin Center, School of Medicine, University of Galway, University Rd, Galway, Ireland.; ^5^ Metabolomics and Analytics Centre, Leiden Academic Centre for Drug Research, Leiden University, Leiden, Netherlands.; ^6^ Institute of Medical Biometry and Statistics, University of Lübeck, Lübeck, Germany.

## Abstract

Parkinson’s disease (PD) is the second most common neurodegenerative disease, with a rapidly increasing prevalence worldwide. Biomarkers monitoring state and progression are urgently needed, and metabolomics from easily accessible biofluids holds the potential to elucidate pathophysiological underpinnings in PD. Several studies suggested metabolomic differences between patients and controls, but findings are controversial, and independent replication is scarce. We thus applied state-of-the-art, large-scale metabolomics in patients with idiopathic and monogenic PD and controls from two independent samples, analyzed by a strict meta-analysis approach. Thereby, we (i) debunked that l-Dopa medication and not disease status causes the most substantial metabolomic differences and (ii) identified polyamine metabolism alterations, partly, but not entirely associated with l-Dopa treatment. Furthermore, we found explorative but robust evidence for alterations in endocannabinoid metabolites; detected lipid metabolism alterations, highlighting potential crosslinks with alpha-synuclein pathology; and provided evidence for a metabolomic signature for the role of oxidative damage in patients with *PRKN*- and *PINK1*-linked PD.

## Teaser

L-Dopa medication substantially affects the Parkinson’s metabolome, while specific pathway alterations might be disease dependent.

## INTRODUCTION

Parkinson’s disease (PD) is the second most common neurodegenerative disease with increasing prevalence worldwide (*1*). However, there are no blood-based biomarkers available in clinical practice (*2*), and metabolomics from easily accessible biofluids as blood hold the potential to assess disease state and progression. Studies investigating metabolites in the blood of patients with Parkinson’s disease (PD) have suggested alterations in a plethora of metabolites and biochemical pathways (*3*). However, only a small fraction of the suggested metabolites have been replicated in independent studies (*4*). Possible explanations lie in major technological and procedural pitfalls that can substantially influence the results and, therefore, must be accounted for in metabolomic studies. First, almost every patient with PD is treated with dopaminergic medication, which hampers the discrimination between disease- and drug-related effects; this is especially true of dopamine metabolism. Therefore, adjusting for treatment in statistical analyses may eliminate not only the effect of Levodopa (l-Dopa) but also the alterations caused by the disease itself when comparing only l-Dopa–treated PD patients to healthy controls (HCs). Second, as metabolite concentrations are very susceptible to handling, processing, and type of analysis, even slight differences in the procedures across laboratories can considerably affect the results (*4*). Third, sex, age, and lifestyle also substantially affect the metabolome (*5*).

Adding another level of complexity, PD is a heterogeneous disorder, including several forms of monogenic PD, such as *PRKN*- or *PINK1*-linked PD (*6*, *7*). Most likely, differences in the underlying molecular course will involve different biochemical pathways, resulting in different levels of specific metabolites for each genetic subset of patients. For example, *PRKN-* and *PINK1*-linked PD involves mitochondrial dysfunction (*8*), making alterations in pathways of oxidative phosphorylation more likely.

An important role has been attributed to lipids in the pathogenesis of PD, as genetic alterations in several genes involved in lipid metabolism have been identified to cause (e.g., *PINK1*) PD or are involved in disease pathogenesis (e.g., *VPS13C*) (*9*). Strong evidence suggests that membrane lipids are highly important for alpha-synuclein (α-syn) metabolism, contributing to α-syn fibrilization and accumulation in laboratory models (*10*). Strikingly, α-syn–lipid interactions are likely an essential component in Lewy body formation and, possibly, for spreading pathology (*9*). Among other lipids, levels of phosphatidylcholines, lysophosphatidylcholines, and phosphatidylethanolamines (PEs) have been found to discriminate patients with idiopathic PD (IPD) (*11*, *12*) and carriers of pathogenic *LRRK2* (*13*) variants from HC. However, these lipids have yet to be investigated in detail in biallelic *PRKN*- or *PINK1*-linked PD. Furthermore, investigations of the lipid metabolism in patients with drug-naïve IPD are scarce. One study on patients with untreated IPD had suggested different free fatty acids, indolelactic acid, and phenylacetylglutamine to be able to distinguish patients from HC (*14*).

Our study addressed two aims: (i) To take the influence of dopaminergic treatment on blood-based metabolomics into account, hypothesizing that this medication has a substantial impact on metabolomics in patients with IPD and monogenic PD and (ii) to identify dopaminergic medication–independent metabolites with different intensities in idiopathic and *PRKN/PINK1*-linked PD compared to HC by large-scale targeted metabolomics, hypothesizing that distinct alterations in metabolites might be characteristic for (“mitochondrial”) PD.

## RESULTS

### Study population

The study sample at both study sites consists of a total of 223 participants. The 140 patients with IPD [55 females (39%)] had a median age at examination of 62 years [interquartile range (IQR) of 54 to 70 years; range, 28 to 84 years], a median age at onset of 54 years (IQR of 46 to 63 years; range, 25 to 81 years], and a median disease duration of 6.3 years (IQR of 4.1 to 10.9 years; range, 0.9 to 22.9 years). The 19 *PRKN/PINK1*-linked PD patients [9 females (47%)] had a median age at examination of 52 years (IQR of 47 to 59 years; range, 24 to 72 years), a median age of onset of 31 years (IQR of 23 to 37 years; range, 13 to 66 years), and a median disease duration of 16 years (IQR of 9.6 to 30.2; range, 4.0 to 48.3 years). The HC group comprised 64 individuals with a median age at examination of 62 years (IQR of 33 to 67 years; range, 25 to 80 years). Clinical scores, l-Dopa equivalent daily dose (LEDD), and daily l-Dopa dose are shown in Table 1.

**Table 1.** Overview of the study groups: Patients with IPD were separated into non–l-Dopa–treated IPD patients (l-Dopa^negative^) and l-Dopa–treated IPD patients (l-Dopa^positive^). l-Dopa^negative^ patients included individuals that were treated with dopamine agonists or untreated. All numerical variables are displayed with mean (1. quartile – 3. quartile; Min. to Max.). MitoPD = *PRKN/PINK1*-linked PD patients; HC = healthy controls; F = female; M = male; PD = Parkinson’s disease; AAO = Age at onset; AAE = Age at examination; LEDD = Levodopa equivalent daily dosage; MDS UPDRS III = Movement Disorder Society Unified Parkinson’s Disease rating scale part III; H/Y = Hoehn and Yahr stage.

	All IPD	l-Dopa^negative^	l-Dopa^positive^	MitoPD	HC
***n***	140	30	110	19	64
**AOO (years)**	54 (46–63; 25–81)	49 (42–58; 25–69)	55 (48–65; 31–81)	31 (23–37; 13–66)	NA
**AAE (years)**	62 (54–70; 28–84)	54 (49–61; 28–78)	65 (58–71; 37–84)	52 (47–59; 24–72)	62 (33–67; 25–80)
**Disease duration (years)**	6.3 (4.1–10.9; 0.9–22.9)	4.3 (2.9–6.5; 0.9–22.9)	6.5 (4.6–11.4; 0.9–22.8)	16.3 (9.6–30.2; 4.0–48.3)	NA
**LEDD (mg)**	499.5 (300–804; 0–1782)	147.5 (85–209; 0–500)	595 (409–954; 127–1782)	350 (255–694; 0–1288)	0
**Daily l-Dopa dose (mg)**	300 (150–506; 0–1225)	0	375 (300–600; 37.5–1225)	225 (112.5–312.5; 0–750)	0
**MDS UPDRS III**	25 (18–37; 4–70)	25 (18–35; 6–69)	25.5 (18–38; 4–70)	25 (16–34; 6–64)	3 (2–6; 0–14)
**H/Y**	2 (1–2; 1–4)	2 2 (1–2; 1–3)	2 (1–2; 1–4)	2 (2–3; 1–5)	0
**Sex**	F: 55 (39%); M: 85 (61%)	F: 15 (50%); M: 15 (50%)	F: 40 (36%); M: 70 (64%)	F: 9 (47%); M: 10 (53%)	F: 34 (53%); M: 30 (47%)

### Metabolomics

After preprocessing and quality control, 304 metabolites were available for analysis of which 32, 51, 9, 138, and 74 were acylcarnitines, amines, organic acids, lipids, and signaling lipids, respectively (see table S1 for an overview of all measured metabolites and their annotation).

In the exploration of recruitment site as batch effect, 100 metabolites had an adjusted *P* value < 0.05 for recruitment site. However, only 17 significant metabolites had an absolute effect size >1 (table S2). As described in Materials and Methods, all further analyses were stratified for recruitment site, and presented results correspond to the effects and *P* values of a fixed effect meta-analysis combining the effects of the two strata.

When combining patients with IPD and HCs, 30 of the 304 metabolites were significantly associated with age at examination (based on adjusted *P* values) (table S3). Regarding amino acids, we found tyrosine, cysteine, its precursors cystathionine, and *O*-acetylserine, as well as l-Homocitrulline, to be elevated. Two amino acids, tryptophan and threonine, were significantly reduced with increasing age. Five acylcarnitines were significantly elevated with age (isobutyrylcarnitine, tiglylcarnitine, trimethylamine *N*-oxide, tetradecanoylcarnitine, and nonanoylcarnitine), and three sphingomyelins with different lipid-lengths were significantly reduced with age [sphingomyelin(d18:1/22:0), (d18:1/24:0), and (d18:1/24:1)]. Notably, we also found citrate and isocitrate, both part of the citric acid cycle, to be significantly elevated with increasing age.

### Analyses

Because of the site effects described in Materials and Methods, the final analyses reported in the text refer to a meta-analysis. The figures show the individual-level data for the specific metabolites; forest plots summarizing the site specific and combined effect estimates are shown in fig. S1. For comparison purposes, we also conducted a discovery/replication stage analysis using the University College London (UCL) cohort as the discovery cohort.

### l-Dopa treatment but not dopamine agonist treatment increases l-Dopa–dependent metabolites in IPD

**Fig. 1.**
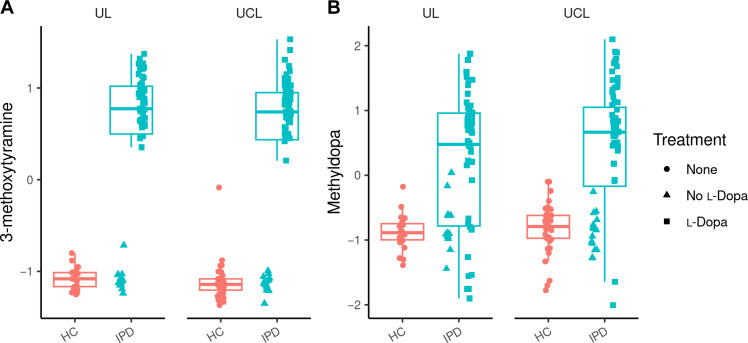
Levels of 3-methoxytyramine and methyldopa are increased in patients with IPD treated with l-Dopa (l-Dopa^positive^) but not elevated in untreated patients and patients with agonist treatment only (l-Dopa^negative^). (**A** and **B**) HC, healthy controls; UL, University of Lübeck; UCL, University College London. Data were analyzed as described in the statistics section. No significance levels are depicted in the figure, as data uncorrected for site effects are shown, while statistical calculations were performed by meta-analysis as described in the methods part.

First, we explored differences in metabolites comparing all patients with IPD (*n* = 140) and HC. Here, we identified five metabolites, i.e., 3-methoxytyramine, methyldopa, putrescine, (+/−)-16-hydroxydocosahexaenoic acid (16-HDoHE), and ornithine, as the only significant metabolites after correction for multiple testing (Table 2 and table S4). We observed a heterogeneous distribution within the IPD group, particularly for 3-methoxytyramine and methyldopa, as these metabolites were predominantly increased in l-Dopa–treated individuals (in the following referred to as l-Dopa^positive^), but not similarly in untreated patients with IPD and individuals only treated with dopamine agonists (Fig. 1). Concluding that l-Dopa treatment is responsible for these notable differences between patients with IPD and HC, we continued analyzing the l-Dopa^positive^ and the l-Dopa^negative^ group separately to elucidate differences in the metabolome not caused by dopaminergic treatment. The demographical and clinical data of the two IPD subgroups are likewise displayed in Table 1.

**Table 2.** Metabolites differently expressed in patients with IPD (*n* = 140) compared to HCs (*n* = 64). Shown are the results of the fixed effect meta-analysis with a nominal *P* value below *P* < 0.05: regression coefficient beta (CI: 95% confidence interval), nominal and adjusted *P* values. Metabolites with an adjusted *P* < 0.05 are displayed in italics. The complete table with all metabolites can be found in table S4.

Name	*z* value	Nominal *P*	Adjusted *P*	β (CI)
*3-Methoxytyrosine*	*13.376*	*8.38 × 10^−41^*	*2.55 × 10^−38^*	*1.514 (1.292; 1.736)*
*Methyldopa*	*8.624*	*6.45 × 10^−18^*	*9.8 × 10^−16^*	*1.179 (0.911; 1.447)*
*Putrescine*	*4.321*	*0.0000155*	*0.00157*	*0.5 (0.273; 0.726)*
*(+/−) 16-HDoHE*	*3.906*	*0.000094*	*0.00714*	*0.629 (0.313; 0.945)*
*Ornithine*	*3.6*	*0.000318*	*0.0194*	*0.516 (0.235; 0.797)*
*-Threonine*	**2*.*897**	*0.00377*	*0.191*	*0.464 (0.15; 0.779)*
Triglyceride (54:7)	−2.701	0.00692	0.301	−0.466 (−0.805; −0.128)
1-Arachidonoyl Glycerol / 2-Arachidonoyl Glycerol	−2.589	0.00963	0.366	−0.413 (−0.726; −0.1)
Triglyceride (54:1)	−2.476	0.0133	0.378	−0.427 (−0.764; −0.089)
N6,N6,N6-Trimethyl-l-lysine	2.472	0.0134	0.378	0.378 (0.078; 0.677)
Tiglylcarnitine	−2.465	0.0137	0.378	−0,406 (−0,728; −0.083)
Homocysteine	2.372	0.0177	0.399	0.353 (0.061; 0.644)
Triglyceride (50:4)	−2.368	0.0179	0.399	−0.4 (−0.73; −0.069)
2-ketoglutaric acid	−2.317	0,0205	0.399	−0.362 (−0.669; −0.056)
Phosphatidylcholine (38:4)	−2.287	0.0222	0.399	−0.405 (−0.751; −0.058)
Phosphatidylcholine (O-36:5)	−2.252	0.0243	0.399	−0.395 (−0,739; −0.051)
Methylmalonylcarnitine	−2.227	0.026	0.399	−0.341 (−0.641; −0.041)
Gamma-Glutamylglutamine	2.211	0.027	0.399	0.35 (0.04; 0.661)
Triglyceride (54:5)	−2.208	0.0273	0.399	−0.386 (−0.729; −0.043)
Phosphatidylethanolamine (O-38:5)	−2.173	0.0298	0.399	−0.396 (−0.753; −0.039)
Phosphatidylcholine (38:7)	−2.171	0.0299	0.399	−0.378 (−0.719; −0.037)
Cystathionine	2.131	0.0331	0.399	0.286 (0.023; 0.549)
Triglyceride (52:4)	−2.124	0.0337	0.399	−0.376 (−0.724; −0.029)
Phosphatidylcholine (36:4)	−2.112	0.0347	0.399	−0.369 (−0.712; −0.027)
Triglyceride (48:3)	−2.088	0.0368	0.399	−0.334 (−0.648; −0.02)
Glycoursodeoxycholic acid	−2.084	0.0371	0,399	−0.281 (−0.545; −0.017)
Phosphatidylethanolamine (O-36:5)	−2.063	0.0391	0.399	−0.372 (−0.726; −0.019)
Triglyceride (51:4)	−2.063	0,0391	0.399	−0.346 (−0.674; −0.017)
Triglyceride (56:6)	−2.061	0.0393	0.399	−0.362 (−0.706; −0.018)
Glycochenodeoxycholic acid	−2.06	0.0394	0.399	−0.308 (−0.6; −0.015)
Ceramide (d18:0/22:0)	−2.047	0.0407	0.399	−0.352 (−0.69; −0.015)

Considering further factors potentially influencing these five metabolites in all patients with IPD, we investigated a possible association between 3-methoxytyramine, methyldopa, putrescine, 16-HDoHE, and ornithine and disease duration. Here, we only found a weak association between 16-HDoHE levels and disease duration {regression coefficient = 0.057 [confidence interval] (CI): 0.023 to 0.091, adjusted *P* = 0.028}, which was not present in the other four metabolites.

**Fig. 2.**
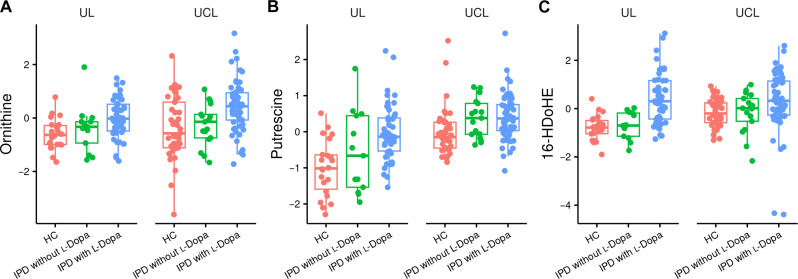
(+/−)-16-HDoHE, ornithine, and putrescine are increased in patients with IPD treated with l-Dopa (l-Dopa^positive^) but not elevated in untreated patients and patients with agonist treatment only (l-Dopa^negative^). (**A** to **C**) Data were analyzed as described in the “Statistics” section. No significance levels are depicted in the figure, as data uncorrected for site effects are shown, while statistical calculations were performed by meta-analysis as described in Materials and Methods.

Investigating the l-Dopa^positive^ group separately, we identified the same five metabolites already mentioned above, i.e., methoxytyramine, methyldopa, 16-HDoHE, ornithine, and putrescine, to be significantly elevated in patients compared to HC after adjusting for multiple testing (Fig. 2, Table 3, and table S5). As expected, the l-Dopa downstream metabolites (*15*) methoxytyramine and methyldopa (adjusted *P* < 0.0001) were highly elevated. Despite also accounting for dopaminergic treatment other than l-Dopa, the LEDD was associated with the levels of both metabolites in the l-Dopa^positive^ group [methoxythyramine: regression coefficient = 0.55 (CI: 0.42 to 0.66, adjusted *P* < 0.0001); methyldopa: regression coefficient = 0.39 (CI: 0.24 to 0.52, adjusted *P* < 0.0001)]. As expected, the daily l-Dopa dose in l-Dopa^positive^ individuals was also strongly associated with methoxytyramine and methyldopa levels [methoxythyramine: regression coefficient = 4 × 10^−04^ (CI: 0.00021 to 0.00060, adjusted *P* = 0.0002); methyldopa: regression coefficient = 0.00127 (CI: 0.00060 to 0.00195, adjusted *P* = 0.0004)].

**Table 3.** Metabolites differently expressed in patients with IPD treated with l-Dopa (Dopa^positive^; *n* = 110) compared to HCs (*n* = 64). Shown are the results of the fixed effect meta-analysis with a nominal *P* value below *P* < 0.05: regression coefficient β (CI: 95% CI), nominal and adjusted *P* values. Metabolites with an adjusted *P* < 0.05 are displayed in italics. The complete table with all metabolites can be found in table S5.

Name	*z* value	Nominal *p*	Adjusted *p*	Beta (CI)
*3-Methoxytyrosine*	*1.95*	*0*	*0*	*1.95 (1.873; 2.026)*
*Methyldopa*	*1.571*	*6.36 × 10^−40^*	*9.67 × 10^−38^*	*1.571 (1.339; 1.804)*
*(+/−) 16-HDoHE*	*0.809*	*2.24 × 10^−06^*	*0.000227*	*0.809 (0.474; 1.144)*
*Putrescine*	*0.547*	*6.39 × 10^−06^*	*0.000485*	*0.547 (0.309; 0.784)*
*Ornithine*	*0.582*	*6.36 × 10^−05^*	*0.00387*	*0.582 (0.297; 0,868)*
N6,N6,N6-trimethyl-l-lysine	0.493	0.00265	0.135	0.493 (0.171; 0.814)
l-Threonine	0.486	0.00366	0.159	0.486 (0.158; 0.813)
Homocysteine	0.403	0.00949	0.309	0.403 (0.099; 0.708)
l-Tyrosine	0.416	0.0102	0.309	0.416 (0.099; 0.734)
2-ketoglutaric acid	−0.424	0.00907	0.309	−0.424 (−0.742; −0.105)
Triglyceride (54:7)	−0.446	0.0144	0.398	−0.446 (−0.803; −0.089)
Triglyceride (54:1)	−0.39	0.0161	0.407	−0.39 (−0.708; −0.073)
Methylmalonylcarnitine	−0.367	0.0176	0.41	−0.367 (−0.67; −0.064)
Cystathionine	0.324	0.0196	0.425	0.324 (0.052; 0.597)
Gamma-glutamylglutamine	0.377	0.0232	0.47	0.377 (0.052; 0.703)
Glycocholic acid	−0.321	0.0301	0.571	−0.321 (−0.611; −0.031)
Glycochenodeoxycholic acid	−0.345	0.0319	0.571	-0.345 (−0.66; -0.03)
Deoxycarnitine	−0.266	0.0346	0.584	-0.266 (−0.513; -0.019)
Saccharopine	0.354	0.0403	0.625	0.354 (0.016; 0.692)
2-hydroxybutyric acid	−0.361	0.0425	0.625	-0.361 (−0.711; -0.012)
Glycoursodeoxycholic acid	−0.295	0.0473	0.625	-0.295 (−0.587; -0.004)
Cer(d18:0/22:0)	−0.345	0.0444	0.625	-0.345 (−0.68; -0.009)
Phosphatidylcholine (38:4)	−0.375	0.0456	0.625	-0.375 (−0.742; -0.007)

We confirmed these findings by applying a discovery/replication design analysis using the UCL cohort as the discovery cohort because it contains more patients with IPD and controls. Fifteen metabolites were selected for the replication stage (table S6), including 3-methoxytyramine, methyldopa, ornithine, and putrescine, but not (+/−)-16-HDoHE bceause the nominal *P* value was 0.125. Testing these metabolites in the UL cohort, 3-methoxytyramine, methyldopa, ornithine, and putrescine could be replicated (tables S7 and S8).

### Differences between l-Dopa^negative^ IPD patients and HCs

When comparing l-Dopa^negative^ IPD patients to HC, there were no significant differences in metabolite concentration if only adjusted *P* values were considered. Notably, the most strikingly elevated metabolites in l-Dopa^positive^ individuals, i.e., methoxytyramine and methyldopa, were not at all increased in l-Dopa^negative^ IPD patients compared to controls (Fig. 1). Regarding 16-HDoHE, ornithine, and putrescine, there was no difference in l-Dopa^negative^ IPD patients compared to HC after adjusting for multiple testing. However, putrescine displayed a nominal *P* value of 0.026 [adjusted *P* = 0.56, regression coefficient = 0.386 (CI: 0.046 to 0.725)] (Fig. 2).

Among the top hits with nominal *P* values < 0.05 in l-Dopa^negative^ IPD patients, which did not show up in l-Dopa^positive^ individuals, we found several membrane lipids such as lysophosphatidic acids (16:0 and 14:0), phosphatidylcholines (36:4, 38:7, and 0 to 36:5), and phosphoethanolamines (34:2 and 36:4). Furthermore, we found the endocannabinoids arachidonoyl glycerol (1-AG/2-AG) and linoleoyl glycerol (1-LG/2-LG) to be lowered in l-Dopa^negative^ IPD patients. Notably our methods did not allow us to distinguish between 1-AG and 2-AG as well as 1-LG and 2-LG, respectively. In addition, we found triglycerides (50:1, 52:1, 54:7, and 56:6), glutathione, citric acid, and thromboxane B2 to be elevated. Three acetyl-carnitines were elevated (hexadecenoylcarnitine, oleoylcarnitine, and tetradecenoylcarnitine), whereas one was lowered (tiglylcarnitine) (Table 4 and table S9).

**Table 4.** Metabolites differently expressed in patients with IPD without l-Dopa treatment (l-Dopa^negative^; *n* = 30) compared to HCs (*n* = 64). Shown are the results of the fixed effect meta-analysis with a nominal *P* value below 0.05, regression coefficient (CI: 95% confidence interval), nominal and adjusted *P* values, sorted by adjusted *P* value. The complete table with all metabolites can be found in table S9.

Name	*z* value	Nominal *P*	Adjusted *P*	β (CI)
Lysophosphatidic acid (16:0)	−3.418	0.000631	0.0959	−0.685 (−1.078; −0.292)
1-Arachidonoyl glycerol/2-arachidonoyl glycerol	−3.589	0.000332	0.0959	−0.762 (−1.178; −0.346)
Glutathione	2.751	0.00594	0.301	0.432 (0.124; 0.74)
1-Linoleoyl glycerol (18:2)/2-linoleoyl glycerol (18:2)	−2.766	0.00567	0.301	−0.573 (−0.978; −0.167)
Phosphatidylcholine (36:4)	−2.858	0.00426	0.301	−0.634 (−1.069; −0.199)
Phosphatidylcholine (38:7)	−2.764	0.0057	0.301	−0.571 (−0.975; −0.166)
Citric acid	2.618	0.00885	0.384	0.57 (0.143; 0.997)
Hexadecenoylcarnitine	2.532	0.0114	0.432	0.533 (0.12; 0.946)
Phosphatidylethanolamine (34:2)	−2.458	0.014	0.449	−0.606 (−1.089; −0.123)
Phosphatidylethanolamine (36:4)	−2.438	0.0148	0.449	−0.58 (−1.046; −0.114)
Oleoylcarnitine	2.383	0.0172	0.474	0.505 (0.09; 0.92)
Tiglylcarnitine	−2.321	0.0203	0.514	−0.61 (−1.126; −0.095)
Putrescine	2.227	0.026	0.56	0.386 (0.046; 0.725)
Lysophosphatidic acid (14:0)	−2.107	0.0351	0.56	−0.398 (−0.768; −0.028)
Glycoursodeoxycholic acid	−2.117	0.0342	0.56	−0.449 (−0.865; −0.033)
Thromboxane B2	−2.132	0.033	0.56	−0.374 (−0.718; −0.03)
Phosphatidylcholine (O-36:5)	−2.088	0.0368	0.56	−0.505 (−0.978; −0.031)
Triglyceride (50:1)	−2.195	0.0282	0.56	−0.49 (−0.928; −0.052)
Triglyceride (52:1)	−2.176	0.0296	0.56	−0.49 (−0.932; −0.049)
Triglyceride (54:7)	−2.154	0.0313	0.56	−0.515 (−0.984; −0.046)
Oleic acid	2.024	0.043	0.623	0.486 (0.015; 0.956)
Tetradecenoylcarnitine	1.963	0.0497	0.653	0.445 (0.001; 0.89)
Triglyceride (56:6)	−1.968	0.049	0.653	−0.478 (−0.955; −0.002)

### Disease duration and disease severity in l-Dopa^negative^ IPD patients are associated with alterations in different metabolite classes

**Fig. 3.**
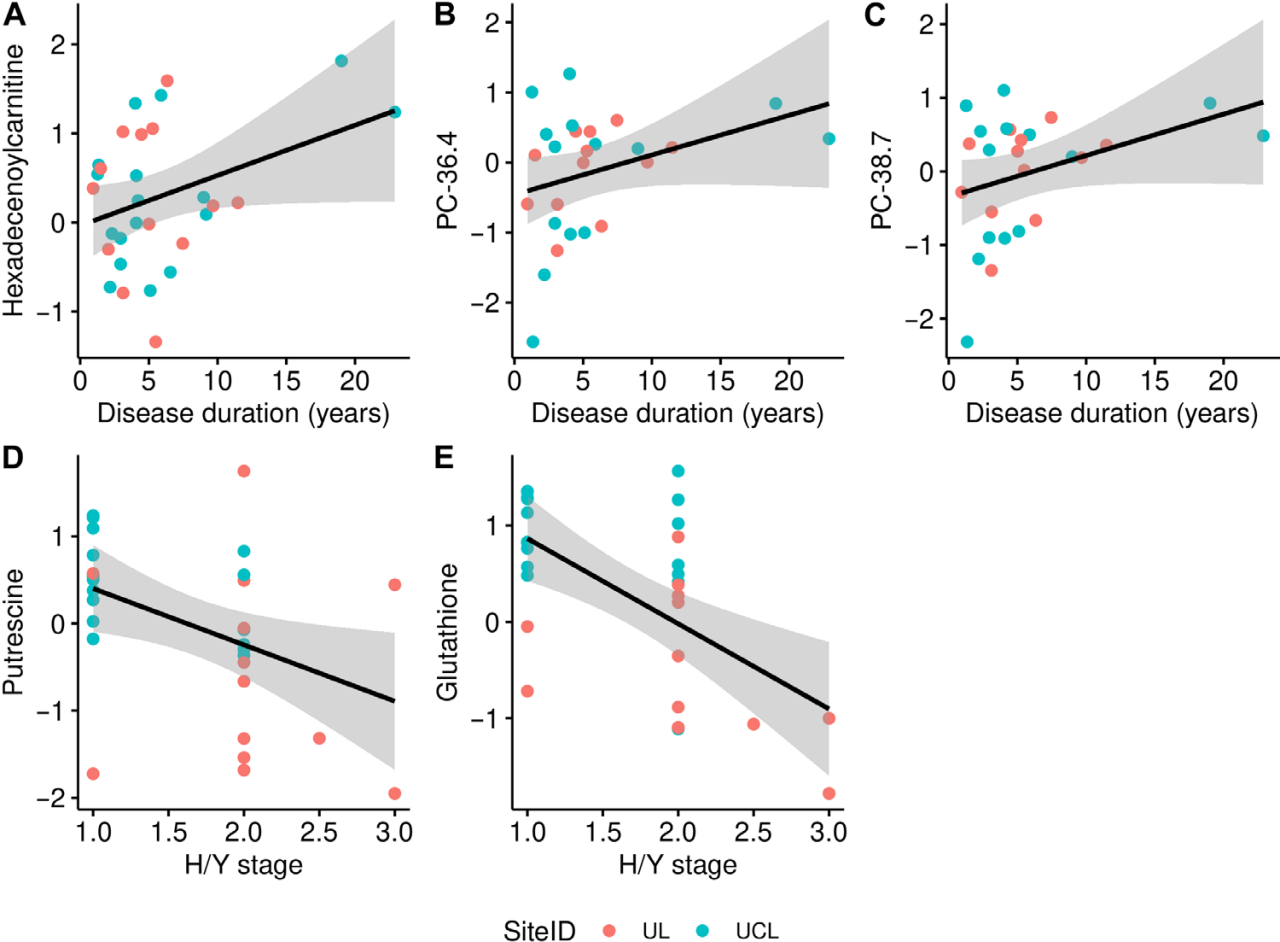
Exploratory associations of metabolite levels with disease duration and disease severity, measured by H/Y staging, in patients with IPD not treated with l-Dopa (l-Dopa^negative^). Three metabolite levels were exploratively associated with disease duration (**A** to **C**) and two with H/Y staging (**D** and **E**) as indicated by a nominal *P* < 0.05. Note that the regression line is descriptive and does not correspond to the regression coefficients based on the meta-analysis. The figures reflect the data and the calculations depicted in tables S7 and S8.

We conducted explorative regression analyses to investigate whether there was a nominal association between the discovered altered metabolites and disease duration and disease severity in the l-Dopa^negative^ group. Regarding carnitines, we observed an exploratory association between disease duration and hexadoconylcarnitine (nominal *P* value < 0.05) (Fig. 3A), but no association between carnitines and Hoehn and Yahr (H/Y) scores.

With respect to lipids, higher levels of two phosphatidylcholines (PC 36.4 and PC 38.7) were associated with longer disease duration (nominal *P* value < 0.05) (Fig. 3, B and C). Last, we found a negative association between putrescine and glutathione and disease severity (nominal *P* value < 0.05) (Fig. 3, D and E), but no association with disease duration.

None of the other metabolites showed any significant association with H/Y stage or disease duration upon explorative regression analysis. The association between all investigated metabolites and disease duration and H/Y stage is shown in tables S10 and S11.

### In the mitoPD group, hydroxyeicosatetraenoic acids (HETEs) are elevated, which were not found to be altered in patients with IPD besides likewise increased l-Dopa–dependent metabolites

Most of the patients with mitoPD (27 of 30) were treated with l-Dopa, thus investigating a l-Dopa^negative^ group was not possible. Again, the l-Dopa–dependent metabolites methyldopa [regression coefficient = 1.95 (CI: 1873 to 2026); adjusted *P* < 0.0001] and methoxytyramine [regression coefficient = 1571 (1339 to 1804); adjusted *P* < 0.0001] were elevated in the mitoPD group compared to HC after adjusting for multiple testing. Within metabolites with nominal *P* values < 0.05, we found among the top hits not only 16-HDoHE, l-tyrosine, putrescine, and ornithine but also 5-HETE, 8-HETE, 11-HETE, and 15(S)-HETrE (table S12). Association analyses comparing metabolite levels and disease duration and severity in patients with mitoPD were not performed due to the small sample size.

## DISCUSSION

Our study demonstrates (i) that l-Dopa treatment substantially affects the metabolomic profile in PD and (ii) that levels of specific lipids and endocannabinoids were different between patients with PD and controls even after confounding effects of l-Dopa therapy were excluded. Our approach of differentiating between l-Dopa and agonist treatment allows new insights into the PD metabolome, implicating previous studies’ interpretation and future metabolomics study design in PD.

Although preanalytical sample handling was strictly harmonized as described above, we still observed variability in metabolite levels between the two study sites. The distinct reasons for this variability are not entirely clear. However, variability might be influenced by differences in diet (*16*); lifestyle factors, including physical activity and comorbidities (*17*, *18*); and the gut microbiome (*19*) between participants. We also cannot fully exclude variations during temperature-controlled shipment from the different study sites to the institution where metabolomic measurements were performed. This variability must be considered in multicenter metabolomic studies in general. However, it can be addressed by appropriate statistical methods, as demonstrated in our study by the performed meta-analysis approach.

### l-Dopa treatment

Many previous PD metabolomic studies have highlighted several metabolites as promising candidates for differentiating HC from patients with PD. However, by differentiating between l-Dopa and agonist treatment/no dopaminergic medication, we found several of these metabolites were increased by l-Dopa treatment itself, but not by agonist treatment.

Thus, we have addressed a major challenge faced in studies of the PD metabolism, namely, that most of the patients included are already undergoing treatment with l-Dopa. Expectedly, methyldopa and methoxytyramine, both breakdown products of l-Dopa, were highly elevated in patients with l-Dopa–treated IPD and mitoPD. They were neither elevated in patients with IPD without l-Dopa treatment nor in patients with l-Dopa agonist treatment only. As expected, the LEDD and the daily l-Dopa dose correlated with levels of methyldopa and methoxytyramine in the blood.

In addition, we found 16-HDoHE, a very long-chain fatty acid, to be significantly elevated in l-Dopa–treated patients. Higher levels of 16-HDoHE were also weakly associated with longer disease duration. This suggests that this metabolite is partly influenced by l-Dopa intake and, at the same time, affected by disease duration. 16-HDoHE is a type of hydroxyeicosatetraenoic acid (HETE) that is derived from arachidonic acid (*20*). Both arachidonic acid and docosahexaenoic acids are polyunsaturated fatty acids present in phospholipids of membranes of the body’s cells and are abundant in the brain. HETEs are markers of oxidative damage and are increased in IPD (*21*).

α-Syn has been reported to immediately change its structure in the presence of both arachidonic acid and docosahexaenoic acids to its α helical conformation. Upon prolonged exposure to docosahexaenoic acids, α-syn gradually assembles into amyloid-like fibrils, with the docosahexaenoic acid being part of the aggregate (*10*, *22*).

However, 16-HDoHE did not cluster with its downstream products or other arachidonic acid derivatives such as other hydroxydocosahexaenoic acids in our study. It had similar levels in the plasma of non–l-Dopa–treated participants and HC. Therefore, the role of 16-HDoHE in the PD metabolome is unclear based on our data. Otherwise, HETEs were among the top hits of altered metabolites investigating patients with mitoPD, which are, however, entirely treated with l-Dopa, potentially supporting the role of l-Dopa treatment on this specific metabolite pathway.

### Polyamine metabolism

Alterations in the ornithine metabolism, its downstream metabolite urea (*23*), and polyamine metabolites such as putrescine and spermidine have been repeatedly suggested as a marker of PD state and severity (*24*, *25*), even when controlling for LEDD (*26*). However, only one of the studies (*25*) did include a small group (*n* = 7) of non–l-Dopa–treated probands. In our study, ornithine and putrescine were also elevated in the l-Dopa^positive^ group. Regarding non–l-Dopa–treated individuals, only putrescine was elevated when compared with HC, and only when considering nominal *P* values. Thus, our data imply that polyamine levels in PD are strongly influenced by l-Dopa treatment. In line with this suggested link between l-Dopa–treatment and urea metabolism, putrescine is significantly elevated in rat brains and livers after oral administration of l-Dopa (*27*). We revealed a negative association between putrescine levels and disease severity (H/Y stage) in l-Dopa^negative^ patients. This finding could indicate that the disease and the l-Dopa treatment influence the polyamine metabolism. It is tempting to speculate that there is some compensatory up-regulation of the polyamine metabolism at the beginning of the disease that decreases in advanced stages. However, there was no association with disease duration. Therefore, the biological underpinnings of a potential relationship between l-Dopa treatment, PD, and polyamine metabolism needs further investigation.

### Endocannabinoid metabolism

2-AG is the brain’s most abundant endocannabinoid and the primary ligand to the cannabinoid type 1 (CB1) receptor. Another prominent cannabinoid is 2-LG, a partial agonist to the CB1 receptor (*28*). Both regulate diverse neural functions and are fundamental to synaptic plasticity. Endocannabinoids are released in the synapse via a synuclein-dependent mechanism, which is not functional in conditions with misfolded α-syn (*29*). Homozygous loss-of-function mutations in 2-AG synthase diacylglycerol lipase β that produce 2-AG and 2-LG have been linked to early-onset autosomal recessive parkinsonism (*30*). In addition, increasing 2-AG levels is neuroprotective in the MPTP-mouse model of PD (*31*).

In agreement with these findings and another small study that measured only 2-AG (*32*), we found markedly reduced levels of 2-AG and 2-LG in l-Dopa^negative^ IPD patients. In patients with mitoPD, endocannabinoid levels were not reduced, which could indicate a less severe disruption of the endocannabinoid system, which might be because there is less α-syn involved. The treatment of patients with PD with medical cannabis has so far yielded controversial results and needs further investigation with endocannabinoids as potential biomarkers (*33*).

### Lipid metabolism and crosslink between fatty acids and α-syn

Many studies have indicated that fatty acid metabolism is altered in patients with IPD, and several membrane lipids such as sphingolipids and glycerophospholipids (*13*) have been consistently reported to be decreased in PD (*4*, *10*). Notably, several genes in the metabolomic pathway of these lipids are associated with PD, such as *PLA2G6*, *SMPD1,* and *GBA* (*13*). There is also a well-established interaction between α-syn and docosahexaenoic acids, as discussed above (*10*). α-Syn also interacts with other lipids, such as sphingolipids, and its pathway follows the neurotoxic process after the aggregation of α-syn (*34*).

We found several lipids decreased in our study comparing l-Dopa^negative^ IPD patients with HC. Most of these metabolites belonged to membrane-associated lipids such as sphingolipids, glycerophospholipids, and triglycerides. In detail, we identified lower levels of lysophosphatidic acid (14:0 and 16:0) that are upstream metabolites forming various other glycerophospholipids and three phosphatidylcholines (PC 36.4, PC O36.5, and PC 38.7). We found longer disease duration to be nominally associated with increased levels of two of these phosphatidylcholines (PC 36.4 and PC 38.7).

PLA2G6 converts lysophosphatidylcholines into phosphatidylcholines, and lipid analysis of brain tissues has revealed that the acyl chain length of phospholipids is shortened by *PLA2G6* loss, which causes endoplasmic reticulum stress through membrane lipid disequilibrium that, in turn, leads to dopaminergic neurodegeneration (*35*). Lysophosphatidylcholines and lysophosphatidylethanolamine have been found to strongly inhibit α-syn aggregation (*36*). We also identified lower levels in two phosphatidylethanolamines (34:2 and 36:4), which are downstream metabolites of phosphatidylcholines, which confirms previous findings (*13*).

We detected several triglycerides to be lowered in l-Dopa^negative^ IPD patients. The relationship between triglycerides and PD is controversial, as a recent meta-analysis claimed a protective effect of elevated levels of triglycerides in PD (*37*). In contrast, another meta-analysis in the same year found no effect (*38*). Because the number of complex lipids is lowered, as discussed above, low levels of simple lipids such as triglycerides could also result from transformation into complex lipids.

Together, the evidence provided by our data, demonstrating alterations in a cluster of lipids acting in a common pathway, excluding the influence of l-Dopa treatment, underscores the potential pathophysiological role of these metabolites in PD, mediated by a possible crosslink with α-syn.

### Other metabolites

Acylcarnitines transport acyl groups (organic acids and fatty acids) from the cytoplasm into the mitochondria, where they are broken down to produce energy via β-oxidation. We found three acylcarnitines to be increased (hexadecenoylcarnitine, tetradecenoylcarnitine, and oleoylcarnitine) and one (tiglylcarnitine) to be lowered in l-Dopa^negative^ IPD patients. Previous studies had suggested lower levels of free and total carnitine levels in older individuals (*5*) and in healthy individuals compared to patients with IPD. However, no drug naïve patients had been included (*39*, *40*). Our analyses revealed no clear association of acylcarnitines with disease severity. However, explorative analyses revealed a potential association between increased acylcarnitine levels and disease duration.

Furthermore, we found glutathione, the most abundant and important antioxidant in the human body, to be increased in l-Dopa^negative^ IPD patients. This finding is in contrast to previous research that found lower levels of glutathione in the substantia nigra of patients with IPD (*41*). A recent study suggested that an increase in plasma glutathione was associated with less increase in PD probability (*42*), which aligns with our finding of a negative association between glutathione levels and clinical severity. However, clinical trials had not shown glutathione treatment to be superior to placebo regarding the alleviation of PD symptoms (*43*, *44*).

We found reduced glycoursodeoxycholic acid levels in l-Dopa^negative^ IPD patients based on nominal *P* values. While glycoursodeoxycholic acid has not yet been linked to PD, it was found to be increased in patients with type 2 diabetes and has been discussed as a marker for hyperglycemia (*45*). Given the high prevalence of type 2 diabetes among individuals with PD (*46*), we consider this finding highly credible.

Last, citric acid was found to be elevated in l-Dopa^negative^ IPD patients based on the nominal *P* value. No study to date suggests increased citric acid in PD, but we found a strong connection of its elevation with age at examination, which has widely been reported. Accumulation of citric acid, the starting point of the citric acid circle, could also indicate a shift from oxidative phosphorylation to increased glycolysis due to mitochondrial dysfunction, which we previously described (*47*, *48*).

### MitoPD

Only one study investigated the metabolome in *PRKN*-linked PD (*49*), and no investigations addressing patients with *PINK1*-linked PD have been available so far. We combined these two autosomal recessively inherited forms of PD, as the proteins encoded by these genes act in a common biochemical pathway, mainly involved in the degradation of damaged mitochondria (*8*), but links to changes in the innate immune system have also been described to be associated with PRKN and PINK1 dysfunction (*50*). We were able to partly reproduce the differences described in the only other metabolomics study investigating patients with *PRKN*-linked PD, where an elevation of fatty acids and oxidized lipids and a decrease of antioxidants, caffeine, and benzoate-related metabolites were reported. We found several hydroxyeicosatetraenoic acids [5-HETE, 8-HETE, 11-HETE, and 15(S)-HETrE] elevated at nominal *P* values. These metabolites were not found to be different in the comparison between IPD and HC. As explained above, this might reflect different pathways of oxidative dysfunction in these subsets of patients with PD known to display mitochondrial alterations. This idea of different pathophysiological pathways in monogenic and IPD (*51*), as well as different mechanisms such as mitochondrial versus lysosomal dysfunction within idiopathic PD (*52*), is well-established and should be considered in potential disease-modifying trials. However, as almost all patients with *PRKN/PINK1*-linked PD in this study were treated with l-Dopa, conclusions drawn from these analyses are limited due to the substantial effect of l-Dopa treatment on the metabolome. Methoxytyrosine and methyldopa were the only metabolites that were significantly different between the mitoPD group and HC. Furthermore, most metabolites with nominal *P* values < 0.05—such as (+/−) 16-HDoHE, l-tyrosine, putrescine, and ornithine—were identified as l-Dopa–dependent within our study. Thus, investigating the mitoPD group did not allow us to draw definite conclusions, as almost all patients were treated with l-Dopa, and the sample size was too small.

### Strengths and limitations

The strengths of our study consist of considering the substantial effect of l-Dopa medication and investigating two independent samples of probands recruited with the same detailed protocol to ensure identical preanalytical handling. Nonetheless, we observed site-dependent effects. Thus, we applied a strict statistical approach to correct for this bias and for multiple testing. Furthermore, we used state-of-the-art large-scale comprehensive metabolomic platforms to measure more than 300 metabolites and identify differences in single metabolites rather than in clusters.

Although our statistical approach includes replication of metabolites at both study sites, some of our findings must be considered preliminary, as they formally only reach nominal significance and, therefore, require replication in independent studies. Moreover, relevant effects in the l-Dopa^negative^ group may have been missed due to the sample size of *n* = 30. Further limitations include the cross-sectional study design and the relatively small sample size regarding patients with *PRKN-/PINK1*-linked disease, making association analysis with disease severity impossible. Moreover, no group of patients with pathogenic biallelic variants in *PRKN* and *PINK1* without l-Dopa treatment was available, preventing deeper insight into the effects of dopaminergic medication on the metabolome in this group of patients with PD. In addition, to investigate the detected alterations as potential biomarkers for PD, a comparison with atypical PD forms, such as progressive supranuclear palsy and multiple system atrophy, would be required in future studies.

In conclusion, we investigated a broad range of more than 300 metabolites that covered all major biochemical pathways. Adjusting for multiple testing, only metabolites affected by l-Dopa treatment significantly differed, highlighting the dominant effect of l-Dopa on the PD metabolome. However, several metabolites had nominal *P* values < 0.05 comparing HCs and non–l-Dopa–treated PD patients, which we consider relevant as (i) applying strict statistical correction for multiple testing might mitigate all but significant effects if such a magnitude of tests are performed; (ii) several of these metabolites have been consistently reproduced in other studies as we recently reviewed (*4*); (iii) several of these metabolites belong to the same biological pathways, making a role in PD metabolism more plausible; and (iv) some of the relevant metabolites were associated with disease state and progression, further highlighting their biological impact. Of special importance, our statistical approach directly includes the replication of our findings in two independent cohorts, representing a crucial step that was lacking in most of the PD metabolomics studies performed so far (*4*).

Overall, our study underscores the large impact of l-Dopa treatment on metabolomic studies in PD. Thus, we highlight the risk of misinterpreting differences between patients with IPD and HCs as disease specific, when in fact they may simply relate to l-Dopa treatment. Thus, the impact of dopaminergic treatment should be considered in all future metabolomics studies. This is also relevant for biomarker studies dealing with PD in general, as with recent findings implicate that l-Dopa treatment even affects PD imaging biomarkers (*53*). Furthermore, our association analyses revealed the evidence of different metabolite trajectories throughout the disease; however, longitudinal metabolomic studies are warranted to assess relevant metabolite changes over the disease course. Last, we found evidence for the role of specific glycerophospholipids and endocannabinoids in disease progression and severity, supporting recent findings that lipid pathway alterations influence PD pathogenesis.

## MATERIALS AND METHODS

### The SysMedPD study

The Systems Medicine of Mitochondrial PD (SysMedPD) study (www.sysmedpd.eu) was initiated by an international consortium, including basic and clinician scientists, to contribute to the elucidation of mitochondrial underpinnings of PD. The consortium consisted of eight partners from five European countries. At the same time, recruitment of patients with idiopathic and different forms of monogenic PD, as well as healthy controls, took place at the Institute of Neurogenetics at the University of Lübeck (UL) and the Department of Neurology at the UCL. Before recruitment, inclusion and exclusion criteria, the clinical work-up, and the sampling of biomaterials were harmonized between both centers.

### Ethics statement

The study was approved by the Ethics Committee of the University of Lübeck (study number 16-195) and London (REC reference number 17/LO/1166). Written informed consent was obtained from all participants. The study was performed in accordance with the Declaration of Helsinki.

### Proband recruitment

**Fig. 4.**
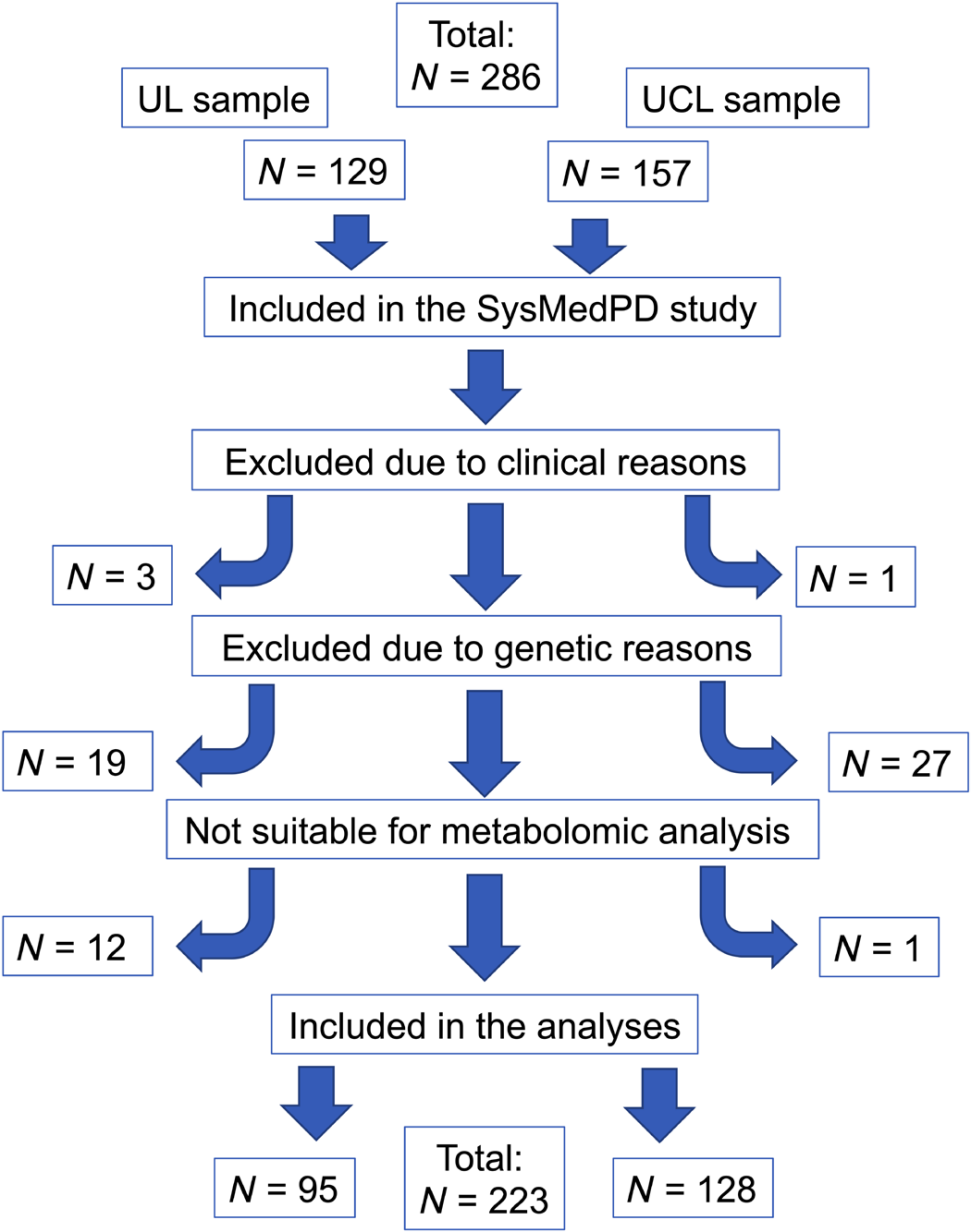
Flowchart of the exclusion and inclusion process. UCL, University College London; UL, University of Lübeck. Excluded due to clinical reasons: Patients who do not fulfill the diagnostic criteria of PD as described in the methods section. Excluded due to genetic reasons: UL: Patients were excluded due to pathogenic variants/risk variants in *GBA1* (*n* = 10), *LRRK2* (*n* = 2), *SNCA* (*n* = 1), heterozygous variants in *PRKN* or *PINK1* (*n* = 4), and nonconclusive genetic results (*n* = 2). UCL: Patients were excluded due to pathogenic variants/risk variants in *GBA1* (*n* = 12), *LRRK2* (*n* = 7), heterozygous variants in *PRKN* or *PINK1* (*n* = 6), and nonconclusive genetic results (*n* = 2). Patients were not suitable for metabolomic analyses if biomaterials to perform the analyses were not available.

The total sample at the two recruitment sites comprised 289 participants. Inclusion criteria for affected individuals consisted of a definite diagnosis of PD according to the MDS clinical diagnostic criteria for PD (*54*), eligibility according to the harmonized genetic criteria (see below), and material availability to perform metabolomics analyses. Otherwise, patients with clinical suspicion of atypical PD, coexisting neurodegenerative disorders, diagnosed dementia, or a history of severe infection (defined by the need for antibiotics) or surgery in 2 weeks before participation were excluded. A flowchart providing the selection process for inclusion in the study is depicted in Fig. 4. All participants underwent an in-depth clinical assessment for motor symptoms and clinical severity using the Movement Disorder Society Unified Parkinson’s Disease Rating Scale III (MDS-UPDRS III) and the H/Y scale. However, because of superior robustness, we only used H/Y scores for exploratory association analyses. Moreover, the LEDD was calculated as published (*55*).

### Genetic testing

We recruited patients with known pathogenic variants in *PRKN* and *PINK1* and uncovered priorly unknown genetic alterations by genotyping performed within the study. All participants underwent conclusive genetic testing within the study, which differed slightly between both sides. At UL, all samples underwent gene panel sequencing at CENTOGENE (Rostock, Germany). The panel covered the genes *PRKN*, *PINK1*, *DJ-1*, *SNCA*, *LRRK2*, *GBA1*, *VPS35*, *GCH1*, and others irrelevant to the present study (*56*). We confirmed potentially disease-related variants by Sanger sequencing. At UCL, the Illumina NeuroChip array, a customized version of the Infinium HumanCore-24 v1.0 backbone, was used, covering nearly 200,000 variants associated with neurodegenerative diseases, including but not limited to PD (*57*). Furthermore, we performed multiplex ligation-dependent probe amplification (MLPA) to investigate copy number variations (i.e., deletions or duplications) in PD-associated genes (*SNCA*, *PRKN*, *PINK1*, *DJ-1*, *LRRK2*, and *GCH1*). We performed sequence analyses and MLPA in patients and control participants at both sites. The pathogenicity of detected variants was assessed according to the ACMG (American College of Medical Genetics and Genomics) (*58*) and the MDSGene (www.mdsgene.org) criteria.

Genetic study inclusion criteria were likewise harmonized between both study sites: (i) We excluded patients with PD and controls with pathogenic variants in known PD genes besides pathogenic biallelic *PRKN* and *PINK1* variants. (ii) We excluded individuals with heterozygous pathogenic variants in *PRKN* and *PINK1* due to conflicting evidence regarding their role in PD (*59*, *60*).

### Study groups

After the exclusion of screened participants due to either clinical criteria (*n* = 4), genetic criteria (*n* = 46), or lack of sufficient biomaterial to perform the experiments (*n* = 13), 223 participants were included. This comprised 140 patients with IPD, 19 patients with *PRKN/PINK1*-linked PD (*PRKN*: *n* = 16 individuals; *PINK1*: *n* = 3) and 64 healthy controls undergoing metabolomic analyses (Fig. 4). Carriers of biallelic pathogenic variants in *PRKN* and *PINK1* formed the “mitoPD” group for all analyses.

### Preanalytic sample processing

Blood samples for metabolomic and genetic analysis were collected from each participant at 8 a.m. in the morning after a 12-hour fasting period, where participants were only allowed to take medication and drink water. Following the venous blood collection, the samples were placed on ice and promptly conveyed to the laboratory within 15 min. The specimens underwent centrifugation at a force of 2000*g* for 10 min at a controlled temperature of 4°C (centrifuges: Beckman Coulter Allegra X-15R and Eppendorf centrifuge 5417R).

Subsequently, the resulting plasma supernatants were transferred into a fresh 15-ml Falcon tube, prechilled on ice, and subjected to thorough mixing through inversion. Following this step, aliquots of 300-ml each were prepared, rapidly cryopreserved by immersion in liquid nitrogen, and ultimately stored at a temperature of −80°C. Specimens from UL and UCL were shipped on dry ice to the University of Leiden under strictly temperature-controlled conditions.

### Metabolomic analyses

Metabolites were assessed with five different metabolite platforms that are described below:

1) The amine platform (*61*) covers amino acids and biogenic amines using an AccQ-Tag derivatization strategy adapted from the protocol supplied by Waters. A 5.0 μl of each sample was spiked with an internal standard solution. Then, proteins were precipitated by adding MeOH and taken to dryness in a SpeedVac centrifuge. The residue was reconstituted in borate buffer (pH 8.8) with AQC reagent. A 1.0 μl of the reaction mixture was injected into the ultra-performance liquid chromatography–mass spectrometry (UPLC-MS)/MS system. Chromatographic separation was achieved by an Agilent 1290 Infinity II LC System on an AccQ-Tag Ultra column (Waters) with a flow of 0.7 ml/min over an 11-min gradient. The UPLC was coupled to electrospray ionization on a triple quadrupole mass spectrometer (AB SCIEX QTRAP 6500). Analytes were detected in the positive ion mode and monitored in multiple reaction monitoring (MRM) using nominal mass resolution. Acquired data were evaluated using MultiQuant Software for Quantitative Analysis (AB SCIEX, version 3.0.2) by integrating assigned MRM peaks and normalizing using internal standards.

2) The acylcarnitine platform covers acylcarnitines and trimethylamine *N*-oxide, choline, betaine, and carnitine. Ten microliter of each sample was spiked with an internal standard solution, and proteins were precipitated by adding MeOH. The supernatant was transferred to an autosampler vial, and 1.0 μl was injected into the UPLC-MS/MS. Chromatographic separation was achieved by an Agilent 1290 Infinity II LC System on an Accq-Tag Ultra column (Waters) with a flow of 0.7 ml/min over an 11-min gradient. The UPLC was coupled to electrospray ionization on a triple quadrupole mass spectrometer (AB SCIEX Qtrap 6500). Analytes were detected in the positive ion mode and monitored in MRM using nominal mass resolution. Acquired data were evaluated using MultiQuant Software for Quantitative Analysis (AB SCIEX, version 3.0.2) by integrating assigned MRM peaks and normalizing using the internal standards.

3) The organic acid profiling platform, performed with gas chromatography–MS (GC-MS) technology, covers 28 organic acids. Sample preparation was done by adding the first protein precipitation of 50 μl of plasma with MeOH/H_2_O with internal standard. After centrifugation and transferring the supernatant, the samples were evaporated to complete dryness in the SpeedVac. Then, two-step derivatization procedures were performed online: oximation using methoxyamine hydrochloride (MeOX, 15 mg/ml in pyridine) as first reaction and silylation using *N*-methyl-*N*-(trimethylsilyl)-trifluoroacetamide as second reaction were carried out. One microliter of each sample was injected into the GC-MS. The metabolites were measured by gas chromatography on an Agilent Technologies 7890A equipped with an Agilent Technologies mass selective detector (MSD 5975C) and MultiPurpose Sampler (MPS, MXY016-02A, GERSTEL). Chromatographic separations were performed on an HP-5MS UI (5% phenyl methyl siloxane), 30 mÅ to 0.25 m internal diameter (ID) column with a film thickness of 25 μm, using helium as the carrier gas at a flow rate of 1.7 ml/min. The mass spectrometer was operated in SCAN mode with a mass range of 50 to 500. Raw data were preprocessed using Agilent MassHunter Quantitative Analysis software (Agilent, Version B.05.01).

4) The signaling lipids platform (*62*) covers various isoprostane classes and their respective prostaglandin isomers from different polyunsaturated fatty acids. Also included in this platform are endocannabinoids, bile acids, and lipids from the sphingosine and sphinganine classes and their phosphorylated forms, as well as three classes of lysophosphatidic acids.

The signaling and peroxidized lipids platform is divided into two chromatographic methods: low and high pH. Each sample was spiked with antioxidant and internal standard solution. The extraction of the compounds is performed via liquid-liquid extraction. Butanol and methyl tert-butyl ether extract the analytes from the aqueous phase. The organic phase is concentrated by drying, reconstituted, transferred into amber autosampler vials, and used for high and low pH injection. For the high pH method, a Kinetex EVO column (Phenomenex) was used on a Shimadzu UPLC system formed by three high-pressure pumps coupled online with an LCMS-8050 triple quadrupole mass spectrometer (Shimadzu). The acquired data were evaluated using LabSolutions Insight software (version 3.3, Shimadzu). The low pH method used an Acquity UPLC BEH C18 column (Waters) on a Shimadzu UPLC system coupled to a QTRAP mass spectrometer (SCIEX). Analytes were monitored in dynamic MRM and evaluated using MultiQuant (version 3.0.2).

5) The lipidomics platform (*63*) covers 185 compounds, including triglycerides, cholesterol esters, and phospholipids. Isopropanol (1000 μl) containing internal standards were added to 10 μl of plasma. A total of 2.5 μl was injected on an HSS T3 column on an ACQUITY UPLC (Waters, Ettenleur, the Netherlands) with a 16-min gradient. The lipid analysis is performed on a UPLC-ESI-Triple-TOF (Sciex 6600+) high-resolution mass spectrometer using reference mass correction. Lipids were detected in full scan in the positive ion mode. MultiQuant Software preprocessed the raw data for Quantitative Analysis (AB SCIEX, Version 3.0.2). The lipid response was calculated as the peak area ratios of the target analyte to the respective internal standard. For all metabolomic platforms, in-house developed algorithms were applied using pooled quality-controlled samples to compensate for shifts in the sensitivity of the mass spectrometer over batches.

### Statistics

Metabolite data were further preprocessed and quality controlled. If a metabolite was available on several platforms, then measurements from the platform with the lowest relative SD based on quality-control samples were kept. Missing values were replaced by a constant metabolic-specific value defined as 0.5 × minimally observed value. A log transformation (base2) was applied to make the distribution of the intensities of each metabolite more symmetric. Following this transformation, the *z* score was calculated for each metabolite to achieve a distribution with a mean of 0 and an SD of 1 using the mean value of intensities of each metabolite divided by the SD, facilitating the comparison of metabolites.

Additional quality control steps included missing frequencies per metabolite and proband, comparison of intensity distributions across subjects, principal components analysis (PCA), and checks for duplicate samples as implemented in the R package QCnormSE (version 0.99.4.9000) available on Zenodo (https://zenodo.org/records/14728213). No metabolites or samples were excluded due to quality issues.

However, we observed a strong batch effect of recruitment sites on the overall distribution of the metabolite intensities in the PCA plots, especially for amines and lipid metabolites. Thus, we first applied a linear regression analysis for each metabolite separately, with transformed metabolite intensity as the dependent variable and recruitment site as the variable of interest and adjusted for age at examination, sex, and diagnosis group (IPD or control) as independent variables. *P* values for the effect of the recruitment site were adjusted for multiple testing using the Benjamini-Hochberg procedure (*64*). Because many metabolites showed a significant association, we conducted all further association analyses of metabolite intensities with clinical variables stratified by recruitment site. Site-specific results containing the mean metabolite intensities in each group and the results of the linear regression analysis with metabolite intensity as the dependent variable and the group, sex, and age as independent variables (regression coefficient for group, SE, and nominal and adjusted *P* values) are shown in tables S7 and S8.

Again, we used a linear regression model for each metabolite separately, with transformed metabolite intensity as the dependent variable and the variable of interest (categorical or continuous) as the independent variable and included additional covariates. Details on the participants and covariates included in each analysis and the corresponding tables and figures can be found in table S13. In particular, most of the analyses were adjusted for age at examination. However, we did not adjust for age at examination when analyzing disease severity measured as H/Y stage because of the substantial overlap between increasing age and disease severity in patients with PD that is similar to the overlap of PD status and levodopa treatment. Therefore, metabolites associated with age at examination and disease severity will be separately discussed.

We used a meta-analysis approach to combine the results of the two recruitment sites, which has several substantial advantages compared to a two-stage design with a discovery and replication cohort. It provides a more comprehensive and objective assessment of the evidence, greater statistical power, and more precise estimates (*65*) without the necessity to specify the criteria for selecting metabolites for replication and declaring successful replication in advance (*66*). In particular, the risk of false positive findings is substantially decreased because similar effects in both studies must be observed for each metabolite to be called statistically significant. In addition, the risk of false-negative results is also reduced because all individuals are used for identification compared to the smaller sample size in the discovery cohort.

Regression coefficients (β) and SEs of the variable of interest estimated for the two recruitment sites were combined in a meta-analysis using the R package metafor (version 3.8-1) (*67*). We used a fixed-effects meta-analysis because a random-effects meta-analysis is not recommended with two studies (*68*), and the same protocols for recruitment, clinical characterization, and sample collection were used at the two sites. *P* values were adjusted for multiple testing using the Benjamini-Hochberg procedure (*64*). In the results section, we clearly distinguish between significant findings (adjusted *P* value < 0.05) and nominally significant results (nominal = unadjusted *P* value < 0.05) for some explorative analyses. In both cases, we report the meta-analysis’s regression coefficients (β) with corresponding 95% CIs in Results. In addition, we generated forest plots for the main findings (fig. S1), which show the fixed-effects regression coefficients together with information on the heterogeneity of effects between studies. In particular, we report the results of Cochran’s *Q* test (*69*) and the heterogeneity statistic *I*^2^ using the DerSimonian-Laird estimator of τ^2^ (*70*).

We conducted a discovery/replication study to compare it to our preferred meta-analysis approach. We selected the UCL dataset as the discovery cohort because it contained more patients with IPD and controls. We used the comparison of patients with l-Dopa^positive^ versus controls. Metabolites were selected for the replication stage if the nominal *P* value was <0.05 in the discovery cohort. The *P* values in the replication cohort were adjusted for multiple testing using the Benjamini-Hochberg procedure (tables S6 to S8) (*64*).
