# Supplementary material for: The role of dopaminergic medication and specific pathway alterations in idiopathic and PRKN/PINK1-mediated Parkinson’s disease
Source: Sci Adv. 2025 May 14;11(20):eadp7063. doi: 10.1126/sciadv.adp7063 (PMC12077494; doi:10.1126/sciadv.adp7063)
Supplement: Supplementary file 1 — Tables S1 to S13 Fig. S1 [file sciadv.adp7063_sm.pdf]

Science Advances
AAAS

Supplementary Materials for

The role of dopaminergic medication and specific pathway alterations in idiopathic and *PRKN/PINK1*-mediated Parkinson's disease

Alexander Balck *et al.*

Corresponding author: Christine Klein, christine.klein@uni-luebeck.de;
Silke Szymczak, silke.szymczak@uni-luebeck.de

Sci. Adv. **11**, eadp7063 (2025)
DOI: 10.1126/sciadv.adp7063

This PDF file includes:

Tables S1 to S13
Fig. S1

Table S1. Overview over all measured metabolites (n=304) and their respective identifiers

Metabolite Name	ChEBI ID	PubChem_ID	Lipidmaps
(+/-) 10-HDoHE	72640	11537494	LMFA04000027
(+/-) 11-HDoHE	72794	11631564	LMFA04000028
(+/-) 14-HDoHE	72647	11566378	LMFA04000030
(+/-) 16-HDoHE	72613	11595378	LMFA04000031
(+/-) 8-HDoHE	72610	11976798	LMFA04000026
1-AG/2-AG	34071;52392	16019980;5282280	LMGL01010032;LMGL01010023
1-LG/2-LG	75561;75457	5283469;5365676	LMGL01010006;LMGL01010033
1-Methylhistidine	70958	NA	NA
11-HETE	72606	14123410	LMFA03060085
11,12-DiHETrE	63969	5283146	LMFA03050008
12-HETE	8447	13786989	LMFA03060088
12,13-DiHODE	88461	16061067	LMFA02000046
12,13-DiHOME	72665	10236635	LMFA02000230
12,13-EpOME	38229	5356421	LMFA02000038
12(S)-HEPE	72645	10041593	LMFA03070031
13-HODE	72639	5282947	LMFA02000154
14,15-DiHETE	88459	16061119	LMFA03060077
14,15-DiHETrE	63966	5283147	LMFA03050010
15(S)-HETrE	88348	5283145	LMFA03050007
17,18-DiHETE	88349	16061120	LMFA03060078
19,20-DiHDPA	72657	16061148	LMFA04000043
2-Ketoglutaric acid	30915	NA	NA
2-Methylbutyroylcarnitine	73026	NA	NA
3-Hydroxybutyric acid	20067	NA	NA
3-Hydroxypropionic acid	33404	NA	NA
3-Methoxytyrosine	1582	NA	NA
5-HETE	28209	9862886	LMFA03060084
5,6-DiHETrE	63974	5283142	LMFA03050004
8-HETE	72643	11976122	LMFA03060086
8,12-iPF2a IV	NA	156593941	NA
8,9-DiHETrE	63970	5283144	LMFA03050006
8(9)-EpETrE	34490	5283203	LMFA03080003
9-HODE	72651	5282944	LMFA02000151
9-HOTrE	80447	6439873	LMFA02000024
9,10-DiHOME	72663	9966640	LMFA02000229
9,10,13-TriHOME	34499	5282965	LMFA02000168
9,12,13-TriHOME	34506	9858729	LMFA02000014
Acetylcarnitine	73024	NA	NA
Anandamide	2700	5281969	LMFA08040001
Betaine	41139	NA	NA
Butyrylcarnitine	7676	NA	NA

Carnitine	17126	NA	NA
CE(18:1)	NA	NA	NA
CE(18:2)	NA	NA	NA
CE(20:4)	NA	NA	NA
CE(22:6)	NA	NA	NA
Cer(d18:0/22:0)	NA	NA	NA
Cer(d18:0/23:0)	NA	NA	NA
Cer(d18:1/22:0)	NA	NA	NA
Cer(d18:1/23:0)	NA	NA	NA
Cer(d18:1/24:1)	NA	NA	NA
Cholic acid	16359	221493	LMST04010001
Choline	15354	NA	NA
Citric acid	30769	NA	NA
Citrulline	18211	NA	NA
Cortisol	17650	5754	LMST02030001
Cystathionine	15901	NA	NA
Cysteine	15356	NA	NA
Decanoylcarnitine	68830	NA	NA
Decenoylcarnitine	86063	NA	NA
Dehydroepiandrosterone	85252	5283451	LMFA08040009
Deoxycarnitine	16244	NA	NA
Deoxycholic acid	28834	222528	LMST04010040
DG(36:2)	NA	NA	NA
DG(36:3)	NA	NA	NA
DGLEA	34488	5282272	LMFA08040011
DL-3-aminoisobutyric acid	27389	NA	NA
Dodecenoylcarnitine	86065	NA	NA
Ethanolamine	16000	NA	NA
FA 18(1)	16196	445639	LMFA01030002
FA 18(2)	17351	5280450	LMFA01030120
FA 22(5) w6	65136	6441454	LMFA04000064
FA 22(6)	28125	445580	LMFA01030185
Gamma-aminobutyric acid	16865	NA	NA
Gamma-Glutamylglutamine	73707	NA	NA
Gamma-L-glutamyl-L-alanine	50619	NA	NA
Glutathione	16856	NA	NA
Glycine	15428	NA	NA
Glycochenodeoxycholic acid	36274	12544	LMST05030008
Glycocholic acid	17687	10140	LMST05030001
Glycodeoxycholic acid	27471	3035026	LMST05030006
Glycolithocholic acid	37998	115245	LMST05030009
Glycoursodeoxycholic acid	89929	12310288	LMST05030016
Glycylglycine	17201	NA	NA

Hexadecenoylcarnitine	86032	NA	NA
Hexanoylcarnitine	70749	NA	NA
Homocitrulline	17443	NA	NA
Homocysteine	17230	NA	NA
Hydroxylysine	86498	NA	NA
Isobutyrylcarnitine	73017	NA	NA
Isocitrate	30887	NA	NA
Isovalerylcarnitine	73025	NA	NA
L-2-aminoadipic acid	37024	NA	NA
L-4-hydroxy-proline	20392	NA	NA
L-Alanine	16449	NA	NA
L-Alpha-aminobutyric acid	35621	NA	NA
L-Arginine	29016	NA	NA
L-Asparagine	22653	NA	NA
L-Glutamic acid	18237	NA	NA
L-Glutamine	28300	NA	NA
L-Histidine	27570	NA	NA
L-Homoserine	30653	NA	NA
L-Isoleucine	24898	NA	NA
L-Kynurenine	28683	NA	NA
L-Leucine	25017	NA	NA
L-Lysine	25094	NA	NA
L-Methionine	16811	NA	NA
L-Phenylalanine	28044	NA	NA
L-Proline	26271	NA	NA
L-Serine	17822	NA	NA
L-Threonine	26986	NA	NA
L-Tryptophan	27897	NA	NA
L-Tyrosine	18186	NA	NA
L-Valine	27266	NA	NA
Lactic acid	422	NA	NA
Lauroylcarnitine	73054	NA	NA
Linoleoyl ethanolamide	64032	5283446	LMFA08040004
Linoleylcarnitine	73072	NA	NA
LPS 18(1)	52649	9547099	LMGP03050001
Lysophosphatidic acid 14(0)	62833	9547180	LMGP10050007
Lysophosphatidic acid 16(0)	15799	6419701;53478599	LMGP10050006;LMGP10050042
Lysophosphatidic acid 16(1)	75070	52929751	LMGP10050016
Lysophosphatidic acid 18(1)	62837;78270	5311263;52929749	LMGP10050008;LMGP10050014
Lysophosphatidic acid 18(2)	62834;74330	50990923;53478601	LMGP10050017;LMGP10050044
Lysophosphatidylcholine(14:0)	NA	NA	NA
Lysophosphatidylcholine(16:0)	NA	NA	NA
Lysophosphatidylcholine(16:1)	NA	NA	NA

Lysophosphatidylcholine(18:0)	NA	NA	NA
Lysophosphatidylcholine(18:1)	NA	NA	NA
Lysophosphatidylcholine(18:2)	NA	NA	NA
Lysophosphatidylcholine(18:3)	NA	NA	NA
Lysophosphatidylcholine(20:3)	NA	NA	NA
Lysophosphatidylcholine(20:4)	NA	NA	NA
Lysophosphatidylcholine(20:5)	NA	NA	NA
Lysophosphatidylcholine(22:6)	NA	NA	NA
Lysophosphatidylcholine(O-16:0)	NA	NA	NA
Lysophosphatidylcholine(O-16:1)	NA	NA	NA
Lysophosphatidylcholine(O-18:1)	NA	NA	NA
Lysophosphatidylethanolamine (16:0)	73134;131743	9547069;53480922	LMGP02050002;LMGP02050036
Lysophosphatidylethanolamine (16:1)	145277;145248	52925129;53480923	LMGP02050010;LMGP02050037
Lysophosphatidylethanolamine (20:4)	64395	42607465; 53480936; 53480952;53480937	LMGP02050009;LMGP02050051; LMGP02050067;LMGP02050052
Lysophosphatidylethanolamine (20:5)	145287;145263	52925146;53480938	LMGP02050027;LMGP02050053
Lysophosphatidylethanolamine(18:0)	NA	NA	NA
Lysophosphatidylethanolamine(18:1)	NA	NA	NA
Lysophosphatidylethanolamine(20:4)	NA	NA	NA
Lysophosphatidylethanolamine(22:6)	NA	NA	NA
Lysophosphatidylinositol 16(1)	NA	52928604	LMGP06050009
Lysophosphatidylinositol 18(0)	83054	42607495	LMGP06050004
Lysophosphatidylinositol 18(1)	82753	42607496	LMGP06050005
Lysophosphatidylinositol 18(2)	NA	52928605	LMGP06050010
Lysophosphatidylinositol 20(4)	NA	42607497	LMGP06050006
Malic acid	6650	NA	NA
Malonylcarnitine	73028	NA	NA
Methionine sulfone	132188	NA	NA
Methyldopa	167648	NA	NA
Methylmalonylcarnitine	73031	NA	NA
Myristoilcarnitine	73061	NA	NA
N6,N6,N6-Trimethyl-L-lysine	165870	NA	NA
Nonaylcarnitine	70997	NA	NA
O-Acetyl-L-serine	17981	NA	NA
O-Anandamide	418207	5712057	LMFA00000014
O-Phosphoethanolamine	17553	NA	NA
2-Hydroxybutyric acid	1148	NA	NA
Octanoylcarnitine	73039	NA	NA
Octenoylcarnitine	86052	NA	NA
Oleoylcarnitine	72689	NA	NA
Ornithine	18257	NA	NA

Palmitoyl ethanolamide	71464	4671	LMFA08040013
Palmitoylcarnitine	73067	NA	NA
Phosphatidylcholine(32:0)	NA	NA	NA
Phosphatidylcholine(32:1)	NA	NA	NA
Phosphatidylcholine(32:2)	NA	NA	NA
Phosphatidylcholine(34:1)	NA	NA	NA
Phosphatidylcholine(34:2)	NA	NA	NA
Phosphatidylcholine(34:3)	NA	NA	NA
Phosphatidylcholine(34:4)	NA	NA	NA
Phosphatidylcholine(36:1)	NA	NA	NA
Phosphatidylcholine(36:2)	NA	NA	NA
Phosphatidylcholine(36:3)	NA	NA	NA
Phosphatidylcholine(36:4)	NA	NA	NA
Phosphatidylcholine(36:5)	NA	NA	NA
Phosphatidylcholine(38:2)	NA	NA	NA
Phosphatidylcholine(38:3)	NA	NA	NA
Phosphatidylcholine(38:4)	NA	NA	NA
Phosphatidylcholine(38:5)	NA	NA	NA
Phosphatidylcholine(38:6)	NA	NA	NA
Phosphatidylcholine(38:7)	NA	NA	NA
Phosphatidylcholine(40:5)	NA	NA	NA
Phosphatidylcholine(40:6)	NA	NA	NA
Phosphatidylcholine(40:7)	NA	NA	NA
Phosphatidylcholine(40:8)	NA	NA	NA
Phosphatidylcholine(O-34:1)	NA	NA	NA
Phosphatidylcholine(O-34:2)	NA	NA	NA
Phosphatidylcholine(O-34:3)	NA	NA	NA
Phosphatidylcholine(O-36:3)	NA	NA	NA
Phosphatidylcholine(O-36:4)	NA	NA	NA
Phosphatidylcholine(O-36:5)	NA	NA	NA
Phosphatidylcholine(O-38:4)	NA	NA	NA
Phosphatidylcholine(O-38:5)	NA	NA	NA
Phosphatidylcholine(O-38:6)	NA	NA	NA
Phosphatidylcholine(O-44:5)	NA	NA	NA
Phosphatidylethanolamine(34:2)	NA	NA	NA
Phosphatidylethanolamine(36:4)	NA	NA	NA
Phosphatidylethanolamine(38:2)	NA	NA	NA
Phosphatidylethanolamine(38:4)	NA	NA	NA
Phosphatidylethanolamine(38:6)	NA	NA	NA
Phosphatidylethanolamine(O-36:5)	NA	NA	NA
Phosphatidylethanolamine(O-38:5)	NA	NA	NA
Phosphatidylethanolamine(O-38:7)	NA	NA	NA
Pimelylcarnitine	86084	NA	NA

Propionylcarnitine	28867	NA	NA
Prostaglandin E2	15551	5280360	LMFA03010003
Prostaglandin F2α	15553	5280363	LMFA03010002
Putrescine	17148	NA	NA
Pyroglutamic acid	16010	NA	NA
S-Methylcysteine	45658	NA	NA
Saccharopine	30768	NA	NA
Sarcosine	15611	NA	NA
Serotonine	28790	NA	NA
Sphingomyelin(d18:1/14:0)	NA	NA	NA
Sphingomyelin(d18:1/15:0)	NA	NA	NA
Sphingomyelin(d18:1/16:0)	NA	NA	NA
Sphingomyelin(d18:1/16:1)	NA	NA	NA
Sphingomyelin(d18:1/18:0)	NA	NA	NA
Sphingomyelin(d18:1/18:1)	NA	NA	NA
Sphingomyelin(d18:1/18:2)	NA	NA	NA
Sphingomyelin(d18:1/20:0)	NA	NA	NA
Sphingomyelin(d18:1/20:1)	NA	NA	NA
Sphingomyelin(d18:1/21:0)	NA	NA	NA
Sphingomyelin(d18:1/22:0)	NA	NA	NA
Sphingomyelin(d18:1/22:1)	NA	NA	NA
Sphingomyelin(d18:1/23:0)	NA	NA	NA
Sphingomyelin(d18:1/23:1)	NA	NA	NA
Sphingomyelin(d18:1/24:0)	NA	NA	NA
Sphingomyelin(d18:1/24:1)	NA	NA	NA
Sphingomyelin(d18:1/24:2)	NA	NA	NA
Sphingomyelin(d18:1/25:0)	NA	NA	NA
Sphingomyelin(d18:1/25:1)	NA	NA	NA
Sphingosine 1-phosphate 18(0)	16893	91486	LMSP01050002
Sphingosine 1-phosphate 18(1)	37550	5283560	LMSP01050001
Sphingosine 1-phosphate 18(2)	NA	NA	NA
Stearoylcarnitine	73074	NA	NA
Symmetric dimethylarginine	25682	NA	NA
Taurine	15891	NA	NA
Taurochenodeoxycholic acid	16525	387316	LMST05040005
Taurocholic acid	28865	6675	LMST05040001
Taurodeoxycholic acid	9410	2733768	LMST05040013
Taurolithocholic acid	36259	439763	LMST05040003
Tetradecadienylcarnitine	86069	NA	NA
Tetradecenoylcarnitine	86066	NA	NA
TG(42:0)	NA	NA	NA
TG(42:1)	NA	NA	NA
TG(42:2)	NA	NA	NA

TG(44:0)	NA	NA	NA
TG(44:1)	NA	NA	NA
TG(44:2)	NA	NA	NA
TG(45:0)	NA	NA	NA
TG(46:0)	NA	NA	NA
TG(46:1)	NA	NA	NA
TG(46:2)	NA	NA	NA
TG(48:1)	NA	NA	NA
TG(48:2)	NA	NA	NA
TG(48:3)	NA	NA	NA
TG(50:1)	NA	NA	NA
TG(50:2)	NA	NA	NA
TG(50:3)	NA	NA	NA
TG(50:4)	NA	NA	NA
TG(51:1)	NA	NA	NA
TG(51:2)	NA	NA	NA
TG(51:3)	NA	NA	NA
TG(51:4)	NA	NA	NA
TG(52:1)	NA	NA	NA
TG(52:2)	NA	NA	NA
TG(52:3)	NA	NA	NA
TG(52:4)	NA	NA	NA
TG(53:1)	NA	NA	NA
TG(54:0)	NA	NA	NA
TG(54:1)	NA	NA	NA
TG(54:2)	NA	NA	NA
TG(54:3)	NA	NA	NA
TG(54:4)	NA	NA	NA
TG(54:5)	NA	NA	NA
TG(54:7)	NA	NA	NA
TG(55:1)	NA	NA	NA
TG(56:0)	NA	NA	NA
TG(56:1)	NA	NA	NA
TG(56:2)	NA	NA	NA
TG(56:3)	NA	NA	NA
TG(56:4)	NA	NA	NA
TG(56:5)	NA	NA	NA
TG(56:6)	NA	NA	NA
TG(56:7)	NA	NA	NA
TG(57:1)	NA	NA	NA
TG(58:2)	NA	NA	NA
TG(58:8)	NA	NA	NA
TG(58:9)	NA	NA	NA

TG(59:1)	NA	NA	NA
TG(60:1)	NA	NA	NA
TG(60:2)	NA	NA	NA
TG(60:3)	NA	NA	NA
Thromboxane B2	28728	5283137	LMFA03030002
Tiglylcarnitine	71179	NA	NA
Trimethylamine N-oxide	15724	NA	NA
Valerylcarnitine	86050	NA	NA

Metabolites are ordered alphabetically, NA: not avaliable

Table S2. Influence of the recruitment site on metabolite levels for HC and all IPD patients combined (n=204)

Name	Mean all	Mean UL	Mean UCL	Beta	SE	Nominal *p*	Adjusted *p*
Sphingosine 1-phosphate 18(1)	-0.045	-1.071	0.689	1.731	0.077	2.22e-56	6.74e-54
Sphingosine 1-phosphate 18(0)	-0.05	-1.077	0.683	1.752	0.079	1.33e-55	2.02e-53
12-HETE	-0.044	-1.079	0.696	1.728	0.08	7.17e-54	7.27e-52
12(S)-HEPE	-0.03	-1.06	0.706	1.758	0.082	2.88e-53	2.19e-51
(+/-) 14-HDoHE	-0.022	-1.049	0.711	1.759	0.083	5.59e-53	3.4e-51
(+/-) 10-HDoHE	-0.017	-1.017	0.697	1.735	0.087	4.06e-49	2.06e-47
O-Phosphoethanolamine	-0.039	-1.023	0.673	1.652	0.087	5.09e-46	2.21e-44
Taurine	-0.037	-0.989	0.652	1.605	0.092	7.85e-42	2.98e-40
(+/-) 11-HDoHE	-0.015	-0.989	0.681	Jan 68	0.098	5.84e-41	1.97e-39
Sphingosine 1-phosphate 18(2)	-0.051	-0.945	0.587	1.475	0.089	1.59e-39	4.82e-38
LPS 18(1)	-0.046	-0.977	0.619	1.548	0.097	1.36e-37	3.75e-36
8(9)-EpETrE	-0.045	-0.93	0.587	1.502	0.101	5.52e-34	1.4e-32
Serotonine	-0.025	-0.893	0.603	1.456	0.107	4.16e-30	9.72e-29
8-HETE	-0.079	-0.865	0.483	1.267	0.107	7.83e-25	1.7e-23
Isocitrate	-0.042	-0.696	0.424	1.268	0.116	5.9e-22	1.2e-20
15(S)-HETrE	-0.068	-0.793	0.45	1.193	0.118	1.06e-19	2.02e-18
13-HODE	-0.005	-0.71	0.498	1.143	0.123	2.19e-17	3.91e-16
Glutathione	-0.058	-0.675	0.389	0.949	0.124	8.36e-13	1.41e-11
Linoleylcarnitine	0.012	-0.534	0.401	0.911	0.125	8.23e-12	1.32e-10
FA 18(2)	0.031	-0.487	0.402	0.893	0.132	1.38e-10	2.09e-09
Putrescine	-0.005	-0.365	0.256	0.718	0.11	6.49e-10	9.39e-09
9,10-DiHOME	0.007	-0.523	0.386	0.845	0.138	4.79e-09	6.61e-08
11-HETE	-0.051	-0.535	0.295	0.779	0.135	3.11e-08	4.11e-07
Prostaglandin E2	0.027	-0.468	0.38	0.765	0.133	3.4e-08	4.31e-07
Thromboxane B2	0.008	-0.496	0.368	0.736	0.13	5.21e-08	6.33e-07
TG(54:2)	-0.002	0.45	-0.326	-0.791	0.145	1.61e-07	1.88e-06
Lysophosphatidylcholine(O-18:1)	-0.016	-0.514	0.34	0.773	0.142	1.74e-07	1.96e-06
Malic acid	-0.034	-0.412	0.236	0.71	0.134	3.36e-07	3.64e-06
9,10,13-TriHOME	0.016	0.462	-0.302	-0.718	0.141	8.24e-07	8.64e-06
9-HODE	0.014	-0.422	0.325	0.686	0.137	1.16e-06	1.17e-05
Sarcosine	-0.021	0.356	-0.293	-0.669	0.134	1.39e-06	1.36e-05
Prostaglandin F2α	-0.044	-0.45	0.246	0.654	0.134	2.24e-06	2.12e-05
9,12,13-TriHOME	0.003	0.392	-0.275	-0.659	0.141	5.1e-06	4.7e-05
TG(52:1)	-0.005	0.396	-0.293	-0.677	0.146	6.54e-06	5.85e-05
FA 22(6)	0.023	-0.308	0.26	0.659	0.143	7.68e-06	6.67e-05
TG(56:3)	-0.003	0.393	-0.286	-0.695	0.151	8.11e-06	6.84e-05
TG(51:1)	-0.016	0.41	-0.32	-0.665	0.145	8.45e-06	6.94e-05
TG(52:2)	-0.023	0.359	-0.296	-0.656	0.147	1.39e-05	0.000111
Phosphatidylcholine(O-38:4)	0.03	0.409	-0.241	-0.655	0.148	1.72e-05	0.000134
TG(51:2)	-0.014	0.404	-0.313	-0.64	0.145	1.78e-05	0.000135

O-Acetyl-L-serine	-0.007	-0.208	0.14	0.603	0.138	2.03e-05	0.000151
Cortisol	0.018	0.341	-0.213	-0.608	0.146	4.62e-05	0.000334
TG(50:2)	-0.042	0.312	-0.296	-0.588	0.144	6.68e-05	0.000468
Glycoursodeoxycholic acid	-0.054	0.226	-0.254	-0.535	0.131	6.78e-05	0.000468
TG(50:1)	-0.026	0.323	-0.276	-0.591	0.145	6.92e-05	0.000468
TG(54:3)	-0.005	0.325	-0.241	-0.611	0.152	8.83e-05	0.000584
TG(48:1)	-0.032	0.319	-0.283	-0.56	0.143	0.000122	0.00079
Phosphatidylcholine(36:1)	0.018	0.334	-0.208	-0.542	0.138	0.000129	0.000815
L-Glutamic acid	-0.015	-0.294	0.187	0.563	0.145	0.000136	0.000841
S-Methylcysteine	0.026	-0.29	0.254	0.585	0.151	0.000141	0.00086
Lysophosphatidylethanolamine (16:1)	-0.099	0.221	-0.328	-0.523	0.138	0.000208	0.00124
TG(53:1)	-0.002	0.346	-0.25	-0.572	0.152	0.000231	0.00135
3-Hydroxypropionic Acid	-0.061	-0.311	0.118	0.549	0.147	0.000244	0.0014
Lysophosphatidylinositol 18(1)	0.016	0.333	-0.21	-0.55	0.15	0.000328	0.00184
TG(56:4)	-0.012	0.296	-0.232	-0.546	0.153	0.000474	0.00262
Lysophosphatidic acid 16(1)	-0.138	0.124	-0.325	-0.438	0.125	0.000541	0.00294
TG(58:9)	0.022	-0.236	0.207	0.519	0.149	0.000622	0.00332
CE(22:6)	0.057	-0.173	0.222	0.49	0.141	0.000645	0.00337
TG(56:2)	-0.002	0.297	-0.216	-0.542	0.156	0.000654	0.00337
Methylmalonylcarnitine	0.033	-0.246	0.233	0.468	0.137	0.000781	0.00396
Lysophosphatidylinositol 16(1)	-0.092	0.14	-0.258	-0.49	0.144	0.0008	0.00399
TG(54:1)	-0.003	0.32	-0.235	-0.521	0.153	0.000815	0.004
TG(48:2)	-0.039	0.26	-0.253	-0.486	0.143	0.000833	0.00402
Phosphatidylethanolamine(34:2)	-0.021	0.259	-0.222	-0.488	0.148	0.0012	0.00572
TG(46:1)	-0.034	0.243	-0.232	-0.459	0.142	0.00141	0.00659
Lysophosphatidic acid 18(1)	-0.046	0.175	-0.204	-0.434	0.135	0.0015	0.00693
14,15-DiHETE	0.017	-0.24	0.2	0.482	0.15	0.00156	0.00706
Lysophosphatidylcholine(20:5)	0.052	-0.155	0.199	0.408	0.127	0.00161	0.00718
Lysophosphatidylcholine(22:6)	0.03	-0.241	0.223	0.475	0.149	0.00174	0.00767
Ornithine	-0.064	-0.219	0.048	0.427	0.135	0.00187	0.00811
19,20-DiHDPA	0.049	-0.176	0.21	0.465	0.148	0.00191	0.00817
1-LG/2-LG	-0.027	0.219	-0.204	-0.442	0.142	0.00208	0.00877
Phosphatidylcholine(38:3)	-0.075	0.181	-0.259	-0.45	0.147	0.00263	0.011
Phosphatidylcholine(32:1)	-0.079	0.196	-0.275	-0.423	0.14	0.00288	0.0118
Phosphatidylethanolamine(36:4)	-0.012	0.246	-0.196	-0.415	0.143	0.00406	0.0163
Lysophosphatidic acid 14(0)	-0.091	0.199	-0.297	-0.366	0.126	0.00408	0.0163
(+/-) 8-HDoHE	-0.017	-0.213	0.123	0.445	0.156	0.00468	0.0185
Lysophosphatidic acid 16(0)	-0.082	0.139	-0.24	-0.362	0.129	0.00548	0.0214
Taurodeoxycholic acid	0.008	0.219	-0.142	-0.392	0.142	0.00624	0.024
Phosphatidylcholine(38:6)	0.031	-0.157	0.166	0.397	0.144	0.00648	0.0246
Taurochenodeoxycholic acid	0.013	0.223	-0.138	-0.397	0.145	0.00665	0.0247
2-Hydroxybutyric acid	-0.006	-0.235	0.157	0.413	0.151	0.00666	0.0247
Phosphatidylcholine(36:5)	0.004	-0.152	0.116	0.393	0.145	0.00722	0.0264

Phosphatidylcholine(40:7)	0.043	-0.162	0.19	0.383	0.142	0.00761	0.0272
Phosphatidylcholine(O-36:4)	0.021	0.214	-0.116	-0.422	0.157	0.00764	0.0272
L-Tyrosine	-0.045	0.224	-0.24	-0.375	0.139	0.00769	0.0272
Lauroylcarnitine	0.042	0.291	-0.135	-0.392	0.149	0.00924	0.0323
Phosphatidylcholine(34:1)	0.053	0.112	0.01	-0.1	0.038	0.00951	0.0325
Lysophosphatidic acid 18(2)	-0.032	0.125	-0.144	-0.368	0.141	0.00957	0.0325
L-Arginine	-0.038	0.2	-0.211	-0.383	0.147	0.00963	0.0325
TG(51:3)	-0.008	0.243	-0.187	-0.386	0.15	0.0111	0.0371
TG(50:3)	-0.041	0.192	-0.208	-0.383	0.15	0.0116	0.0382
FA 22(5) w6	-0.041	-0.267	0.121	0.361	0.143	0.0124	0.0405
L-Asparagine	-0.028	-0.276	0.152	0.352	0.141	0.0134	0.0431
Lysophosphatidylethanolamine (20:5)	0.011	-0.113	0.099	0.363	0.146	0.0135	0.0431
Methyldopa	-0.042	-0.098	-0.002	0.311	0.125	0.0136	0.0431
Lysophosphatidylethanolamine (20:4)	-0.034	0.197	-0.199	-0.361	0.146	0.0139	0.0435
Phosphatidylcholine(40:8)	0.067	-0.125	0.204	0.31	0.126	0.0148	0.0458
9-HOTrE	0.004	-0.235	0.175	0.362	0.149	0.0159	0.0488
CE(18:1)	0.018	0.207	-0.117	-0.37	0.153	0.0164	0.05
Lysophosphatidylinositol 20(4)	-0.042	0.147	-0.178	-0.344	0.144	0.0178	0.0536
TG(56:5)	-0.021	0.185	-0.169	-0.369	0.158	0.0202	0.0599
L-Tryptophan	-0.028	0.071	-0.1	-0.333	0.142	0.0203	0.0599
Phosphatidylcholine(38:2)	-0.043	0.147	-0.179	-0.347	0.149	0.0208	0.0609
Myristoilcarnitine	0.024	0.316	-0.185	-0.343	0.148	0.0212	0.0612
Glycochenodeoxycholic acid	-0.022	0.122	-0.125	-0.314	0.135	0.0213	0.0612
Sphingomyelin(d18:1/18:0)	-0.026	0.15	-0.153	-0.329	0.142	0.0216	0.0615
1-AG/2-AG	-0.025	0.191	-0.179	-0.331	0.144	0.0225	0.0633
TG(48:3)	-0.038	0.15	-0.173	-0.329	0.146	0.0252	0.0703
17,18-DiHETE	0.036	-0.088	0.126	0.327	0.147	0.027	0.0747
DL-3-aminoisobutyric acid	-0.021	0.197	-0.178	-0.323	0.145	0.0274	0.0752
Phosphatidylcholine(36:3)	-0.021	0.139	-0.136	-0.328	0.148	0.028	0.0761
L-Kynurenine	-0.057	0.176	-0.226	-0.331	0.15	0.0285	0.0762
TG(52:3)	-0.019	0.159	-0.147	-0.343	0.155	0.0286	0.0762
TG(46:2)	-0.039	0.139	-0.167	-0.309	0.142	0.0301	0.0797
Cer(d18:1/23:0)	0.02	0.216	-0.12	-0.319	0.146	0.0308	0.0807
Linoleoyl ethanolamide	-0.017	-0.224	0.131	0.311	0.146	0.0348	0.0905
5-HETE	-0.098	-0.28	0.031	0.3	0.143	0.0367	0.0945
TG(44:1)	-0.019	0.149	-0.139	-0.289	0.142	0.0436	0.111
DGLEA	-0.077	0.108	-0.21	-0.296	0.146	0.044	0.112
Lysophosphatidylcholine(16:1)	-0.065	0.08	-0.168	-0.301	0.149	0.0449	0.113
1-Methylhistidine	0.04	0.244	-0.108	-0.288	0.143	0.0452	0.113
Betaine	0.052	-0.11	0.167	0.287	0.144	0.0478	0.118
TG(54:7)	0.025	-0.144	0.146	0.292	0.154	0.0587	0.144
Citric acid	-0.025	-0.032	-0.02	0.246	0.13	0.0607	0.147
Lysophosphatidylcholine(20:3)	-0.045	0.063	-0.123	-0.28	0.149	0.061	0.147

Lysophosphatidylethanolamine(22:6)	0.016	-0.088	0.09	0.27	0.143	0.0618	0.147
Gamma-aminobutyric acid	-0.059	-0.24	0.071	0.264	0.141	0.0623	0.147
Cer(d18:1/22:0)	-0.015	0.145	-0.129	-0.302	0.161	0.0625	0.147
Choline	-0.023	-0.086	0.022	0.264	0.143	0.0671	0.156
(+/-) 16-HDoHE	0.018	-0.055	0.071	0.272	0.148	0.0674	0.156
TG(56:6)	-0.007	0.147	-0.117	-0.29	0.158	0.0682	0.156
Phosphatidylcholine(40:6)	0.028	-0.068	0.097	0.265	0.145	0.0684	0.156
Lactic acid	-0.033	-0.149	0.05	0.276	0.151	0.0695	0.158
Phosphatidylcholine(36:2)	0.017	0.126	-0.061	-0.273	0.153	0.0766	0.173
Anandamide	-0.11	0.01	-0.196	-0.265	0.15	0.0785	0.175
Carnitine	-0.003	0.188	-0.139	-0.246	0.14	0.0798	0.177
L-4-hydroxy-proline	-0.061	0.101	-0.179	-0.251	0.143	0.0809	0.178
TG(58:8)	0.016	-0.08	0.085	0.267	0.154	0.0842	0.184
O-Anandamide	-0.088	0.027	-0.171	-0.26	0.151	0.0872	0.189
Sphingomyelin(d18:1/15:0)	0.008	0.234	-0.154	-0.247	0.144	0.0881	0.189
Lysophosphatidylcholine(18:1)	0.063	0.161	-0.008	-0.259	0.151	0.0882	0.189
Lysophosphatidylethanolamine(18:0)	0.029	0.223	-0.11	-0.251	0.147	0.0889	0.189
Decanoylcarnitine	0.047	0.23	-0.083	-0.251	0.148	0.0917	0.193
L-Isoleucine	0.005	0.129	-0.085	-0.239	0.141	0.0922	0.193
Cer(d18:1/24:1)	-0.028	0.118	-0.133	-0.263	0.157	0.0948	0.197
Ethanolamine	-0.071	-0.03	-0.101	-0.228	0.137	0.0971	0.201
Cysteine	-0.036	0.229	-0.228	-0.218	0.133	0.101	0.208
Lysophosphatidylinositol 18(0)	-0.06	0.174	-0.228	-0.22	0.134	0.102	0.208
Glycodeoxycholic acid	-0.029	0.09	-0.115	-0.248	0.152	0.104	0.209
TG(55:1)	-0.018	0.126	-0.121	-0.267	0.164	0.104	0.209
Decenoylcarnitine	0.035	-0.098	0.13	0.245	0.151	0.105	0.209
Sphingomyelin(d18:1/23:0)	0.006	0.12	-0.076	-0.248	0.152	0.105	0.209
Propionylcarnitine	0.001	0.197	-0.139	-0.218	0.134	0.106	0.209
TG(46:0)	-0.015	0.124	-0.114	-0.241	0.148	0.107	0.209
5,6-DiHETrE	0.033	0.137	-0.042	-0.233	0.145	0.11	0.215
Tetradecenoylcarnitine	0.022	0.162	-0.077	-0.239	0.15	0.111	0.216
TG(54:4)	0.002	0.103	-0.07	-0.246	0.157	0.12	0.231
Phosphatidylethanolamine(38:2)	0.039	0.21	-0.084	-0.222	0.144	0.125	0.238
12,13-DiHOME	0.022	-0.153	0.148	0.227	0.147	0.125	0.238
8,9-DiHETrE	-0.008	0.075	-0.067	-0.215	0.142	0.132	0.25
Tiglylcarnitine	0.046	-0.026	0.097	0.221	0.147	0.136	0.255
TG(50:4)	-0.023	0.101	-0.113	-0.225	0.15	0.136	0.255
Cer(d18:0/23:0)	0.012	0.143	-0.081	-0.223	0.15	0.139	0.258
TG(56:1)	-0.002	0.119	-0.089	-0.242	0.163	0.141	0.26
Stearoylcarnitine	0.06	0.253	-0.079	-0.205	0.141	0.147	0.269
Deoxycholic acid	-0.034	0.086	-0.12	-0.219	0.151	0.148	0.27
Cer(d18:0/22:0)	-0.008	0.1	-0.085	-0.216	0.154	0.161	0.291
TG(60:1)	-0.014	0.07	-0.074	-0.217	0.155	0.162	0.291

Lysophosphatidylethanolamine(20:4)	0.003	0.108	-0.073	-0.213	0.152	0.164	0.294
TG(60:2)	-0.016	0.087	-0.09	-0.217	0.156	0.166	0.294
Phosphatidylcholine(O-34:2)	0.015	0.145	-0.078	-0.231	0.166	0.166	0.294
L-Glutamine	0.019	0.143	-0.071	-0.206	0.149	0.168	0.295
Sphingomyelin(d18:1/14:0)	-0.002	0.18	-0.133	-0.203	0.15	0.179	0.313
Hexanoylcarnitine	0.034	0.209	-0.091	-0.202	0.15	0.181	0.314
Phosphatidylcholine(34:3)	-0.047	0.059	-0.123	-0.199	0.149	0.183	0.315
TG(44:0)	-0.02	0.086	-0.096	-0.198	0.148	0.183	0.315
Dodecenoylcarnitine	0.001	0.126	-0.089	-0.193	0.146	0.186	0.317
L-Homoserine	0.009	-0.078	0.071	0.207	0.157	0.188	0.32
L-2-aminoadipic acid	-0.007	-0.061	0.032	0.179	0.137	0.192	0.323
Deoxycarnitine	0.038	-0.04	0.094	0.156	0.119	0.192	0.323
Phosphatidylcholine(O-34:1)	0.028	0.137	-0.049	-0.187	0.145	0.199	0.332
TG(42:0)	-0.014	0.081	-0.081	-0.194	0.152	0.202	0.335
L-Lysine	-0.017	-0.117	0.055	0.192	0.152	0.209	0.342
Phosphatidylethanolamine(38:4)	0.03	0.192	-0.086	-0.181	0.144	0.209	0.342
TG(58:2)	0.002	0.08	-0.054	-0.201	0.16	0.211	0.342
Valerylcarnitine	-0.001	0.173	-0.124	-0.178	0.142	0.212	0.342
Phosphatidylcholine(38:5)	0.02	-0.083	0.094	0.189	0.151	0.212	0.342
TG(51:4)	-0.013	0.126	-0.112	-0.188	0.151	0.214	0.344
Sphingomyelin(d18:1/21:0)	-0.007	0.121	-0.098	-0.187	0.15	0.215	0.344
FA 18(1)	-0.03	-0.093	0.015	0.17	0.146	0.244	0.388
Sphingomyelin(d18:1/18:2)	-0.017	-0.133	0.066	0.205	0.176	0.246	0.389
TG(59:1)	-0.061	0.01	-0.111	-0.188	0.162	0.248	0.389
Lysophosphatidylcholine(O-16:0)	0	-0.149	0.107	0.178	0.154	0.249	0.389
TG(60:3)	0.019	0.112	-0.047	-0.184	0.159	0.25	0.389
Lysophosphatidylcholine(14:0)	-0.023	0.109	-0.118	-0.176	0.155	0.257	0.398
TG(42:1)	-0.024	0.043	-0.072	-0.158	0.144	0.275	0.425
3-Hydroxybutyric acid	0.028	-0.051	0.085	0.166	0.155	0.284	0.436
DG(36:2)	-0.024	0.049	-0.077	-0.167	0.156	0.285	0.436
Oleoylcarnitine	-0.015	-0.07	0.025	0.156	0.147	0.287	0.436
12,13-DiHODE	0.03	-0.079	0.107	0.153	0.143	0.288	0.436
Hydroxylysine	-0.085	0.069	-0.197	-0.158	0.152	0.299	0.449
L-Alpha-aminobutyric acid	0.068	-0.035	0.143	0.154	0.148	0.3	0.449
Sphingomyelin(d18:1/20:0)	-0.026	0.04	-0.073	-0.156	0.151	0.302	0.449
Tetradecadienylcarnitine	0.039	-0.082	0.125	0.152	0.147	0.303	0.449
TG(44:2)	-0.029	0.051	-0.087	-0.144	0.143	0.315	0.464
Octanoylcarnitine	0.045	0.175	-0.048	-0.15	0.151	0.321	0.47
Butyrylcarnitine	-0.028	0.06	-0.091	-0.147	0.147	0.322	0.47
Lysophosphatidylcholine(16:0)	0.015	0.044	-0.007	-0.15	0.152	0.325	0.472
Isovalerylcarnitine	0.064	0.121	0.024	-0.132	0.135	0.327	0.472
Lysophosphatidylcholine(O-16:1)	0.016	-0.119	0.112	0.151	0.154	0.33	0.472
Homocysteine	-0.015	0.153	-0.137	-0.135	0.139	0.331	0.472

Phosphatidylcholine(34:2)	-0.005	0.042	-0.038	-0.148	0.152	0.331	0.472
Phosphatidylcholine(O-38:6)	0.047	-0.025	0.099	0.148	0.154	0.34	0.483
Sphingomyelin(d18:1/25:0)	0.02	0.088	-0.029	-0.149	0.157	0.345	0.488
TG(56:7)	0.009	-0.055	0.055	0.147	0.158	0.353	0.497
Pimelylcarnitine	0.023	-0.08	0.096	0.129	0.141	0.362	0.505
Sphingomyelin(d18:1/24:2)	-0.011	-0.128	0.072	0.139	0.152	0.364	0.505
Phosphatidylcholine(O-38:5)	0.028	0.072	-0.004	-0.146	0.16	0.364	0.505
Dehydroepiandrosterone	0.039	0.023	0.05	0.13	0.145	0.37	0.512
Taurocholic acid	0	0.064	-0.045	-0.129	0.144	0.373	0.513
L-Leucine	0.013	0.055	-0.018	-0.119	0.136	0.383	0.524
Lysophosphatidylcholine(18:0)	0.044	0.072	0.024	-0.135	0.156	0.387	0.528
Phosphatidylethanolamine(O-36:5)	0.034	-0.072	0.111	0.136	0.158	0.391	0.528
Phosphatidylethanolamine(O-38:7)	0.044	-0.006	0.079	0.129	0.151	0.391	0.528
Cystathionine	-0.006	0.186	-0.145	-0.104	0.121	0.393	0.528
Lysophosphatidylcholine(18:3)	-0.005	0.065	-0.055	-0.14	0.171	0.414	0.554
TG(54:5)	-0.007	0.022	-0.028	-0.127	0.156	0.416	0.554
Pyroglutamic acid	-0.032	-0.087	0.008	0.123	0.153	0.424	0.563
Hexadecenoylcarnitine	-0.016	0.121	-0.114	-0.116	0.148	0.435	0.572
Lysophosphatidylethanolamine (16:0)	0.015	-0.017	0.038	0.116	0.148	0.435	0.572
Sphingomyelin(d18:1/22:0)	-0.004	-0.009	-0.001	-0.117	0.153	0.444	0.582
Sphingomyelin(d18:1/25:1)	0.014	0.105	-0.05	-0.11	0.147	0.454	0.592
Sphingomyelin(d18:1/18:1)	-0.025	0.019	-0.056	-0.1	0.138	0.473	0.614
Sphingomyelin(d18:1/20:1)	-0.044	-0.151	0.033	0.103	0.148	0.487	0.627
14,15-DiHETrE	-0.033	-0.032	-0.034	-0.102	0.146	0.487	0.627
L-Histidine	-0.012	-0.037	0.006	-0.104	0.152	0.492	0.631
Isobutyrylcarnitine	0.072	0.106	0.047	0.094	0.144	0.514	0.657
Palmitoyl ethanolamide	-0.04	0.046	-0.101	-0.097	0.152	0.522	0.664
Lysophosphatidylcholine(18:2)	0.064	0.032	0.087	-0.096	0.15	0.525	0.664
Phosphatidylcholine(O-36:5)	0.024	0.054	0.003	-0.096	0.155	0.537	0.678
Nonaylcarnitine	0.038	0.173	-0.059	-0.088	0.144	0.545	0.682
L-Alanine	-0.053	-0.086	-0.029	0.095	0.157	0.546	0.682
Citrulline	-0.012	0.096	-0.091	-0.09	0.149	0.549	0.682
Symmetric dimethylarginine	0.037	0.158	-0.05	-0.086	0.144	0.549	0.682
L-Methionine	-0.053	-0.15	0.017	0.077	0.131	0.557	0.687
Trimethylamine N-oxide	-0.014	0.048	-0.058	0.088	0.15	0.559	0.687
L-Valine	0.045	0.085	0.016	-0.084	0.145	0.56	0.687
CE(18:2)	0.03	-0.046	0.084	0.087	0.15	0.564	0.689
TG(52:4)	-0.013	0.003	-0.025	-0.087	0.157	0.581	0.703
Phosphatidylcholine(O-36:3)	0.048	0.102	0.009	-0.08	0.145	0.581	0.703
Lysophosphatidylcholine(20:4)	0.031	-0.079	0.109	0.082	0.149	0.582	0.703
Acetylcarnitine	0.004	0.073	-0.046	-0.08	0.146	0.586	0.703
L-Threonine	0.015	-0.1	0.098	0.079	0.145	0.587	0.703
Methionine sulfone	0.028	0.053	0.011	0.077	0.148	0.604	0.72

TG(57:1)	-0.002	-0.068	0.045	0.076	0.151	0.615	0.73
Phosphatidylcholine(32:2)	-0.042	0.014	-0.082	-0.074	0.151	0.626	0.74
Phosphatidylcholine(O-44:5)	0.047	-0.001	0.081	0.075	0.155	0.63	0.742
2-Ketoglutaric acid	-0.073	-0.104	-0.051	0.067	0.141	0.634	0.744
Phosphatidylcholine(40:5)	-0.036	0.009	-0.068	-0.073	0.154	0.637	0.744
3-Methoxytyrosine	-0.049	0.076	-0.14	0.048	0.104	0.646	0.752
Glycylglycine	-0.021	-0.059	0.006	0.062	0.146	0.673	0.781
TG(56:0)	0.022	-0.032	0.06	0.065	0.159	0.683	0.789
Phosphatidylcholine(O-34:3)	0.044	-0.027	0.096	0.058	0.149	0.696	0.802
Saccharopine	-0.046	-0.109	-0.001	0.055	0.145	0.703	0.807
Sphingomyelin(d18:1/24:1)	-0.005	-0.034	0.016	-0.057	0.156	0.715	0.816
Sphingomyelin(d18:1/22:1)	-0.004	-0.006	-0.003	-0.055	0.151	0.716	0.816
Phosphatidylcholine(38:7)	-0.005	-0.07	0.041	0.052	0.155	0.736	0.833
Lysophosphatidylethanolamine(18:1)	-0.027	0.017	-0.058	-0.049	0.146	0.737	0.833
Octenoylcarnitine	-0.012	-0.042	0.01	-0.047	0.15	0.753	0.845
2-Methylbutyroylcarnitine	0.043	0.133	-0.021	-0.042	0.135	0.755	0.845
Malonylcarnitine	-0.007	-0.024	0.006	0.047	0.152	0.756	0.845
Gamma-Glutamylglutamine	-0.057	-0.118	-0.013	0.044	0.143	0.759	0.845
L-Serine	0.001	-0.059	0.045	-0.044	0.147	0.763	0.846
Phosphatidylcholine(38:4)	0.007	-0.044	0.044	0.042	0.158	0.79	0.869
Phosphatidylcholine(36:4)	0	-0.055	0.04	0.041	0.155	0.791	0.869
Phosphatidylcholine(34:4)	-0.039	-0.002	-0.066	-0.039	0.146	0.792	0.869
TG(45:0)	-0.016	-0.004	-0.024	-0.039	0.155	0.799	0.871
Sphingomyelin(d18:1/16:0)	0.021	0.004	0.033	-0.04	0.16	0.801	0.871
11,12-DiHETrE	-0.03	-0.078	0.004	-0.036	0.145	0.802	0.871
Glycocholic acid	-0.028	-0.035	-0.024	-0.034	0.137	0.807	0.873
Palmitoylcarnitine	-0.01	0.082	-0.077	-0.034	0.148	0.819	0.882
Sphingomyelin(d18:1/16:1)	-0.004	0.001	-0.007	-0.035	0.152	0.821	0.882
12,13-EpOME	0.013	-0.015	0.034	0.033	0.162	0.841	0.898
Sphingomyelin(d18:1/23:1)	0.003	0.035	-0.019	-0.028	0.143	0.846	0.898
TG(42:2)	-0.029	-0.036	-0.025	-0.028	0.146	0.849	0.898
CE(20:4)	0.014	-0.01	0.032	0.03	0.159	0.85	0.898
L-Phenylalanine	-0.047	-0.045	-0.048	-0.026	0.141	0.851	0.898
Homocitrulline	0.017	0.09	-0.036	0.026	0.15	0.862	0.904
8,12-iPF2a IV	-0.102	-0.166	-0.057	0.023	0.131	0.863	0.904
DG(36:3)	-0.012	-0.025	-0.003	-0.023	0.153	0.879	0.915
Glycolithocholic acid	-0.047	-0.077	-0.026	0.023	0.15	0.879	0.915
Lysophosphatidylinositol 18(2)	-0.024	-0.112	0.039	0.02	0.148	0.891	0.924
TG(54:0)	0.021	0.031	0.013	-0.02	0.161	0.9	0.928
Cholic acid	-0.043	-0.02	-0.059	0.018	0.143	0.903	0.928
Gamma-L-glutamyl-L-alanine	-0.04	-0.019	-0.055	-0.018	0.149	0.903	0.928
N6,N6,N6-Trimethyl-L-lysine	-0.019	0.016	-0.044	-0.016	0.138	0.908	0.929
Sphingomyelin(d18:1/24:0)	0.002	-0.074	0.056	0.017	0.154	0.911	0.929

Phosphatidylethanolamine(O-38:5)	0.033	0.009	0.051	0.014	0.16	0.93	0.943
Phosphatidylethanolamine(38:6)	0.036	0.08	0.005	-0.012	0.138	0.931	0.943
Taurolithocholic acid	-0.012	-0.024	-0.004	-0.01	0.148	0.946	0.956
Phosphatidylcholine(32:0)	0.012	0.048	-0.013	-0.007	0.15	0.962	0.968
L-Proline	-0.048	-0.044	-0.051	0.002	0.143	0.989	0.993
Glycine	-0.028	-0.04	-0.019	-0.001	0.141	0.995	0.995

Shown are the results of a linear regression for each metabolite and site separately: regression coefficient (beta), standard error (SE), nominal and adjusted p-values.

Table S3. Influence of age at examination on metabolites levels for HC and all IPD patients combined (n=204)

Name	z-value	Nominal *p*	Adjusted *p*	Beta (CI)
O-Acetyl-L-serine	5.994	2.05e-09	6.24e-07	0.032 (0.021;0.042)
Cystathionine	4.981	6.31e-07	9.59e-05	0.023 (0.014;0.032)
Cysteine	4.769	1.85e-06	0.000187	0.024 (0.014;0.034)
3-Methoxytyrosine	4.278	1.88e-05	0.00143	0.017 (0.009;0.024)
Citric acid	4.207	2.59e-05	0.00155	0.021 (0.011;0.03)
Sphingomyelin(d18:1/15:0)	4.169	3.07e-05	0.00155	0.023 (0.012;0.033)
Sphingomyelin(d18:1/22:0)	-3.865	0.000111	0.00482	-0.022 (-0.033;-0.011)
Isobutyrylcarnitine	3.743	0.000182	0.00614	0.02 (0.01;0.031)
Lysophosphatidylinositol 18(0)	3.763	0.000168	0.00614	0.019 (0.009;0.029)
L-Tryptophan	-3.664	0.000248	0.00717	-0.02 (-0.03;-0.009)
Trimethylamine N-oxide	3.653	0.000259	0.00717	0.021 (0.01;0.032)
Lysophosphatidylethanolamine (20:5)	3.625	0.000289	0.00732	0.02 (0.009;0.031)
Lysophosphatidic acid 14(0)	3.581	0.000342	0.00799	0.017 (0.008;0.027)
Sphingomyelin(d18:1/24:0)	-3.562	0.000368	0.00799	-0.02 (-0.032;-0.009)
Nonaylcarnitine	3.524	0.000425	0.00819	0.019 (0.009;0.03)
Isocitrate	Mär 52	0.000431	0.00819	0.015 (0.007;0.024)
Homocitrulline	3.458	0.000544	0.00918	0.02 (0.009;0.031)
Lysophosphatidylcholine(18:2)	-3.47	0.000521	0.00918	-0.02 (-0.031;-0.008)
Ethanolamine	-3.312	0.000925	0.0148	-0.017 (-0.027;-0.007)
Choline	3.283	0.00103	0.0156	0.018 (0.007;0.029)
(+/-) 16-HDoHE	3.195	0.0014	0.0202	0.018 (0.007;0.028)
Phosphatidylcholine(36:5)	3.175	0.0015	0.0207	0.017 (0.007;0.028)
Myristoilcarnitine	3.152	0.00162	0.0215	0.017 (0.006;0.027)
Lysophosphatidylinositol 18(2)	-3.103	0.00191	0.0242	-0.017 (-0.029;-0.006)
L-Threonine	-3.075	0.00211	0.0256	-0.017 (-0.028;-0.006)
CE(22:6)	2.993	0.00276	0.0323	0.016 (0.005;0.026)
Methionine sulfone	2.974	0.00294	0.0331	0.017 (0.006;0.028)
Lysophosphatidylcholine(20:3)	-2.925	0.00344	0.0374	-0.016 (-0.027;-0.005)
8-HETE	-2.885	0.00392	0.0411	-0.012 (-0.02;-0.004)
Lysophosphatidylcholine(20:4)	-2.827	0.0047	0.0476	-0.016 (-0.027;-0.005)
Symmetric dimethylarginine	2.789	0.00529	0.0519	0.015 (0.005;0.026)
L-Histidine	-2.71	0.00674	0.0622	-0.016 (-0.027;-0.004)
Tiglylcarnitine	2.709	0.00675	0.0622	0.015 (0.004;0.026)
L-Serine	-2.698	0.00698	0.0624	-0.015 (-0.026;-0.004)
Glutathione	-2.652	0.00801	0.0696	-0.012 (-0.021;-0.003)
Homocysteine	2.629	0.00856	0.0723	0.014 (0.004;0.024)
Methyldopa	Feb 59	0.00959	0.0782	0.012 (0.003;0.021)
Lysophosphatidylinositol 16(1)	-2.566	0.0103	0.0782	-0.014 (-0.025;-0.003)
Dehydroepiandrosterone	2.583	0.00979	0.0782	0.014 (0.003;0.025)
Phosphatidylethanolamine(38:2)	Feb 57	0.0102	0.0782	0.014 (0.003;0.025)

Sphingosine 1-phosphate 18(2)	-2.521	0.0117	0.085	-0.008 (-0.015;-0.002)
14,15-DiHETrE	-2.52	0.0117	0.085	-0.014 (-0.025;-0.003)
Sphingomyelin(d18:1/14:0)	2.506	0.0122	0.0864	0.014 (0.003;0.025)
Ornithine	2.481	0.0131	0.0904	0.013 (0.003;0.023)
11,12-DiHETrE	-2.469	0.0135	0.0914	-0.014 (-0.024;-0.003)
Phosphatidylethanolamine(38:4)	2.459	0.0139	0.092	0.013 (0.003;0.023)
Propionylcarnitine	2.397	0.0165	0.102	0.012 (0.002;0.022)
Stearoylcarnitine	2.401	0.0164	0.102	0.012 (0.002;0.022)
Sphingomyelin(d18:1/20:1)	-2.405	0.0162	0.102	-0.014 (-0.025;-0.002)
Phosphatidylcholine(40:6)	2.387	0.017	0.103	0.013 (0.002;0.024)
Lysophosphatidylethanolamine(18:0)	2.379	0.0174	0.103	0.013 (0.002;0.024)
Lysophosphatidylethanolamine(22:6)	2.363	0.0181	0.106	0.013 (0.002;0.023)
Valerylcarnitine	2.345	0.019	0.107	0.013 (0.002;0.023)
Lysophosphatidylcholine(18:1)	-2.33	0.0198	0.107	-0.013 (-0.024;-0.002)
Sphingomyelin(d18:1/24:1)	-2.331	0.0197	0.107	-0.014 (-0.025;-0.002)
TG(59:1)	-2.34	0.0193	0.107	-0.014 (-0.026;-0.002)
17,18-DiHETE	2.295	0.0217	0.116	0.013 (0.002;0.024)
Palmitoylcarnitine	Feb 21	0.0271	0.141	0.012 (0.001;0.022)
Thromboxane-B2	-2.206	0.0274	0.141	-0.011 (-0.02;-0.001)
2-Methylbutyroylcarnitine	2.179	0.0293	0.148	0.011 (0.001;0.021)
Citrulline	2.161	0.0307	0.151	0.012 (0.001;0.023)
Hexadecenoylcarnitine	2.164	0.0305	0.151	0.012 (0.001;0.022)
Phosphatidylcholine(38:6)	2.154	0.0313	0.151	0.012 (0.001;0.022)
L-Methionine	-2.146	0.0319	0.151	-0.011 (-0.02;-0.001)
Hydroxylysine	2.076	0.0379	0.176	0.012 (0.001;0.023)
13-HODE	-2.073	0.0382	0.176	-0.009 (-0.018;-0.001)
FA 22(6)	2.009	0.0446	0.197	0.011 (0;0.022)
12,13-DiHOME	-2.019	0.0435	0.197	-0.011 (-0.022;0)
Lysophosphatidylcholine(16:0)	-2.008	0.0447	0.197	-0.011 (-0.023;0)
8,9-DiHETrE	-1.985	0.0472	0.205	-0.011 (-0.021;0)
Sphingomyelin(d18:1/20:0)	-1.977	0.048	0.206	-0.011 (-0.022;0)
9,10-DiHOME	-1.953	0.0509	0.215	-0.01 (-0.02;0)
Phosphatidylethanolamine(38:6)	1.929	0.0537	0.224	0.01 (0;0.02)
12-HETE	-1.886	0.0593	0.244	-0.004 (-0.009;0)
Putrescine	1.868	0.0618	0.248	0.007 (0;0.015)
TG(54:5)	-1.867	0.0619	0.248	-0.011 (-0.022;0.001)
Hexanoylcarnitine	Jan 86	0.0629	0.248	0.011 (-0.001;0.022)
8,12-iPF2a IV	-1.848	0.0646	0.249	-0.009 (-0.019;0.001)
TG(54:4)	-1.847	0.0648	0.249	-0.011 (-0.022;0.001)
Octenoylcarnitine	-1.832	0.0669	0.254	-0.01 (-0.022;0.001)
19,20-DiHDPA	1.819	0.0688	0.258	0.01 (-0.001;0.021)
Phosphatidylcholine(40:7)	Jan 81	0.0703	0.261	0.01 (-0.001;0.02)
Octanoylcarnitine	1.794	0.0729	0.267	0.01 (-0.001;0.021)

TG(58:8)	1.784	0.0744	0.269	0.01 (-0.001;0.022)
(+/-) 8-HDoHE	1.764	0.0777	0.278	0.01 (-0.001;0.022)
L-Glutamic acid	1.758	0.0788	0.279	0.009 (-0.001;0.019)
Lysophosphatidylcholine(16:1)	-1.714	0.0866	0.302	-0.01 (-0.021;0.001)
O-Anandamide	-1.708	0.0876	0.303	-0.01 (-0.021;0.001)
Decanoylcarnitine	1.691	0.0908	0.307	0.009 (-0.001;0.02)
15(S)-HETrE	-1.692	0.0907	0.307	-0.007 (-0.016;0.001)
O-Phosphoethanolamine	-1.671	0.0947	0.31	-0.003 (-0.007;0.001)
Lysophosphatidylcholine(18:0)	-1.666	0.0957	0.31	-0.01 (-0.021;0.002)
Phosphatidylcholine(32:0)	1.671	0.0947	0.31	0.009 (-0.002;0.021)
TG(51:2)	1.667	0.0954	0.31	0.009 (-0.002;0.019)
Lysophosphatidylinositol 20(4)	-1.64	0.101	0.32	-0.009 (-0.02;0.002)
Sphingomyelin(d18:1/18:0)	-1.641	0.101	0.32	-0.009 (-0.019;0.002)
Sphingomyelin(d18:1/22:1)	-1.624	0.104	0.326	-0.009 (-0.02;0.002)
TG(58:9)	1.621	0.105	0.326	0.009 (-0.002;0.02)
TG(54:3)	-1.613	0.107	0.328	-0.009 (-0.02;0.002)
Gamma-Glutamylglutamine	-1.603	0.109	0.331	-0.009 (-0.019;0.002)
L-Kynurenine	1.582	0.114	0.335	0.009 (-0.002;0.02)
Carnitine	1.592	0.111	0.335	0.008 (-0.002;0.019)
Phosphatidylethanolamine(O-38:7)	1.584	0.113	0.335	0.009 (-0.002;0.02)
Anandamide	-1.572	0.116	0.336	-0.009 (-0.02;0.002)
Sphingomyelin(d18:1/25:1)	1.575	0.115	0.336	0.009 (-0.002;0.019)
L-2-aminoadipic acid	1.554	0.12	0.336	0.008 (-0.002;0.018)
L-Leucine	-1.552	0.121	0.336	-0.008 (-0.018;0.002)
Lysophosphatidylethanolamine (16:0)	1.557	0.119	0.336	0.009 (-0.002;0.02)
TG(51:1)	1.559	0.119	0.336	0.008 (-0.002;0.018)
1-Methylhistidine	Jan 53	0.126	0.348	0.008 (-0.002;0.019)
9-HOTrE	-1.495	0.135	0.369	-0.008 (-0.019;0.003)
Sphingomyelin(d18:1/23:1)	1.477	0.14	0.379	0.008 (-0.003;0.019)
Saccharopine	-1.469	0.142	0.382	-0.008 (-0.019;0.003)
S-Methylcysteine	1.453	0.146	0.386	0.008 (-0.003;0.02)
Cholic acid	1.453	0.146	0.386	0.008 (-0.003;0.019)
5,6-DiHETrE	-1.435	0.151	0.394	-0.008 (-0.018;0.003)
9-HODE	-1.43	0.153	0.394	-0.007 (-0.017;0.003)
Phosphatidylcholine(O-36:4)	-1.43	0.153	0.394	-0.008 (-0.02;0.003)
L-Asparagine	-1.402	0.161	0.411	-0.008 (-0.018;0.003)
L-Isoleucine	-1.372	0.17	0.425	-0.007 (-0.018;0.003)
L-Tyrosine	1.371	0.17	0.425	0.007 (-0.003;0.018)
Lysophosphatidylcholine(O-16:1)	-1.378	0.168	0.425	-0.008 (-0.019;0.003)
TG(60:1)	-1.356	0.175	0.433	-0.008 (-0.019;0.003)
TG(52:4)	-1.351	0.177	0.433	-0.008 (-0.019;0.004)
DL-3-aminoisobutyric acid	1.322	0.186	0.439	0.007 (-0.003;0.018)
Isovalerylcarnitine	-1.327	0.184	0.439	-0.007 (-0.017;0.003)

Lysophosphatidylcholine(O-16:0)	-1.326	0.185	0.439	-0.007 (-0.018;0.004)
Lysophosphatidylcholine(O-18:1)	-1.33	0.183	0.439	-0.007 (-0.017;0.003)
Phosphatidylcholine(36:2)	-1.33	0.184	0.439	-0.008 (-0.019;0.004)
Lysophosphatidic acid 18(2)	-1.299	0.194	0.446	-0.007 (-0.017;0.004)
Sphingomyelin(d18:1/18:1)	-1.308	0.191	0.446	-0.007 (-0.017;0.003)
TG(58:2)	-1.301	0.193	0.446	-0.008 (-0.019;0.004)
CE(18:1)	-1.283	0.2	0.453	-0.007 (-0.019;0.004)
Lysophosphatidylcholine(20:5)	1.286	0.198	0.453	0.006 (-0.003;0.015)
Lysophosphatidic acid 16(0)	1.258	0.208	0.469	0.006 (-0.003;0.015)
Prostaglandin E2	-1.249	0.212	0.473	-0.006 (-0.016;0.004)
Phosphatidylcholine(36:3)	-1.223	0.221	0.485	-0.007 (-0.018;0.004)
Phosphatidylcholine(38:7)	-1.231	0.218	0.485	-0.007 (-0.018;0.004)
TG(51:4)	1.222	0.222	0.485	0.007 (-0.004;0.017)
Taurine	-1.186	0.236	0.504	-0.003 (-0.009;0.002)
Sphingomyelin(d18:1/24:2)	-1.184	0.237	0.504	-0.007 (-0.018;0.004)
TG(52:3)	-1.187	0.235	0.504	-0.007 (-0.018;0.004)
TG(54:2)	-1.182	0.237	0.504	-0.006 (-0.017;0.004)
Glycine	-1.114	0.265	0.552	-0.006 (-0.016;0.004)
Sphingomyelin(d18:1/23:0)	-1.116	0.264	0.552	-0.006 (-0.017;0.005)
TG(56:3)	-1.119	0.263	0.552	-0.006 (-0.017;0.005)
TG(60:2)	-1.107	0.268	0.555	-0.006 (-0.018;0.005)
TG(56:6)	-1.1	0.271	0.557	-0.006 (-0.018;0.005)
Acetylcarnitine	01. Aug	0.28	0.568	0.006 (-0.005;0.017)
Malic acid	1.055	0.291	0.568	0.005 (-0.005;0.015)
Linoleoyl ethanolamide	-1.059	0.29	0.568	-0.006 (-0.016;0.005)
Lysophosphatidylcholine(18:3)	-1.058	0.29	0.568	-0.007 (-0.019;0.006)
Phosphatidylcholine(36:4)	-1.056	0.291	0.568	-0.006 (-0.017;0.005)
Sphingomyelin(d18:1/16:0)	-1.056	0.291	0.568	-0.006 (-0.018;0.005)
Sphingomyelin(d18:1/21:0)	1.056	0.291	0.568	0.006 (-0.005;0.017)
TG(56:5)	-1.079	0.281	0.568	-0.006 (-0.017;0.005)
Oleoylcarnitine	1.035	0.301	0.569	0.006 (-0.005;0.016)
Cortisol	-1.029	0.303	0.569	-0.006 (-0.016;0.005)
Lysophosphatidylethanolamine(20:4)	-1.034	0.301	0.569	-0.006 (-0.017;0.005)
Phosphatidylcholine(O-38:6)	1.034	0.301	0.569	0.006 (-0.005;0.018)
Phosphatidylcholine(O-44:5)	-1.044	0.297	0.569	-0.006 (-0.018;0.005)
TG(51:3)	1.032	0.302	0.569	0.005 (-0.005;0.016)
Lauroylcarnitine	1.015	0.31	0.575	0.006 (-0.005;0.016)
Tetradecadienylcarnitine	-1.016	0.31	0.575	-0.006 (-0.016;0.005)
Deoxycarnitine	0.999	0.318	0.585	0.004 (-0.004;0.012)
Phosphatidylcholine(38:4)	-0.986	0.324	0.594	-0.006 (-0.017;0.006)
Decenoylcarnitine	0.952	0.341	0.597	0.005 (-0.006;0.017)
3-Hydroxybutyric acid	0.941	0.347	0.597	0.006 (-0.006;0.017)
Lysophosphatidic acid 18(1)	-0.955	0.339	0.597	-0.005 (-0.015;0.005)

LPS 18(1)	-0.94	0.347	0.597	-0.003 (-0.009;0.003)
(+/-) 10-HDoHE	0.968	0.333	0.597	0.003 (-0.003;0.009)
Glycolithocholic acid	-0.958	0.338	0.597	-0.005 (-0.016;0.006)
Prostaglandin F2α	-0.941	0.347	0.597	-0.004 (-0.014;0.005)
Cer(d18:1/23:0)	0.956	0.339	0.597	0.005 (-0.006;0.016)
Phosphatidylcholine(34:2)	-0.94	0.347	0.597	-0.005 (-0.017;0.006)
Phosphatidylcholine(38:3)	-0.942	0.346	0.597	-0.005 (-0.016;0.006)
TG(60:3)	-0.949	0.343	0.597	-0.005 (-0.017;0.006)
11-HETE	-0.923	0.356	0.605	-0.005 (-0.015;0.005)
TG(52:2)	-0.922	0.356	0.605	-0.005 (-0.016;0.006)
Lysophosphatidylethanolamine (20:4)	0.905	0.365	0.615	0.005 (-0.006;0.016)
TG(56:4)	-0.904	0.366	0.615	-0.005 (-0.016;0.006)
Phosphatidylcholine(O-38:5)	-0.9	0.368	0.615	-0.005 (-0.017;0.006)
14,15-DiHETE	0.884	0.377	0.626	0.005 (-0.006;0.016)
1-AG/2-AG	0.865	0.387	0.64	0.005 (-0.006;0.015)
Pyroglutamic acid	0.835	0.404	0.664	0.005 (-0.006;0.016)
TG(48:1)	0.825	0.409	0.669	0.004 (-0.006;0.015)
Taurochenodeoxycholic acid	-0.816	0.415	0.674	-0.004 (-0.015;0.006)
Lysophosphatidylcholine(14:0)	0.81	0.418	0.676	0.005 (-0.007;0.016)
Serotonine	-0.788	0.431	0.689	-0.003 (-0.009;0.004)
3-Hydroxypropionic Acid	0.784	0.433	0.689	0.004 (-0.006;0.014)
Sphingosine 1-phosphate 18(1)	-0.783	0.434	0.689	-0.002 (-0.006;0.003)
Glycodeoxycholic acid	-0.778	0.437	0.689	-0.004 (-0.016;0.007)
Glycoursodeoxycholic acid	-0.776	0.438	0.689	-0.003 (-0.012;0.005)
FA 18(1)	0.766	0.444	0.693	0.004 (-0.007;0.015)
12,13-EpOME	-0.765	0.444	0.693	-0.005 (-0.016;0.007)
Phosphatidylcholine(38:5)	0.758	0.449	0.696	0.004 (-0.007;0.015)
L-Valine	-0.748	0.454	0.696	-0.004 (-0.015;0.007)
8(9)-EpETrE	-0.752	0.452	0.696	-0.002 (-0.008;0.004)
TG(56:7)	0.746	0.456	0.696	0.004 (-0.007;0.016)
Lactic acid	0.74	0.459	0.698	0.004 (-0.007;0.014)
Sphingosine 1-phosphate 18(0)	0.713	0.476	0.713	0.002 (-0.003;0.006)
Phosphatidylethanolamine(36:4)	0.719	0.472	0.713	0.004 (-0.007;0.014)
Sphingomyelin(d18:1/25:0)	-0.716	0.474	0.713	-0.004 (-0.016;0.007)
Deoxycholic acid	-0.7	0.484	0.721	-0.004 (-0.015;0.007)
Betaine	0.691	0.49	0.723	0.004 (-0.007;0.014)
Taurolithocholic acid	-0.69	0.49	0.723	-0.004 (-0.015;0.007)
Phosphatidylcholine(O-38:4)	-0.684	0.494	0.725	-0.004 (-0.015;0.007)
Gamma-L-glutamyl-L-alanine	-0.656	0.512	0.727	-0.004 (-0.014;0.007)
Sarcosine	-0.655	0.513	0.727	-0.003 (-0.013;0.007)
FA 22(5) w6	-0.668	0.504	0.727	-0.004 (-0.014;0.007)
(+/-) 14-HDoHE	0.659	0.51	0.727	0.001 (-0.003;0.005)
Phosphatidylcholine(32:1)	0.657	0.511	0.727	0.003 (-0.007;0.014)

Sphingomyelin(d18:1/16:1)	-0.652	0.514	0.727	-0.004 (-0.015;0.008)
TG(44:1)	0.655	0.512	0.727	0.003 (-0.006;0.012)
TG(46:2)	0.663	0.507	0.727	0.003 (-0.006;0.012)
TG(54:0)	-0.644	0.519	0.731	-0.004 (-0.016;0.008)
Lysophosphatidylinositol 18(1)	-0.634	0.526	0.733	-0.004 (-0.015;0.008)
TG(46:1)	0.637	0.524	0.733	0.003 (-0.006;0.013)
Linoleylcarnitine	-0.626	0.531	0.733	-0.003 (-0.012;0.006)
Phosphatidylcholine(36:1)	-0.627	0.531	0.733	-0.003 (-0.013;0.007)
Phosphatidylcholine(O-34:3)	-0.623	0.533	0.733	-0.004 (-0.015;0.008)
Phosphatidylcholine(34:4)	0.619	0.536	0.734	0.003 (-0.007;0.014)
DGLEA	0.586	0.558	0.761	0.003 (-0.008;0.014)
L-Alpha-aminobutyric acid	-0.572	0.568	0.761	-0.003 (-0.014;0.008)
Malonylcarnitine	0.561	0.575	0.761	0.003 (-0.008;0.014)
2-Ketoglutaric acid	0.569	0.57	0.761	0.003 (-0.007;0.013)
Phosphatidylcholine(32:2)	0.559	0.576	0.761	0.003 (-0.008;0.014)
Phosphatidylcholine(O-34:1)	0.566	0.572	0.761	0.003 (-0.008;0.014)
TG(54:1)	0.579	0.562	0.761	0.003 (-0.008;0.015)
TG(56:2)	-0.568	0.57	0.761	-0.003 (-0.013;0.007)
TG(48:2)	0.545	0.586	0.768	0.003 (-0.007;0.013)
TG(48:3)	0.547	0.585	0.768	0.003 (-0.007;0.013)
Glycylglycine	-0.53	0.596	0.774	-0.003 (-0.013;0.008)
(+/-) 11-HDoHE	0.526	0.599	0.774	0.002 (-0.005;0.008)
TG(42:0)	0.527	0.598	0.774	0.002 (-0.006;0.011)
Phosphatidylcholine(40:8)	-0.518	0.604	0.779	-0.002 (-0.012;0.007)
Taurocholic acid	0.51	0.61	0.783	0.003 (-0.008;0.013)
Dodecenoylcarnitine	0.497	0.619	0.788	0.003 (-0.008;0.013)
TG(44:0)	0.496	0.62	0.788	0.002 (-0.006;0.011)
1-LG/2-LG	-0.492	0.623	0.789	-0.003 (-0.013;0.008)
Sphingomyelin(d18:1/18:2)	0.482	0.63	0.795	0.003 (-0.009;0.015)
L-Phenylalanine	-0.435	0.663	0.795	-0.002 (-0.013;0.008)
Pimelylcarnitine	-0.422	0.673	0.795	-0.002 (-0.013;0.008)
FA 18(2)	0.413	0.68	0.795	0.002 (-0.008;0.012)
12,13-DiHODE	-0.436	0.663	0.795	-0.002 (-0.013;0.008)
12(S)-HEPE	0.452	0.652	0.795	0.001 (-0.003;0.005)
5-HETE	-0.467	0.641	0.795	-0.003 (-0.013;0.008)
Glycochenodeoxycholic acid	-0.407	0.684	0.795	-0.002 (-0.012;0.008)
Palmitoyl ethanolamide	0.442	0.659	0.795	0.003 (-0.009;0.014)
Cer(d18:0/22:0)	-0.47	0.639	0.795	-0.003 (-0.014;0.009)
DG(36:3)	-0.417	0.676	0.795	-0.002 (-0.012;0.008)
Phosphatidylcholine(34:1)	-0.412	0.681	0.795	-0.001 (-0.003;0.002)
Phosphatidylcholine(38:2)	-0.427	0.67	0.795	-0.002 (-0.013;0.009)
Phosphatidylcholine(O-36:3)	-0.438	0.661	0.795	-0.002 (-0.012;0.008)
TG(42:2)	-0.423	0.673	0.795	-0.002 (-0.012;0.008)

TG(44:2)	0.441	0.659	0.795	0.002 (-0.007;0.011)
TG(46:0)	0.413	0.679	0.795	0.002 (-0.007;0.011)
TG(50:1)	-0.424	0.672	0.795	-0.002 (-0.013;0.008)
TG(52:1)	-0.457	0.647	0.795	-0.002 (-0.013;0.008)
TG(53:1)	0.405	0.685	0.795	0.002 (-0.008;0.012)
TG(56:1)	0.45	0.653	0.795	0.002 (-0.007;0.011)
TG(57:1)	-0.433	0.665	0.795	-0.002 (-0.013;0.008)
L-Homoserine	0.379	0.704	0.814	0.002 (-0.009;0.014)
Phosphatidylethanolamine(34:2)	0.348	0.728	0.838	0.002 (-0.009;0.013)
Lysophosphatidylethanolamine(18:1)	-0.338	0.735	0.843	-0.002 (-0.011;0.008)
L-4-hydroxy-proline	0.33	0.741	0.844	0.002 (-0.009;0.012)
Lysophosphatidic acid 16(1)	0.331	0.74	0.844	0.002 (-0.008;0.011)
9,12,13-TriHOME	-0.322	0.747	0.844	-0.002 (-0.012;0.009)
Cer(d18:0/23:0)	0.325	0.745	0.844	0.002 (-0.009;0.013)
Butyrylcarnitine	-0.295	0.768	0.861	-0.002 (-0.013;0.009)
Phosphatidylcholine(O-36:5)	-0.295	0.768	0.861	-0.002 (-0.013;0.01)
L-Arginine	0.261	0.794	0.884	0.001 (-0.009;0.012)
CE(18:2)	-0.264	0.792	0.884	-0.001 (-0.013;0.01)
TG(42:1)	-0.248	0.804	0.889	-0.001 (-0.011;0.008)
TG(55:1)	-0.249	0.804	0.889	-0.002 (-0.013;0.01)
Phosphatidylcholine(40:5)	-0.21	0.833	0.915	-0.001 (-0.012;0.01)
TG(50:2)	-0.211	0.833	0.915	-0.001 (-0.011;0.009)
Phosphatidylethanolamine(O-36:5)	-0.206	0.836	0.915	-0.001 (-0.013;0.01)
TG(56:0)	0.178	0.859	0.936	0.001 (-0.01;0.012)
L-Proline	-0.169	0.866	0.936	-0.001 (-0.011;0.01)
DG(36:2)	0.172	0.864	0.936	0.001 (-0.009;0.011)
TG(45:0)	-0.154	0.878	0.946	-0.001 (-0.011;0.009)
L-Alanine	0.13	0.897	0.95	0.001 (-0.01;0.012)
Methylmalonylcarnitine	0.138	0.89	0.95	0.001 (-0.009;0.011)
9,10,13-TriHOME	0.132	0.895	0.95	0.001 (-0.01;0.011)
CE(20:4)	0.132	0.895	0.95	0.001 (-0.011;0.013)
TG(50:4)	-0.137	0.891	0.95	-0.001 (-0.012;0.01)
Cer(d18:1/22:0)	0.118	0.906	0.957	0.001 (-0.01;0.011)
Gamma-aminobutyric acid	-0.093	0.926	0.968	0 (-0.009;0.008)
N6,N6,N6-Trimethyl-L-lysine	-0.098	0.922	0.968	-0.001 (-0.011;0.01)
Taurodeoxycholic acid	0.089	0.929	0.968	0 (-0.01;0.011)
Lysophosphatidylcholine(22:6)	0.088	0.93	0.968	0.001 (-0.011;0.012)
Phosphatidylcholine(O-34:2)	0.078	0.938	0.969	0 (-0.011;0.011)
TG(50:3)	-0.078	0.938	0.969	0 (-0.011;0.01)
TG(54:7)	0.075	0.94	0.969	0 (-0.011;0.012)
L-Glutamine	-0.07	0.944	0.97	0 (-0.011;0.011)
Phosphatidylcholine(34:3)	0.066	0.948	0.97	0 (-0.011;0.011)
Cer(d18:1/24:1)	0.048	0.962	0.981	0 (-0.011;0.011)

Lysophosphatidylethanolamine (16:1)	0.043	0.966	0.982	0 (-0.01;0.011)
L-Lysine	-0.022	0.983	0.992	0 (-0.012;0.011)
Hydroxybutyric acid	0.026	0.98	0.992	0 (-0.011;0.011)
Tetradecenoylcarnitine	0.004	0.997	0.997	0 (-0.011;0.011)
Glycocholic acid	-0.011	0.991	0.997	0 (-0.01;0.01)
Phosphatidylethanolamine(O-38:5)	-0.007	0.995	0.997	0 (-0.012;0.012)

Shown are the results of the fixed effect meta-analysis: regression coefficient (CI: 95% confidence interval), nominal and adjusted p-values.

Table S4. Metabolites differently expressed in all IPD patients (n=140) compared to healthy controls (n=64)

Name	z-value	Nominal *p*	Adjusted *p*	Beta (CI)
3-Methoxytyrosine	13.376	8.38E-41	2.55E-38	1.514 (1.292;1.736)
Methyldopa	8.624	6.45E-18	9,800E-16	1.179 (0.911;1.447)
Putrescine	4.321	0.0000155	0.00157	0.5 (0.273;0.726)
(+/-) 16-HDoHE	3.906	0.000094	0.00714	0.629 (0.313;0.945)
Ornithine	03. Jun	0.000318	0.0194	0.516 (0.235;0.797)
L-Threonine	2.897	0.00377	0.191	0.464 (0.15;0.779)
TG(54:7)	-2.701	0.00692	0.301	-0.466 (-0.805;-0.128)
1-AG/2-AG	-2.589	0.00963	0.366	-0.413 (-0.726;-0.1)
TG(54:1)	-2.476	0.0133	0.378	-0.427 (-0.764;-0.089)
N6,N6,N6-Trimethyl-L-lysine	2.472	0.0134	0.378	0.378 (0.078;0.677)
Tiglylcarnitine	-2.465	0.0137	0.378	-0.406 (-0.728;-0.083)
Homocysteine	2.372	0.0177	0.399	0.353 (0.061;0.644)
TG(50:4)	-2.368	0.0179	0.399	-0.4 (-0.73;-0.069)
2-Ketoglutaric acid	-2.317	0.0205	0.399	-0.362 (-0.669;-0.056)
Phosphatidylcholine(38:4)	-2.287	0.0222	0.399	-0.405 (-0.751;-0.058)
Phosphatidylcholine(O-36:5)	-2.252	0.0243	0.399	-0.395 (-0.739;-0.051)
Methylmalonylcarnitine	-2.227	0.026	0.399	-0.341 (-0.641;-0.041)
Gamma-Glutamylglutamine	2.211	0.027	0.399	0.35 (0.04;0.661)
TG(54:5)	-2.208	0.0273	0.399	-0.386 (-0.729;-0.043)
Phosphatidylethanolamine(O-38:5)	-2.173	0.0298	0.399	-0.396 (-0.753;-0.039)
Phosphatidylcholine(38:7)	-2.171	0.0299	0.399	-0.378 (-0.719;-0.037)
Cystathionine	2.131	0.0331	0.399	0.286 (0.023;0.549)
TG(52:4)	-2.124	0.0337	0.399	-0.376 (-0.724;-0.029)
Phosphatidylcholine(36:4)	-2.112	0.0347	0.399	-0.369 (-0.712;-0.027)
TG(48:3)	-2.088	0.0368	0.399	-0.334 (-0.648;-0.02)
Glycoursodeoxycholic acid	-2.084	0.0371	0.399	-0.281 (-0.545;-0.017)
Phosphatidylethanolamine(O-36:5)	-2.063	0.0391	0.399	-0.372 (-0.726;-0.019)
TG(51:4)	-2.063	0.0391	0.399	-0.346 (-0.674;-0.017)
TG(56:6)	-2.061	0.0393	0.399	-0.362 (-0.706;-0.018)
Glycochenodeoxycholic acid	-2.06	0.0394	0.399	-0.308 (-0.6;-0.015)
Cer(d18:0/22:0)	-2.047	0.0407	0.399	-0.352 (-0.69;-0.015)
1-LG/2-LG	-1.946	0.0517	0.466	-0.307 (-0.615;0.002)
Saccharopine	1.934	0.0531	0.466	0.312 (-0.004;0.629)
CE(20:4)	-1.929	0.0537	0.466	-0.351 (-0.708;0.006)
2-Hydroxybutyric acid	-1.917	0.0552	0.466	-0.317 (-0.642;0.007)
Glycine	01. Sep	0.0574	0.466	0.297 (-0.009;0.604)
TG(51:3)	-1.878	0.0604	0.466	-0.309 (-0.633;0.014)
Phosphatidylcholine(O-38:5)	-1.877	0.0605	0.466	-0.346 (-0.706;0.015)
TG(50:3)	-1.873	0.0611	0.466	-0.315 (-0.644;0.015)
Phosphatidylcholine(40:5)	-1.871	0.0613	0.466	-0.32 (-0.655;0.015)

TG(51:2)	-1.852	0.064	0.474	-0.3 (-0.617;0.017)
TG(52:1)	-1.832	0.067	0.476	-0.297 (-0.615;0.021)
L-Tyrosine	1.819	0.069	0.476	0.281 (-0.022;0.584)
TG(48:2)	-1.818	0.069	0.476	-0.291 (-0.604;0.023)
TG(56:7)	-1.807	0.0708	0.476	-0.325 (-0.677;0.028)
TG(52:3)	-1.799	0.072	0.476	-0.315 (-0.658;0.028)
TG(48:1)	-1.785	0.0743	0.481	-0.285 (-0.599;0.028)
Phosphatidylcholine(O-36:4)	-1.749	0.0803	0.509	-0.314 (-0.667;0.038)
TG(58:2)	-1.709	0.0875	0.541	-0.305 (-0.655;0.045)
Glycocholic acid	-1.69	0.0911	0.541	-0.256 (-0.554;0.041)
Lysophosphatidic acid 16(0)	-1.689	0.0912	0.541	-0.243 (-0.525;0.039)
Isobutyrylcarnitine	-1.682	0.0925	0.541	-0.271 (-0.588;0.045)
TG(51:1)	-1.644	0.1	0.574	-0.259 (-0.568;0.05)
L-2-aminoadipic acid	-1.611	0.107	0.577	-0.242 (-0.536;0.052)
TG(50:1)	-1.607	0.108	0.577	-0.261 (-0.58;0.057)
Sphingomyelin(d18:1/15:0)	-1.596	0.111	0.577	-0.265 (-0.591;0.061)
9-HOTrE	1.585	0.113	0.577	0.26 (-0.062;0.582)
Phosphatidylcholine(34:4)	-1.585	0.113	0.577	-0.262 (-0.585;0.062)
Phosphatidylcholine(38:5)	-1.577	0.115	0.577	-0.272 (-0.611;0.066)
TG(54:4)	-1.575	0.115	0.577	-0.279 (-0.627;0.068)
Sphingomyelin(d18:1/18:1)	1.555	0.12	0.577	0.246 (-0.064;0.557)
Phosphatidylethanolamine(38:4)	-1.55	0.121	0.577	-0.251 (-0.569;0.066)
Lysophosphatidic acid 14(0)	-1.541	0.123	0.577	-0.218 (-0.496;0.059)
Phosphatidylcholine(O-38:6)	-1.542	0.123	0.577	-0.272 (-0.618;0.074)
Sphingomyelin(d18:1/20:1)	1.544	0.123	0.577	0.264 (-0.071;0.599)
Sphingomyelin(d18:1/23:0)	-1.532	0.126	0.577	-0.262 (-0.596;0.073)
Deoxycarnitine	-1.522	0.128	0.577	-0.183 (-0.418;0.053)
TG(50:2)	-1.518	0.129	0.577	-0.244 (-0.559;0.071)
Citric acid	1.498	0.134	0.591	0.217 (-0.067;0.501)
TG(52:2)	-1.45	0.147	0.622	-0.237 (-0.558;0.083)
L-Homoserine	1.447	0.148	0.622	0.241 (-0.085;0.567)
TG(46:2)	-1.44	0.15	0.622	-0.211 (-0.498;0.076)
TG(54:2)	-1.426	0.154	0.622	-0.23 (-0.545;0.086)
TG(56:1)	-1.425	0.154	0.622	-0.212 (-0.504;0.08)
Palmitoylcarnitine	Jan 42	0.155	0.622	0.231 (-0.088;0.55)
TG(56:5)	-1.423	0.155	0.622	-0.249 (-0.592;0.094)
DG(36:2)	-1.413	0.158	0.622	-0.224 (-0.536;0.087)
Phosphatidylethanolamine(38:2)	-1.401	0.161	0.626	-0.23 (-0.552;0.092)
TG(60:3)	-1.396	0.163	0.626	-0.246 (-0.592;0.099)
Choline	-1.381	0.167	0.629	-0.222 (-0.536;0.093)
Hexadecenoylcarnitine	1.377	0.169	0.629	0.222 (-0.094;0.539)
Sphingomyelin(d18:1/18:2)	-1.373	0.17	0.629	-0.269 (-0.654;0.115)
Gamma-aminobutyric acid	-1.358	0.174	0.637	-0.188 (-0.46;0.083)

TG(57:1)	-1.353	0.176	0.637	-0.229 (-0.561;0.103)
Phosphatidylcholine(40:7)	-1.313	0.189	0.655	-0.212 (-0.528;0.104)
TG(42:0)	-1.312	0.19	0.655	-0.187 (-0.467;0.092)
FA 22(5) w6	-1.306	0.192	0.655	-0.209 (-0.523;0.105)
Phosphatidylcholine(38:2)	-1.3	0.194	0.655	-0.22 (-0.552;0.112)
Phosphatidylcholine(38:3)	-1.295	0.195	0.655	-0.212 (-0.533;0.109)
3-Hydroxypropionic Acid	-1.293	0.196	0.655	-0.179 (-0.451;0.093)
TG(44:1)	-1.294	0.196	0.655	-0.186 (-0.468;0.096)
Thromboxane B2	-1.283	0.199	0.657	-0.186 (-0.469;0.098)
Serotonine	-1.277	0.202	0.657	-0.127 (-0.322;0.068)
TG(46:1)	-1.272	0.203	0.657	-0.194 (-0.494;0.105)
Phosphatidylethanolamine(O-38:7)	-1.266	0.205	0.657	-0.219 (-0.557;0.12)
15(S)-HETrE	1.228	0.219	0.69	0.159 (-0.094;0.412)
TG(54:3)	-1.208	0.227	0.69	-0.206 (-0.541;0.128)
TG(58:9)	-1.2	0.23	0.69	-0.203 (-0.533;0.128)
Phosphatidylcholine(36:3)	-1.177	0.239	0.69	-0.195 (-0.52;0.13)
Ethanolamine	1.172	0.241	0.69	0.176 (-0.118;0.47)
Cer(d18:1/23:0)	-1.172	0.241	0.69	-0.196 (-0.525;0.132)
Phosphatidylcholine(32:0)	-1.168	0.243	0.69	-0.202 (-0.541;0.137)
Methionine sulfone	-1.162	0.245	0.69	-0.19 (-0.511;0.131)
Sphingosine 1-phosphate 18(2)	1.159	0.247	0.69	0.115 (-0.079;0.308)
Hexanoylcarnitine	1.155	0.248	0.69	0.193 (-0.135;0.52)
Sphingomyelin(d18:1/18:0)	1.152	0.249	0.69	0.186 (-0.131;0.503)
Isocitrate	-1.149	0.251	0.69	-0.149 (-0.404;0.105)
8(9)-EpETrE	1.138	0.255	0.69	0.105 (-0.076;0.287)
TG(42:1)	-1.139	0.255	0.69	-0.172 (-0.469;0.124)
Lysophosphatidylinositol 16(1)	1.133	0.257	0.69	0.183 (-0.133;0.499)
Lysophosphatidylinositol 18(1)	1.134	0.257	0.69	0.192 (-0.139;0.523)
Taurocholic acid	-1.135	0.257	0.69	-0.181 (-0.495;0.132)
O-Acetyl-L-serine	-1.13	0.259	0.69	-0.172 (-0.47;0.126)
Phosphatidylcholine(40:6)	-1.129	0.259	0.69	-0.187 (-0.511;0.137)
TG(60:1)	-1.113	0.266	0.696	-0.192 (-0.531;0.146)
Cer(d18:1/22:0)	-1.103	0.27	0.696	-0.176 (-0.49;0.137)
17,18-DiHETE	01. Jan	0.271	0.696	0.18 (-0.14;0.5)
Lysophosphatidylethanolamine (16:1)	1.091	0.275	0.696	0.169 (-0.135;0.473)
Octanoylcarnitine	1.074	0.283	0.696	0.178 (-0.147;0.504)
Lysophosphatidylcholine(O-18:1)	-1.065	0.287	0.696	-0.172 (-0.487;0.144)
TG(44:2)	-1.064	0.287	0.696	-0.159 (-0.451;0.133)
TG(55:1)	-1.063	0.288	0.696	-0.198 (-0.562;0.167)
TG(42:2)	-1.052	0.293	0.696	-0.165 (-0.473;0.142)
Lysophosphatidylinositol 20(4)	1.049	0.294	0.696	0.166 (-0.145;0.477)
Phosphatidylethanolamine(36:4)	-1.05	0.294	0.696	-0.168 (-0.482;0.146)
Sphingomyelin(d18:1/23:1)	-1.049	0.294	0.696	-0.173 (-0.495;0.15)

Decanoylcarnitine	1.048	0.295	0.696	0.171 (-0.149;0.491)
Phosphatidylcholine(38:6)	-1.047	0.295	0.696	-0.173 (-0.497;0.151)
TG(60:2)	-1.036	0.3	0.696	-0.183 (-0.528;0.163)
11-HETE	1.031	0.303	0.696	0.153 (-0.138;0.444)
Nonaylcarnitine	-1.026	0.305	0.696	-0.165 (-0.479;0.15)
12,13-DiHOME	1.026	0.305	0.696	0.167 (-0.152;0.486)
2-Methylbutyroylcarnitine	-1.02	0.308	0.696	-0.153 (-0.446;0.141)
Phosphatidylcholine(34:3)	-1.017	0.309	0.696	-0.173 (-0.507;0.161)
Tetradecenoylcarnitine	1.012	0.311	0.696	0.167 (-0.157;0.492)
Phosphatidylethanolamine(34:2)	-1.013	0.311	0.696	-0.171 (-0.503;0.16)
Propionylcarnitine	-1	0.317	0.7	-0.15 (-0.444;0.144)
FA 22(6)	-0.998	0.318	0.7	-0.161 (-0.477;0.155)
FA 18(1)	0.992	0.321	0.7	0.162 (-0.159;0.483)
1-Methylhistidine	-0.985	0.325	0.7	-0.156 (-0.467;0.155)
Dodecenoylcarnitine	0.985	0.325	0.7	0.159 (-0.157;0.475)
TG(44:0)	-0.981	0.327	0.7	-0.137 (-0.41;0.137)
Myristoilcarnitine	0.968	0.333	0.705	0.156 (-0.16;0.473)
Lysophosphatidylethanolamine(18:0)	-0.967	0.334	0.705	-0.163 (-0.494;0.168)
Sphingomyelin(d18:1/21:0)	-0.95	0.342	0.711	-0.164 (-0.501;0.174)
TG(56:3)	-0.947	0.344	0.711	-0.157 (-0.483;0.168)
Oleoylcarnitine	0.941	0.347	0.711	0.153 (-0.166;0.472)
Pyroglutamic acid	-0.937	0.349	0.711	-0.158 (-0.487;0.172)
LPS 18(1)	-0.937	0.349	0.711	-0.092 (-0.284;0.1)
11,12-DiHETrE	-0.927	0.354	0.718	-0.147 (-0.458;0.164)
Deoxycholic acid	0.918	0.359	0.722	0.154 (-0.174;0.482)
DGLEA	-0.909	0.363	0.722	-0.149 (-0.47;0.172)
TG(46:0)	-0.909	0.363	0.722	-0.137 (-0.433;0.159)
DG(36:3)	-0.905	0.366	0.722	-0.145 (-0.46;0.169)
Phosphatidylcholine(40:8)	-0.894	0.371	0.725	-0.129 (-0.411;0.153)
TG(53:1)	-0.891	0.373	0.725	-0.141 (-0.45;0.169)
Homocitrulline	-0.888	0.374	0.725	-0.148 (-0.475;0.179)
12,13-EpOME	0.863	0.388	0.741	0.154 (-0.195;0.503)
Trimethylamine N-oxide	0.857	0.391	0.741	0.143 (-0.185;0.472)
9,10-DiHOME	0.855	0.393	0.741	0.131 (-0.169;0.43)
TG(56:4)	-0.852	0.394	0.741	-0.148 (-0.488;0.192)
Sphingosine 1-phosphate 18(0)	-0.845	0.398	0.741	-0.06 (-0.199;0.079)
Glycylglycine	0.841	0.4	0.741	0.134 (-0.178;0.445)
8,12-iPF2a IV	-0.842	0.4	0.741	-0.119 (-0.397;0.159)
Betaine	-0.838	0.402	0.741	-0.129 (-0.432;0.173)
Lysophosphatidylcholine(O-16:0)	-0.823	0.411	0.748	-0.142 (-0.482;0.197)
Lysophosphatidylcholine(O-16:1)	-0.822	0.411	0.748	-0.145 (-0.491;0.201)
Lauroylcarnitine	0.812	0.417	0.751	0.134 (-0.189;0.457)
Cer(d18:0/23:0)	-0.806	0.42	0.751	-0.138 (-0.474;0.198)

Sphingomyelin(d18:1/16:0)	-0.806	0.42	0.751	-0.147 (-0.504;0.21)
Phosphatidylcholine(O-44:5)	0.8	0.424	0.751	0.142 (-0.206;0.491)
Lysophosphatidylcholine(16:1)	0.794	0.427	0.751	0.136 (-0.199;0.471)
Phosphatidylethanolamine(38:6)	-0.793	0.428	0.751	-0.125 (-0.432;0.183)
L-Kynurenine	-0.783	0.433	0.755	-0.131 (-0.457;0.196)
9,12,13-TriHOME	0.781	0.435	0.755	0.122 (-0.185;0.43)
Phosphatidylcholine(36:5)	-0.777	0.437	0.755	-0.129 (-0.454;0.196)
Lysophosphatidylcholine(16:0)	-0.762	0.446	0.761	-0.133 (-0.475;0.209)
Phosphatidylcholine(32:2)	-0.756	0.45	0.761	-0.13 (-0.466;0.206)
5,6-DiHETrE	-0.752	0.452	0.761	-0.121 (-0.435;0.194)
Stearoylcarnitine	0.743	0.457	0.761	0.114 (-0.186;0.414)
Taurodeoxycholic acid	-0.744	0.457	0.761	-0.117 (-0.426;0.191)
9,10,13-TriHOME	0.74	0.459	0.761	0.114 (-0.187;0.414)
Lysophosphatidylcholine(18:0)	-0.734	0.463	0.761	-0.131 (-0.48;0.218)
Prostaglandin E2	-0.73	0.465	0.761	-0.108 (-0.399;0.182)
Phosphatidylcholine(36:2)	-0.731	0.465	0.761	-0.125 (-0.46;0.21)
Lysophosphatidylcholine(20:4)	-0.729	0.466	0.761	-0.123 (-0.455;0.208)
Lysophosphatidylethanolamine(18:1)	0.717	0.473	0.769	0.105 (-0.182;0.391)
Sarcosine	0.712	0.477	0.771	0.106 (-0.186;0.399)
S-Methylcysteine	-0.705	0.481	0.774	-0.116 (-0.439;0.207)
Lysophosphatidylinositol 18(2)	0.7	0.484	0.774	0.116 (-0.209;0.441)
TG(56:2)	-0.688	0.491	0.779	-0.107 (-0.413;0.198)
L-4-hydroxy-proline	0.681	0.496	0.779	0.108 (-0.203;0.42)
L-Methionine	-0.68	0.496	0.779	-0.099 (-0.385;0.186)
TG(58:8)	-0.679	0.497	0.779	-0.119 (-0.461;0.224)
L-Glutamine	0.67	0.503	0.784	0.11 (-0.212;0.432)
Citrulline	-0.664	0.507	0.786	-0.11 (-0.435;0.215)
CE(18:2)	-0.654	0.513	0.792	-0.111 (-0.445;0.222)
Lysophosphatidylcholine(14:0)	-0.629	0.529	0.809	-0.111 (-0.456;0.235)
Sphingomyelin(d18:1/25:1)	-0.624	0.532	0.809	-0.103 (-0.427;0.221)
TG(56:0)	-0.624	0.532	0.809	-0.106 (-0.439;0.227)
8,9-DiHETrE	-0.603	0.546	0.826	-0.093 (-0.393;0.208)
Lysophosphatidylethanolamine (20:4)	-0.592	0.554	0.834	-0.095 (-0.412;0.221)
(+/-) 8-HDoHE	0.587	0.557	0.835	0.103 (-0.241;0.446)
Lysophosphatidylcholine(18:1)	0.581	0.561	0.836	0.101 (-0.238;0.439)
Linoleoyl ethanolamide	-0.574	0.566	0.839	-0.094 (-0.413;0.226)
Cholic acid	-0.559	0.576	0.85	-0.09 (-0.404;0.225)
Acetylcarnitine	-0.555	0.579	0.85	-0.091 (-0.413;0.231)
Taurolithocholic acid	-0.55	0.583	0.852	-0.092 (-0.419;0.235)
Taurochenodeoxycholic acid	0.544	0.586	0.853	0.088 (-0.23;0.406)
Dehydroepiandrosterone	-0.531	0.595	0.862	-0.086 (-0.405;0.232)
Sphingomyelin(d18:1/14:0)	-0.525	0.6	0.863	-0.091 (-0.429;0.248)
L-Glutamic acid	-0.522	0.602	0.863	-0.08 (-0.38;0.22)

L-Alpha-aminobutyric acid	-0.502	0.615	0.873	-0.082 (-0.404;0.239)
L-Leucine	-0.5	0.617	0.873	-0.075 (-0.369;0.219)
L-Tryptophan	-0.495	0.621	0.873	-0.078 (-0.386;0.231)
9-HODE	0.494	0.621	0.873	0.075 (-0.223;0.374)
Glutathione	-0.487	0.626	0.873	-0.065 (-0.327;0.197)
Palmitoyl ethanolamide	0.487	0.626	0.873	0.083 (-0.251;0.417)
Phosphatidylcholine(O-34:2)	0.481	0.631	0.874	0.079 (-0.242;0.399)
Phosphatidylcholine(32:1)	0.474	0.636	0.874	0.076 (-0.238;0.391)
FA 18(2)	-0.466	0.641	0.874	-0.068 (-0.354;0.218)
Phosphatidylcholine(34:2)	-0.465	0.642	0.874	-0.08 (-0.419;0.258)
Prostaglandin F2α	0.458	0.647	0.874	0.061 (-0.201;0.324)
Lactic acid	-0.455	0.649	0.874	-0.073 (-0.389;0.242)
13-HODE	0.454	0.65	0.874	0.061 (-0.204;0.327)
19,20-DiHDPA	0.454	0.65	0.874	0.073 (-0.242;0.387)
L-Alanine	-0.447	0.655	0.877	-0.075 (-0.403;0.254)
L-Histidine	-0.443	0.658	0.877	-0.075 (-0.405;0.255)
Sphingomyelin(d18:1/24:1)	-0.42	0.675	0.895	-0.075 (-0.423;0.273)
Sphingomyelin(d18:1/16:1)	-0.411	0.681	0.896	-0.071 (-0.412;0.269)
5-HETE	0.41	0.682	0.896	0.066 (-0.249;0.38)
Gamma-L-glutamyl-L-alanine	0.404	0.686	0.896	0.065 (-0.251;0.382)
Lysophosphatidylcholine(18:2)	0.404	0.687	0.896	0.069 (-0.267;0.405)
Cortisol	-0.397	0.692	0.899	-0.061 (-0.365;0.242)
Sphingomyelin(d18:1/25:0)	-0.391	0.696	0.9	-0.07 (-0.42;0.281)
12,13-DiHODE	-0.381	0.703	0.905	-0.061 (-0.377;0.254)
Sphingomyelin(d18:1/20:0)	0.374	0.708	0.908	0.064 (-0.271;0.398)
Carnitine	0.369	0.712	0.91	0.058 (-0.248;0.363)
L-Lysine	0.356	0.722	0.915	0.06 (-0.27;0.39)
Lysophosphatidylethanolamine(22:6)	-0.353	0.724	0.915	-0.058 (-0.381;0.265)
Lysophosphatidic acid 18(1)	0.351	0.726	0.915	0.053 (-0.244;0.35)
3-Hydroxybutyric acid	-0.347	0.729	0.915	-0.06 (-0.4;0.28)
Lysophosphatidic acid 18(2)	-0.344	0.731	0.915	-0.054 (-0.36;0.253)
Phosphatidylcholine(O-34:3)	-0.335	0.738	0.917	-0.057 (-0.39;0.276)
Lysophosphatidylethanolamine (20:5)	0.333	0.739	0.917	0.054 (-0.264;0.372)
CE(18:1)	-0.329	0.742	0.917	-0.057 (-0.396;0.282)
Taurine	-0.316	0.752	0.925	-0.026 (-0.189;0.137)
Cer(d18:1/24:1)	-0.31	0.756	0.927	-0.055 (-0.401;0.291)
14,15-DiHETE	0.306	0.76	0.928	0.05 (-0.273;0.374)
Sphingomyelin(d18:1/22:0)	-0.296	0.767	0.931	-0.05 (-0.384;0.283)
Phosphatidylcholine(36:1)	0.294	0.769	0.931	0.046 (-0.262;0.355)
L-Valine	-0.275	0.783	0.941	-0.044 (-0.358;0.27)
Tetradecadienylcarnitine	0.275	0.783	0.941	0.045 (-0.275;0.364)
Decenoylcarnitine	0.266	0.79	0.943	0.044 (-0.28;0.368)
TG(54:0)	-0.262	0.793	0.943	-0.048 (-0.41;0.313)

CE(22:6)	-0.257	0.797	0.943	-0.041 (-0.357;0.274)
L-Asparagine	-0.256	0.798	0.943	-0.04 (-0.347;0.267)
Cysteine	-0.253	0.801	0.943	-0.037 (-0.322;0.248)
Malic acid	-0.237	0.813	0.954	-0.035 (-0.327;0.256)
Lysophosphatidylcholine(20:5)	0.224	0.823	0.955	0.033 (-0.253;0.318)
Symmetric dimethylarginine	0.218	0.828	0.955	0.035 (-0.276;0.345)
Sphingomyelin(d18:1/24:0)	-0.212	0.832	0.955	-0.037 (-0.374;0.301)
Valerylcarnitine	-0.205	0.837	0.955	-0.033 (-0.346;0.28)
L-Phenylalanine	-0.205	0.838	0.955	-0.032 (-0.339;0.275)
Glycolithocholic acid	-0.203	0.839	0.955	-0.034 (-0.361;0.294)
Lysophosphatidylethanolamine(20:4)	-0.198	0.843	0.955	-0.034 (-0.37;0.302)
L-Proline	-0.196	0.844	0.955	-0.031 (-0.339;0.277)
12-HETE	0.196	0.845	0.955	0.014 (-0.125;0.153)
DL-3-aminoisobutyric acid	0.193	0.847	0.955	0.031 (-0.284;0.346)
Sphingomyelin(d18:1/24:2)	0.188	0.851	0.955	0.033 (-0.31;0.376)
L-Serine	0.18	0.857	0.955	0.029 (-0.29;0.349)
Lysophosphatidylcholine(22:6)	0.177	0.859	0.955	0.03 (-0.307;0.367)
Hydroxylysine	-0.172	0.863	0.955	-0.029 (-0.361;0.302)
Glycodeoxycholic acid	-0.17	0.865	0.955	-0.028 (-0.356;0.3)
Phosphatidylcholine(O-38:4)	-0.167	0.867	0.955	-0.028 (-0.358;0.302)
Butyrylcarnitine	-0.167	0.868	0.955	-0.027 (-0.349;0.294)
O-Phosphoethanolamine	-0.163	0.87	0.955	-0.01 (-0.136;0.115)
Phosphatidylcholine(O-34:1)	0.15	0.881	0.963	0.025 (-0.301;0.351)
Isovalerylcarnitine	-0.133	0.894	0.974	-0.02 (-0.314;0.274)
Phosphatidylcholine(34:1)	0.126	0.9	0.977	0.005 (-0.08;0.091)
O-Anandamide	-0.11	0.913	0.983	-0.019 (-0.35;0.313)
Pimelylcarnitine	-0.104	0.917	0.983	-0.016 (-0.325;0.293)
Linoleylcarnitine	-0.101	0.92	0.983	-0.014 (-0.284;0.256)
Lysophosphatidylcholine(20:3)	0.097	0.923	0.983	0.016 (-0.313;0.346)
Phosphatidylcholine(O-36:3)	0.095	0.924	0.983	0.014 (-0.285;0.314)
TG(45:0)	-0.095	0.925	0.983	-0.015 (-0.332;0.301)
14,15-DiHETrE	-0.087	0.931	0.986	-0.014 (-0.33;0.302)
8-HETE	-0.08	0.936	0.988	-0.01 (-0.244;0.225)
Octenoylcarnitine	0.067	0.946	0.99	0.011 (-0.316;0.339)
Malonylcarnitine	-0.063	0.949	0.99	-0.01 (-0.326;0.306)
L-Arginine	0.062	0.95	0.99	0.01 (-0.309;0.33)
Lysophosphatidylethanolamine (16:0)	0.06	0.952	0.99	0.01 (-0.313;0.333)
Sphingosine 1-phosphate 18(1)	0.057	0.954	0.99	0.004 (-0.131;0.138)
(+/-) 11-HDoHE	-0.043	0.966	0.993	-0.004 (-0.194;0.186)
TG(59:1)	-0.04	0.968	0.993	-0.007 (-0.355;0.34)
Lysophosphatidic acid 16(1)	-0.039	0.969	0.993	-0.005 (-0.279;0.268)
(+/-) 10-HDoHE	0.037	0.971	0.993	0.003 (-0.179;0.186)
Anandamide	0.03	0.976	0.995	0.005 (-0.324;0.334)

Lysophosphatidylinositol 18(0)	-0.026	0.979	0.995	-0.004 (-0.297;0.289)
L-Isoleucine	-0.008	0.994	1	-0.001 (-0.304;0.301)
(+/-) 14-HDoHE	-0.008	0.994	1	-0.001 (-0.127;0.126)
Lysophosphatidylcholine(18:3)	0.005	0.996	1	0.001 (-0.361;0.362)
12(S)-HEPE	-0.002	0.998	1	0 (-0.134;0.134)
Sphingomyelin(d18:1/22:1)	0	1	1	0 (-0.337;0.337)

Shown are the results of the fixed effect meta-analysis: regression coefficient (CI: 95% confidence interval), nominal and adjusted p-values.

Table S5. Metabolites differently expressed in IPD patients with L-Dopa treatment (L-Dopa^{positive}, n=110) compared to controls (n=64)

Name	z-value	Nominal *p*	Adjusted *p*	Beta (CI)
3-Methoxytyrosine	1.95	0	0	1.95 (1.873;2.026)
Methyldopa	1.571	6.36e-40	9.67e-38	1.571 (1.339;1.804)
(+/-) 16-HDoHE	0.809	2.24e-06	0.000227	0.809 (0.474;1.144)
Putrescine	0.547	6.39e-06	0.000485	0.547 (0.309;0.784)
Ornithinie	0.582	6.36e-05	0.00387	0.582 (0.297;0.868)
N6,N6,N6-Trimethyl-L-lysine	0.493	0.00265	0.135	0.493 (0.171;0.814)
L-Threonine	0.486	0.00366	0.159	0.486 (0.158;0.813)
Homocysteine	0.403	0.00949	0.309	0.403 (0.099;0.708)
L-Tyrosine	0.416	0.0102	0.309	0.416 (0.099;0.734)
2-Ketoglutaric acid	-0.424	0.00907	0.309	-0.424 (-0.742;-0.105)
TG(54:7)	-0.446	0.0144	0.398	-0.446 (-0.803;-0.089)
TG(54:1)	-0.39	0.0161	0.407	-0.39 (-0.708;-0.073)
Methylmalonylcarnitine	-0.367	0.0176	0.41	-0.367 (-0.67;-0.064)
Cystathionine	0.324	0.0196	0.425	0.324 (0.052;0.597)
Gamma-Glutamylglutamine	0.377	0.0232	0.47	0.377 (0.052;0.703)
Glycocholic acid	-0.321	0.0301	0.571	-0.321 (-0.611;-0.031)
Glycochenodeoxycholic acid	-0.345	0.0319	0.571	-0.345 (-0.66;-0.03)
Deoxycarnitine	-0.266	0.0346	0.584	-0.266 (-0.513;-0.019)
Saccharopine	0.354	0.0403	0.625	0.354 (0.016;0.692)
Hydroxybutyric acid	-0.361	0.0425	0.625	-0.361 (-0.711;-0.012)
Glycoursodeoxycholic acid	-0.295	0.0473	0.625	-0.295 (-0.587;-0.004)
Cer(d18:0/22:0)	-0.345	0.0444	0.625	-0.345 (-0.68;-0.009)
Phosphatidylcholine(38:4)	-0.375	0.0456	0.625	-0.375 (-0.742;-0.007)
Tiglylcarnitine	-0.336	0.0503	0.63	-0.336 (-0.673;0)
TG(50:4)	-0.352	0.0518	0.63	-0.352 (-0.708;0.003)
1-Methylhistidine	-0.232	0.175	0.634	-0.232 (-0.568;0.103)
Ethanolamine	0.204	0.18	0.634	0.204 (-0.095;0.503)
Gamma-aminobutyric acid	-0.216	0.145	0.634	-0.216 (-0.506;0.074)
Glycine	0.322	0.061	0.634	0.322 (-0.015;0.659)
Glycylglycine	0.222	0.172	0.634	0.222 (-0.096;0.54)
L-Homoserine	0.249	0.172	0.634	0.249 (-0.108;0.606)
Methionine sulfone	-0.265	0.144	0.634	-0.265 (-0.619;0.09)
Serotonine	-0.2	0.0703	0.634	-0.2 (-0.417;0.017)
Betaine	-0.221	0.154	0.634	-0.221 (-0.524;0.082)
Choline	-0.25	0.149	0.634	-0.25 (-0.59;0.09)
3-Hydroxypropionic Acid	-0.229	0.128	0.634	-0.229 (-0.523;0.066)
FA 22(5) w6	-0.268	0.119	0.634	-0.268 (-0.605;0.069)
FA 22(6)	-0.256	0.121	0.634	-0.256 (-0.58;0.068)
Lysophosphatidylethanolamine (16:1)	0.249	0.123	0.634	0.249 (-0.067;0.566)
Lysophosphatidylinositol 16(1)	0.25	0.156	0.634	0.25 (-0.095;0.595)

Sphingosine 1-phosphate 18(2)	0.148	0.169	0.634	0.148 (-0.063;0.358)
Taurocholic acid	-0.21	0.188	0.634	-0.21 (-0.522;0.102)
1-AG/2-AG	-0.287	0.0936	0.634	-0.287 (-0.623;0.049)
11-HETE	0.214	0.175	0.634	0.214 (-0.095;0.523)
15(S)-HETrE	0.199	0.147	0.634	0.199 (-0.07;0.467)
9-HOTrE	0.291	0.102	0.634	0.291 (-0.057;0.639)
Deoxycholic acid	0.292	0.0838	0.634	0.292 (-0.039;0.623)
CE(20:4)	-0.325	0.0868	0.634	-0.325 (-0.698;0.047)
Cer(d18:1/23:0)	-0.229	0.189	0.634	-0.229 (-0.572;0.113)
Phosphatidylcholine(34:4)	-0.253	0.164	0.634	-0.253 (-0.61;0.103)
Phosphatidylcholine(36:4)	-0.256	0.169	0.634	-0.256 (-0.622;0.109)
Phosphatidylcholine(38:2)	-0.242	0.188	0.634	-0.242 (-0.601;0.118)
Phosphatidylcholine(38:5)	-0.29	0.0929	0.634	-0.29 (-0.628;0.048)
Phosphatidylcholine(38:6)	-0.218	0.176	0.634	-0.218 (-0.534;0.098)
Phosphatidylcholine(38:7)	-0.299	0.111	0.634	-0.299 (-0.666;0.069)
Phosphatidylcholine(40:5)	-0.303	0.0803	0.634	-0.303 (-0.643;0.037)
Phosphatidylcholine(40:6)	-0.255	0.117	0.634	-0.255 (-0.575;0.064)
Phosphatidylcholine(40:7)	-0.267	0.0997	0.634	-0.267 (-0.585;0.051)
Phosphatidylcholine(O-36:4)	-0.329	0.0938	0.634	-0.329 (-0.713;0.056)
Phosphatidylcholine(O-36:5)	-0.342	0.0744	0.634	-0.342 (-0.717;0.034)
Phosphatidylcholine(O-38:5)	-0.369	0.0672	0.634	-0.369 (-0.765;0.026)
Phosphatidylcholine(O-38:6)	-0.338	0.0627	0.634	-0.338 (-0.694;0.018)
Phosphatidylethanolamine(O-36:5)	-0.3	0.115	0.634	-0.3 (-0.674;0.074)
Phosphatidylethanolamine(O-38:5)	-0.328	0.0871	0.634	-0.328 (-0.704;0.048)
Phosphatidylethanolamine(O-38:7)	-0.248	0.162	0.634	-0.248 (-0.595;0.1)
Sphingomyelin(d18:1/15:0)	-0.279	0.121	0.634	-0.279 (-0.632;0.074)
Sphingomyelin(d18:1/18:1)	0.272	0.112	0.634	0.272 (-0.063;0.607)
Sphingomyelin(d18:1/18:2)	-0.321	0.124	0.634	-0.321 (-0.729;0.088)
Sphingomyelin(d18:1/20:1)	0.253	0.167	0.634	0.253 (-0.106;0.612)
Sphingomyelin(d18:1/23:0)	-0.26	0.151	0.634	-0.26 (-0.616;0.095)
TG(46:2)	-0.222	0.174	0.634	-0.222 (-0.541;0.098)
TG(48:1)	-0.27	0.122	0.634	-0.27 (-0.612;0.072)
TG(48:2)	-0.268	0.124	0.634	-0.268 (-0.61;0.073)
TG(48:3)	-0.331	0.0575	0.634	-0.331 (-0.672;0.011)
TG(50:1)	-0.24	0.173	0.634	-0.24 (-0.586;0.105)
TG(50:3)	-0.277	0.128	0.634	-0.277 (-0.634;0.08)
TG(51:1)	-0.228	0.19	0.634	-0.228 (-0.569;0.113)
TG(51:2)	-0.296	0.0963	0.634	-0.296 (-0.645;0.053)
TG(51:3)	-0.272	0.136	0.634	-0.272 (-0.629;0.085)
TG(51:4)	-0.334	0.0724	0.634	-0.334 (-0.698;0.03)
TG(52:1)	-0.26	0.138	0.634	-0.26 (-0.603;0.084)
TG(52:3)	-0.25	0.188	0.634	-0.25 (-0.623;0.122)
TG(52:4)	-0.302	0.116	0.634	-0.302 (-0.678;0.074)

TG(54:5)	-0.315	0.0952	0.634	-0.315 (-0.684;0.055)
TG(56:1)	-0.301	0.0703	0.634	-0.301 (-0.627;0.025)
TG(56:6)	-0.295	0.113	0.634	-0.295 (-0.66;0.07)
TG(56:7)	-0.282	0.132	0.634	-0.282 (-0.65;0.085)
TG(57:1)	-0.331	0.0763	0.634	-0.331 (-0.698;0.035)
TG(58:2)	-0.335	0.093	0.634	-0.335 (-0.727;0.056)
TG(60:1)	-0.256	0.177	0.634	-0.256 (-0.628;0.116)
TG(60:3)	-0.278	0.139	0.634	-0.278 (-0.647;0.09)
Glutathione	-0.184	0.195	0.64	-0.184 (-0.463;0.094)
L-Methionine	-0.192	0.196	0.64	-0.192 (-0.482;0.099)
L-2-aminoadipic acid	-0.21	0.201	0.65	-0.21 (-0.532;0.112)
Phosphatidylethanolamine(38:2)	-0.215	0.206	0.658	-0.215 (-0.549;0.119)
TG(50:2)	-0.22	0.208	0.658	-0.22 (-0.562;0.122)
Isobutyrylcarnitine	-0.212	0.219	0.681	-0.212 (-0.55;0.126)
Sphingomyelin(d18:1/18:0)	0.212	0.219	0.681	0.212 (-0.126;0.55)
Isocitrate	-0.168	0.224	0.687	-0.168 (-0.439;0.103)
Acetylcarnitine	-0.205	0.238	0.695	-0.205 (-0.546;0.135)
8(9)-EpETrE	0.111	0.235	0.695	0.111 (-0.072;0.294)
DG(36:2)	-0.214	0.236	0.695	-0.214 (-0.568;0.14)
TG(42:0)	-0.193	0.237	0.695	-0.193 (-0.512;0.127)
TG(52:2)	-0.211	0.235	0.695	-0.211 (-0.558;0.137)
Linoleoyl ethanolamide	-0.199	0.24	0.696	-0.199 (-0.531;0.133)
O-Acetyl-L-serine	-0.183	0.257	0.697	-0.183 (-0.499;0.133)
Palmitoylcarnitine	0.199	0.253	0.697	0.199 (-0.142;0.539)
Lysophosphatidic acid 14(0)	-0.169	0.262	0.697	-0.169 (-0.464;0.126)
Lysophosphatidylinositol 18(1)	0.213	0.25	0.697	0.213 (-0.15;0.575)
1-LG/2-LG	-0.192	0.264	0.697	-0.192 (-0.528;0.144)
11,12-DiHETrE	-0.195	0.249	0.697	-0.195 (-0.526;0.136)
9,10-DiHOME	0.179	0.262	0.697	0.179 (-0.134;0.493)
Phosphatidylethanolamine(38:4)	-0.198	0.248	0.697	-0.198 (-0.535;0.138)
TG(44:1)	-0.183	0.256	0.697	-0.183 (-0.499;0.133)
TG(54:2)	-0.202	0.253	0.697	-0.202 (-0.547;0.144)
TG(55:1)	-0.218	0.27	0.708	-0.218 (-0.606;0.17)
TG(54:4)	-0.209	0.278	0.723	-0.209 (-0.588;0.169)
Citrulline	-0.195	0.283	0.726	-0.195 (-0.552;0.161)
Homocitrulline	-0.193	0.284	0.726	-0.193 (-0.546;0.16)
Phosphatidylcholine(32:0)	-0.193	0.295	0.741	-0.193 (-0.554;0.168)
TG(46:1)	-0.177	0.293	0.741	-0.177 (-0.507;0.153)
Lysophosphatidylcholine(O-18:1)	-0.182	0.298	0.742	-0.182 (-0.523;0.16)
Sphingomyelin(d18:1/23:1)	-0.184	0.305	0.745	-0.184 (-0.535;0.167)
TG(42:1)	-0.173	0.306	0.745	-0.173 (-0.504;0.158)
TG(60:2)	-0.196	0.306	0.745	-0.196 (-0.571;0.179)
12,13-DiHOME	0.179	0.313	0.749	0.179 (-0.168;0.525)

Thromboxane-B2	-0.158	0.311	0.749	-0.158 (-0.464;0.148)
Lysophosphatidylinositol 20(4)	0.163	0.319	0.758	0.163 (-0.158;0.485)
Decanoylcarnitine	0.166	0.331	0.766	0.166 (-0.168;0.499)
Nonaylcarnitine	-0.152	0.343	0.766	-0.152 (-0.467;0.163)
Dehydroepiandrosterone	-0.163	0.334	0.766	-0.163 (-0.495;0.168)
Lysophosphatidylcholine(16:1)	0.175	0.336	0.766	0.175 (-0.181;0.531)
TG(42:2)	-0.17	0.337	0.766	-0.17 (-0.516;0.177)
TG(44:2)	-0.158	0.343	0.766	-0.158 (-0.483;0.168)
TG(54:3)	-0.177	0.343	0.766	-0.177 (-0.544;0.189)
TG(56:5)	-0.185	0.328	0.766	-0.185 (-0.556;0.186)
Octanoylcarnitine	0.163	0.347	0.77	0.163 (-0.177;0.504)
Lysophosphatidylethanolamine(18:1)	0.156	0.35	0.771	0.156 (-0.171;0.482)
TG(58:9)	-0.162	0.353	0.771	-0.162 (-0.503;0.179)
12,13-EpOME	0.182	0.36	0.782	0.182 (-0.208;0.572)
Sphingomyelin(d18:1/21:0)	-0.17	0.363	0.783	-0.17 (-0.538;0.197)
Hexanoylcarnitine	0.158	0.368	0.789	0.158 (-0.186;0.503)
Taurodeoxycholic acid	-0.146	0.375	0.797	-0.146 (-0.47;0.177)
DGLEA	-0.156	0.381	0.805	-0.156 (-0.506;0.193)
Lysophosphatidylcholine(O-16:0)	-0.162	0.39	0.812	-0.162 (-0.532;0.207)
Phosphatidylcholine(34:3)	-0.154	0.39	0.812	-0.154 (-0.504;0.197)
S-Methylcysteine	-0.153	0.397	0.817	-0.153 (-0.508;0.201)
Citric acid	0.129	0.407	0.817	0.129 (-0.175;0.432)
17,18-DiHETE	0.14	0.4	0.817	0.14 (-0.187;0.467)
Phosphatidylcholine(38:3)	-0.146	0.404	0.817	-0.146 (-0.488;0.197)
Sphingomyelin(d18:1/25:1)	-0.139	0.404	0.817	-0.139 (-0.465;0.187)
TG(56:0)	-0.157	0.408	0.817	-0.157 (-0.531;0.216)
CE(18:2)	-0.15	0.411	0.817	-0.15 (-0.507;0.207)
L-4-hydroxy-proline	0.135	0.424	0.832	0.135 (-0.196;0.465)
L-Glutamine	0.142	0.423	0.832	0.142 (-0.205;0.489)
TG(44:0)	-0.125	0.427	0.832	-0.125 (-0.433;0.183)
Cer(d18:0/23:0)	-0.142	0.441	0.854	-0.142 (-0.503;0.219)
Dodecenoylcarnitine	0.117	0.491	0.858	0.117 (-0.216;0.45)
Hexadecenoylcarnitine	0.121	0.489	0.858	0.121 (-0.221;0.463)
Lauroylcarnitine	0.125	0.469	0.858	0.125 (-0.214;0.465)
Myristoilcarnitine	0.126	0.461	0.858	0.126 (-0.209;0.461)
Trimethylamine N-oxide	0.125	0.486	0.858	0.125 (-0.226;0.477)
3-Hydroxybutyric acid	-0.133	0.462	0.858	-0.133 (-0.488;0.222)
Pyroglutamic acid	-0.125	0.454	0.858	-0.125 (-0.453;0.203)
Lysophosphatidic acid 16(0)	-0.108	0.473	0.858	-0.108 (-0.404;0.187)
Cortisol	-0.122	0.479	0.858	-0.122 (-0.46;0.216)
Cer(d18:1/22:0)	-0.133	0.448	0.858	-0.133 (-0.476;0.211)
DG(36:3)	-0.137	0.452	0.858	-0.137 (-0.494;0.22)
Lysophosphatidylcholine(18:1)	0.129	0.488	0.858	0.129 (-0.236;0.494)

Lysophosphatidylcholine(O-16:1)	-0.135	0.485	0.858	-0.135 (-0.515;0.245)
Lysophosphatidylethanolamine(18:0)	-0.127	0.479	0.858	-0.127 (-0.477;0.224)
Phosphatidylcholine(36:3)	-0.132	0.466	0.858	-0.132 (-0.488;0.224)
Phosphatidylcholine(36:5)	-0.123	0.464	0.858	-0.123 (-0.454;0.207)
TG(58:8)	-0.13	0.469	0.858	-0.13 (-0.482;0.222)
DL-3-aminoisobutyric acid	0.118	0.496	0.861	0.118 (-0.222;0.458)
L-Alanine	-0.111	0.535	0.869	-0.111 (-0.463;0.24)
L-Arginine	-0.113	0.522	0.869	-0.113 (-0.457;0.232)
Symmetric dimethylarginine	0.107	0.518	0.869	0.107 (-0.217;0.431)
Lysophosphatidylinositol 18(2)	0.116	0.515	0.869	0.116 (-0.233;0.464)
Sphingosine 1-phosphate 18(0)	-0.047	0.54	0.869	-0.047 (-0.196;0.102)
Taurolithocholic acid	-0.113	0.531	0.869	-0.113 (-0.467;0.241)
5-HETE	0.103	0.535	0.869	0.103 (-0.223;0.43)
CE(22:6)	-0.104	0.507	0.869	-0.104 (-0.411;0.203)
Phosphatidylcholine(32:1)	0.107	0.539	0.869	0.107 (-0.234;0.448)
Phosphatidylcholine(36:2)	-0.115	0.537	0.869	-0.115 (-0.48;0.25)
Phosphatidylethanolamine(38:6)	-0.101	0.516	0.869	-0.101 (-0.407;0.204)
Sphingomyelin(d18:1/25:0)	-0.113	0.538	0.869	-0.113 (-0.474;0.248)
TG(46:0)	-0.112	0.503	0.869	-0.112 (-0.441;0.217)
TG(56:3)	-0.117	0.53	0.869	-0.117 (-0.481;0.247)
Propionylcarnitine	-0.094	0.547	0.876	-0.094 (-0.398;0.211)
L-Alpha-aminobutyric acid	-0.102	0.556	0.877	-0.102 (-0.442;0.238)
L-Kynurenine	-0.099	0.579	0.877	-0.099 (-0.449;0.251)
L-Leucine	-0.085	0.585	0.877	-0.085 (-0.388;0.219)
L-Serine	-0.089	0.582	0.877	-0.089 (-0.407;0.229)
Lysophosphatidic acid 18(1)	0.092	0.558	0.877	0.092 (-0.216;0.4)
Taurochenodeoxycholic acid	0.095	0.576	0.877	0.095 (-0.238;0.428)
8,12-iPF2a IV	-0.082	0.568	0.877	-0.082 (-0.364;0.2)
8,9-DiHETrE	-0.092	0.564	0.877	-0.092 (-0.403;0.22)
9-HODE	0.089	0.585	0.877	0.089 (-0.231;0.41)
Lysophosphatidylcholine(18:0)	-0.114	0.552	0.877	-0.114 (-0.489;0.261)
Phosphatidylcholine(32:2)	-0.108	0.561	0.877	-0.108 (-0.472;0.256)
Phosphatidylcholine(O-44:5)	0.106	0.579	0.877	0.106 (-0.268;0.48)
TG(56:4)	-0.109	0.567	0.877	-0.109 (-0.484;0.265)
Lysophosphatidylcholine(18:2)	0.101	0.59	0.88	0.101 (-0.266;0.468)
(+/-) 11-HDoHE	-0.056	0.594	0.881	-0.056 (-0.261;0.149)
2-Methylbutyroylcarnitine	-0.081	0.606	0.887	-0.081 (-0.39;0.228)
Butyrylcarnitine	-0.082	0.603	0.887	-0.082 (-0.392;0.228)
Tetradecenoylcarnitine	0.089	0.611	0.887	0.089 (-0.254;0.432)
Lysophosphatidylethanolamine (20:5)	0.084	0.621	0.887	0.084 (-0.249;0.417)
12,13-DiHODE	-0.089	0.618	0.887	-0.089 (-0.437;0.26)
Lysophosphatidylcholine(20:3)	0.089	0.619	0.887	0.089 (-0.261;0.438)
TG(53:1)	-0.089	0.614	0.887	-0.089 (-0.434;0.256)

TG(56:2)	-0.089	0.611	0.887	-0.089 (-0.432;0.254)
L-Histidine	-0.086	0.624	0.887	-0.086 (-0.43;0.258)
Lysophosphatidylethanolamine (16:0)	0.084	0.633	0.887	0.084 (-0.26;0.428)
LPS 18(1)	-0.051	0.628	0.887	-0.051 (-0.259;0.156)
Cholic acid	-0.084	0.636	0.887	-0.084 (-0.431;0.263)
Phosphatidylcholine(34:1)	0.022	0.635	0.887	0.022 (-0.069;0.114)
Cysteine	-0.069	0.661	0.89	-0.069 (-0.377;0.24)
Gamma-L-glutamyl-L-alanine	0.075	0.665	0.89	0.075 (-0.263;0.412)
Taurine	-0.037	0.661	0.89	-0.037 (-0.205;0.13)
L-Tryptophan	-0.074	0.646	0.89	-0.074 (-0.392;0.243)
O-Anandamide	-0.081	0.659	0.89	-0.081 (-0.443;0.28)
Phosphatidylcholine(36:1)	0.078	0.651	0.89	0.078 (-0.259;0.414)
Phosphatidylcholine(40:8)	-0.067	0.653	0.89	-0.067 (-0.358;0.224)
Phosphatidylethanolamine(36:4)	-0.073	0.662	0.89	-0.073 (-0.401;0.255)
Sphingomyelin(d18:1/16:0)	-0.088	0.661	0.89	-0.088 (-0.479;0.304)
FA 18(1)	0.071	0.681	0.908	0.071 (-0.267;0.409)
O-Phosphoethanolamine	-0.027	0.691	0.91	-0.027 (-0.161;0.107)
Lysophosphatidylcholine(14:0)	-0.075	0.691	0.91	-0.075 (-0.445;0.295)
Phosphatidylcholine(O-34:2)	0.071	0.691	0.91	0.071 (-0.28;0.422)
(+/-) 14-HDoHE	-0.027	0.699	0.912	-0.027 (-0.163;0.109)
Lysophosphatidylcholine(20:4)	-0.069	0.697	0.912	-0.069 (-0.419;0.281)
L-Valine	-0.062	0.712	0.918	-0.062 (-0.39;0.267)
Carnitine	0.061	0.712	0.918	0.061 (-0.262;0.384)
Sphingomyelin(d18:1/20:0)	0.07	0.71	0.918	0.07 (-0.297;0.436)
L-Asparagine	-0.06	0.727	0.928	-0.06 (-0.397;0.277)
Pimelylcarnitine	-0.059	0.724	0.928	-0.059 (-0.387;0.269)
Prostaglandin F2α	0.049	0.734	0.934	0.049 (-0.232;0.329)
Sarcosine	0.05	0.745	0.94	0.05 (-0.249;0.349)
FA 18(2)	-0.052	0.742	0.94	-0.052 (-0.365;0.26)
Hydroxylysine	0.051	0.78	0.941	0.051 (-0.308;0.41)
Decenoylcarnitine	0.054	0.761	0.941	0.054 (-0.291;0.399)
Stearoylcarnitine	0.044	0.77	0.941	0.044 (-0.254;0.343)
Lysophosphatidic acid 16(1)	0.042	0.775	0.941	0.042 (-0.246;0.329)
(+/-) 10-HDoHE	-0.028	0.78	0.941	-0.028 (-0.224;0.168)
Anandamide	-0.056	0.759	0.941	-0.056 (-0.413;0.301)
Prostaglandin E2	-0.047	0.772	0.941	-0.047 (-0.364;0.27)
Lysophosphatidylethanolamine(22:6)	-0.054	0.754	0.941	-0.054 (-0.392;0.284)
Sphingomyelin(d18:1/14:0)	-0.056	0.765	0.941	-0.056 (-0.42;0.309)
Sphingomyelin(d18:1/24:1)	-0.056	0.769	0.941	-0.056 (-0.428;0.316)
TG(59:1)	-0.055	0.779	0.941	-0.055 (-0.44;0.33)
Malic acid	-0.044	0.784	0.942	-0.044 (-0.361;0.272)
Linoleylcarnitine	-0.039	0.797	0.947	-0.039 (-0.337;0.259)
Malonylcarnitine	-0.038	0.826	0.947	-0.038 (-0.376;0.3)

Lysophosphatidic acid 18(2)	0.037	0.817	0.947	0.037 (-0.281;0.356)
12(S)-HEPE	-0.016	0.822	0.947	-0.016 (-0.16;0.127)
12-HETE	0.017	0.822	0.947	0.017 (-0.133;0.167)
9,10,13-TriHOME	0.043	0.796	0.947	0.043 (-0.285;0.371)
Palmitoyl ethanolamide	0.041	0.824	0.947	0.041 (-0.318;0.399)
CE(18:1)	-0.045	0.807	0.947	-0.045 (-0.407;0.317)
Lysophosphatidylcholine(16:0)	-0.046	0.805	0.947	-0.046 (-0.412;0.32)
Phosphatidylethanolamine(34:2)	-0.04	0.819	0.947	-0.04 (-0.386;0.306)
Sphingomyelin(d18:1/24:2)	0.044	0.814	0.947	0.044 (-0.327;0.416)
TG(45:0)	-0.046	0.801	0.947	-0.046 (-0.404;0.312)
L-Proline	0.035	0.837	0.949	0.035 (-0.3;0.371)
Oleoylcarnitine	0.036	0.836	0.949	0.036 (-0.308;0.38)
Valerylcarnitine	-0.034	0.838	0.949	-0.034 (-0.357;0.289)
9,12,13-TriHOME	0.034	0.842	0.949	0.034 (-0.301;0.369)
Phosphatidylcholine(O-36:3)	-0.033	0.846	0.949	-0.033 (-0.368;0.302)
TG(54:0)	-0.037	0.846	0.949	-0.037 (-0.414;0.339)
Lactic acid	-0.033	0.851	0.951	-0.033 (-0.375;0.31)
8-HETE	-0.022	0.858	0.953	-0.022 (-0.267;0.223)
Phosphatidylcholine(34:2)	-0.033	0.859	0.953	-0.033 (-0.397;0.331)
Isovalerylcarnitine	-0.026	0.868	0.958	-0.026 (-0.331;0.279)
5,6-DiHETrE	-0.028	0.87	0.958	-0.028 (-0.361;0.306)
L-Isoleucine	0.023	0.886	0.962	0.023 (-0.295;0.341)
Lysophosphatidylinositol 18(0)	-0.023	0.885	0.962	-0.023 (-0.338;0.291)
Sphingosine 1-phosphate 18(1)	0.012	0.88	0.962	0.012 (-0.139;0.162)
Phosphatidylcholine(O-34:3)	-0.027	0.884	0.962	-0.027 (-0.39;0.336)
(+/-) 8-HDoHE	0.026	0.891	0.964	0.026 (-0.341;0.392)
Lysophosphatidylcholine(20:5)	0.019	0.896	0.966	0.019 (-0.273;0.312)
Glycodeoxycholic acid	0.02	0.904	0.972	0.02 (-0.311;0.352)
Lysophosphatidylcholine(22:6)	-0.018	0.914	0.974	-0.018 (-0.348;0.312)
Lysophosphatidylethanolamine(20:4)	0.019	0.92	0.974	0.019 (-0.344;0.381)
Sphingomyelin(d18:1/22:0)	-0.02	0.913	0.974	-0.02 (-0.385;0.344)
Sphingomyelin(d18:1/22:1)	0.019	0.918	0.974	0.019 (-0.352;0.39)
Sphingomyelin(d18:1/16:1)	-0.018	0.924	0.975	-0.018 (-0.395;0.358)
L-Glutamic acid	-0.013	0.934	0.977	-0.013 (-0.325;0.298)
14,15-DiHETrE	-0.014	0.935	0.977	-0.014 (-0.351;0.323)
Phosphatidylcholine(O-34:1)	0.015	0.934	0.977	0.015 (-0.345;0.375)
L-Lysine	-0.005	0.979	0.985	-0.005 (-0.346;0.337)
L-Phenylalanine	0.01	0.954	0.985	0.01 (-0.313;0.332)
Octenoylcarnitine	0.004	0.983	0.985	0.004 (-0.342;0.35)
Tetradecadienylcarnitine	0.009	0.957	0.985	0.009 (-0.335;0.354)
Lysophosphatidylethanolamine (20:4)	-0.007	0.964	0.985	-0.007 (-0.315;0.301)
13-HODE	-0.005	0.974	0.985	-0.005 (-0.289;0.28)
14,15-DiHETE	0.005	0.978	0.985	0.005 (-0.323;0.332)

19,20-DiHDPA	-0.004	0.98	0.985	-0.004 (-0.321;0.313)
Glycolithocholic acid	-0.003	0.985	0.985	-0.003 (-0.344;0.337)
Cer(d18:1/24:1)	0.003	0.985	0.985	0.003 (-0.34;0.347)
Lysophosphatidylcholine(18:3)	-0.007	0.972	0.985	-0.007 (-0.388;0.374)
Phosphatidylcholine(O-38:4)	-0.007	0.968	0.985	-0.007 (-0.366;0.351)
Sphingomyelin(d18:1/24:0)	0.007	0.971	0.985	0.007 (-0.358;0.372)

Shown are the results of the fixed effect meta-analysis: regression coefficient (CI: 95% confidence interval), nominal and adjusted p-values.

Table S6. Cohort-specific results (UCL) for the comparison of all metabolites differently expressed in L-Dopa^{positive} IPD patients compared to healthy controls.

Metabolite	Mean All	Mean HC	Mean L-Dopa^{positive}	Beta	Standard error	Nominal *p*	Adjusted *p*
3-Methoxytyrosine	0.0309	-11.169	0.8422	18.922	0.0524	0	0
Methyldopa	0.1471	-0.8149	0.8272	16.281	0.1409	0	0
N6,N6,N6-Trimethyl-L-lysine	0.0022	-0.3634	0.2607	0.6416	0.206	0.0024	0.2465
Deoxycholic acid	-0.0646	-0.25	0.0652	0.5925	0.2166	0.0074	0.5624
Deoxycarnitine	0.0893	0.2121	0.0033	-0.3449	0.1402	0.0157	0.7931
Glycocholic acid	-0.0389	0.2069	-0.211	-0.5072	0.2022	0.0138	0.7931
Ornithine	0.0994	-0.3796	0.438	0.5273	0.2375	0.0288	0.8743
Glycochenodeoxycholic acid	-0.1165	0.1612	-0.3108	-0.4779	0.2143	0.028	0.8743
Taurocholic acid	-0.0647	0.147	-0.2129	-0.4905	0.2163	0.0255	0.8743
Gamma-L-glutamyl-L-alanine	0.0051	-0.3362	0.2463	0.5425	0.2309	0.0209	0.8743
Sphingomyelin(d18:1/24:0)	0.0369	-0.0171	0.0745	0.3866	0.2576	0.1368	0.9242
Citric acid	-0.0793	-0.4989	0.2144	0.3196	0.2125	0.1357	0.9242
Lysophosphatidylinositol 16(1)	-0.246	-0.3339	-0.1845	0.3538	0.2351	0.1356	0.9242
Phosphatidylcholine(40:7)	0.119	0.2877	0.0016	-0.3496	0.2311	0.1339	0.9242
Thromboxane B2	0.3848	0.6436	0.2036	-0.3161	0.2078	0.1315	0.9242
15(S)-HETrE	0.4589	0.3781	0.5155	0.2587	0.1683	0.1276	0.9242
(+/-) 16-HDoHE	0.1094	-0.1796	0.3117	0.3788	0.2447	0.1248	0.9242
Sphingosine 1-phosphate 18(2)	0.6045	0.5605	0.6353	0.2102	0.1352	0.1234	0.9242
Acetylcarnitine	-0.1304	0.0009	-0.2223	-0.3695	0.2373	0.1226	0.9242
L-Tyrosine	-0.1864	-0.4567	0.0047	0.3633	0.231	0.1192	0.9242
Sphingomyelin(d18:1/20:1)	0.0321	-0.0562	0.0935	0.3816	0.2424	0.119	0.9242
L-Homoserine	0.072	-0.2007	0.2647	0.4523	0.2858	0.1168	0.9242
Phosphatidylcholine(36:1)	-0.248	-0.3859	-0.1519	0.3692	0.2311	0.1136	0.9242
L-Isoleucine	-0.0246	-0.2364	0.1252	0.381	0.2383	0.1132	0.9242
Glycylglycine	0.0584	-0.0491	0.1344	0.3306	0.2056	0.1113	0.9242
8(9)-EpETrE	0.5728	0.4952	0.6272	0.1645	0.1018	0.1091	0.9242
Lysophosphatidylcholine(20:3)	-0.1024	-0.1684	-0.0564	0.3736	0.2308	0.109	0.9242
Lysophosphatidylethanolamine (16:1)	-0.29	-0.4902	-0.15	0.3563	0.2191	0.1072	0.9242
2-Ketoglutaric acid	-0.0698	0.0419	-0.148	-0.3559	0.2159	0.1025	0.9242

L-Threonine	0.1133	-0.0109	0.2011	0.396	0.24	0.1023	0.9242
Lactic acid	0.0939	-0.2473	0.3328	0.3641	0.2171	0.0967	0.9242
Sphingomyelin(d18:1/22:0)	-0.0173	-0.0785	0.0253	0.4402	0.2603	0.0943	0.9242
Betaine	0.1097	0.247	0.0136	-0.4067	0.2307	0.081	0.9242
Methionine sulfone	-0.0436	0.1	-0.1452	-0.4345	0.2389	0.0721	0.9242
Glycine	-0.0101	-0.1644	0.099	0.4136	0.226	0.0704	0.9242
CE(22:6)	0.1433	0.2377	0.0776	-0.4042	0.2207	0.0704	0.9242
Taurodeoxycholic acid	-0.1437	0.0302	-0.2655	-0.4099	0.2228	0.0688	0.9242
Sphingomyelin(d18:1/18:1)	-0.0599	-0.2301	0.0586	0.4218	0.2277	0.0672	0.9242
9,10-DiHOME	0.3911	0.3588	0.4137	0.3862	0.2061	0.0639	0.9242
Lysophosphatidylinositol 20(4)	-0.1761	-0.4239	-0.0027	0.4493	0.2355	0.0594	0.9242
Sphingomyelin(d18:1/20:0)	-0.0842	-0.2646	0.0415	0.5068	0.2539	0.0489	0.9242
Putrescine	0.2372	-0.0051	0.4084	0.3053	0.1509	0.0459	0.9242
Homocysteine	-0.0943	-0.5062	0.1969	0.4759	0.2321	0.0431	0.9242
Glycoursodeoxycholic acid	-0.2266	0.0116	-0.3933	-0.3586	0.172	0.0397	0.9242
Sphingomyelin(d18:1/18:0)	-0.1607	-0.3601	-0.0218	0.505	0.2372	0.036	0.9242
TG(56:4)	-0.2222	-0.3128	-0.1591	0.2081	0.247	0.4017	0.9251
TG(54:3)	-0.2386	-0.3098	-0.189	0.2105	0.2498	0.4016	0.9251
Phosphatidylcholine(36:4)	0.0666	0.0674	0.0659	0.2071	0.2447	0.3995	0.9251
Sphingomyelin(d18:1/16:1)	-0.0001	-0.0564	0.0392	0.2213	0.2592	0.3954	0.9251
Phosphatidylcholine(40:8)	0.1835	0.1783	0.1871	0.168	0.195	0.3913	0.9251
TG(56:2)	-0.2222	-0.3341	-0.1442	0.1711	0.1985	0.3909	0.9251
5,6-DiHETrE	0.008	-0.0781	0.0682	0.1842	0.2122	0.3874	0.9251
Citrulline	-0.1499	-0.1097	-0.1783	-0.2136	0.2425	0.3807	0.9251
Decanoylcarnitine	-0.1271	-0.0733	-0.1647	-0.2057	0.2333	0.3803	0.9251
Palmitoyl ethanolamide	-0.1439	-0.2616	-0.0615	0.2262	0.2557	0.3787	0.9251
Phosphatidylcholine(38:6)	0.1105	0.1442	0.0871	-0.2017	0.226	0.3744	0.9251
Lysophosphatidylcholine(18:1)	-0.0345	0.0193	-0.072	0.2149	0.2407	0.3743	0.9251
TG(52:2)	-0.3101	-0.4343	-0.2236	0.2218	0.2473	0.3722	0.9251
Phosphatidylcholine(32:2)	-0.0872	-0.1765	-0.0251	0.2333	0.2595	0.371	0.9251
Lysophosphatidylcholine(O-16:0)	0.0911	0.2757	-0.0375	-0.21	0.2328	0.3695	0.9251

Prostaglandin F2α	0.2761	0.385	0.1998	-0.2218	0.2454	0.3682	0.9251
TG(50:3)	-0.2075	-0.3298	-0.1223	0.222	0.2428	0.3631	0.9251
Methylmalonylcarnitine	0.2469	0.3733	0.1585	-0.1854	0.202	0.3612	0.9251
TG(60:1)	-0.1006	0.0479	-0.204	-0.2136	0.2312	0.3578	0.9251
Glycolithocholic acid	-0.0156	-0.0264	-0.008	0.2035	0.2179	0.3528	0.9251
L-Valine	0.0375	-0.0762	0.1178	0.2159	0.2309	0.3521	0.9251
3-Hydroxypropionic Acid	0.0594	-0.0559	0.1402	-0.2647	0.2826	0.3512	0.9251
Lysophosphatidylcholine(14:0)	-0.12	-0.2699	-0.0156	0.243	0.259	0.3506	0.9251
8,12-iPF2a IV	0.0152	0.0316	0.0037	0.1978	0.2103	0.3492	0.9251
19,20-DiHDPA	0.1426	0.1806	0.1159	-0.2382	0.2528	0.3484	0.9251
Phosphatidylcholine(O-38:4)	-0.2603	-0.3528	-0.1959	0.2389	0.2533	0.348	0.9251
TG(53:1)	-0.2504	-0.3895	-0.1535	0.1923	0.2037	0.3477	0.9251
L-Serine	-0.0179	0.2479	-0.2058	-0.2223	0.2351	0.3467	0.9251
FA 22(6)	0.1894	0.2147	0.1716	-0.2117	0.2238	0.3466	0.9251
TG(56:6)	-0.0924	-0.1874	-0.0262	0.2503	0.2628	0.3433	0.9251
Isobutyrylcarnitine	0.0767	0.0187	0.1172	-0.2068	0.2138	0.3357	0.9251
Lysophosphatidylcholine(20:4)	0.1241	0.1591	0.0997	0.2322	0.233	0.3217	0.9251
Lysophosphatidic acid 16(0)	-0.1689	-0.0354	-0.2624	-0.1948	0.1943	0.3185	0.9251
Lysophosphatidylethanolamine(20:4)	-0.0646	-0.0895	-0.0473	0.2309	0.2299	0.3178	0.9251
L-Proline	-0.0135	-0.1722	0.0987	0.2225	0.2187	0.3116	0.9251
TG(56:1)	-0.1066	-0.1092	-0.1048	-0.185	0.1812	0.3101	0.9251
14,15-DiHETE	0.1379	0.1991	0.0951	-0.2548	0.2488	0.3084	0.9251
TG(50:2)	-0.3052	-0.4554	-0.2005	0.2418	0.2352	0.3066	0.9251
TG(54:2)	-0.3215	-0.4599	-0.2251	0.2431	0.2362	0.306	0.9251
Phosphatidylcholine(36:3)	-0.1375	-0.1484	-0.1299	0.2675	0.2599	0.306	0.9251
Gamma-Glutamylglutamine	-0.0005	-0.1014	0.0709	0.2434	0.2356	0.3041	0.9251
L-Tryptophan	-0.0403	-0.027	-0.0498	0.2329	0.2252	0.3037	0.9251
Lysophosphatidylinositol 18(1)	-0.2189	-0.3481	-0.1285	0.2527	0.2441	0.3031	0.9251
3-Hydroxybutyric acid	0.021	0.1334	-0.0577	-0.2549	0.2462	0.3029	0.9251
Lysophosphatidylcholine(O-18:1)	0.3489	0.5472	0.2108	-0.2249	0.2167	0.3022	0.9251
Phosphatidylcholine(O-38:6)	0.0227	0.1374	-0.0572	-0.2625	0.2493	0.2952	0.9251

TG(42:0)	-0.0754	-0.0144	-0.1179	-0.1889	0.1792	0.2944	0.9251
8-HETE	0.4854	0.4673	0.498	0.1788	0.1672	0.2877	0.9251
Prostaglandin E2	0.4122	0.5827	0.2928	-0.2309	0.2155	0.2866	0.9251
12,13-DiHOME	0.1276	0.172	0.0964	0.2414	0.2245	0.2849	0.9251
12,13-EpOME	0.0191	-0.0484	0.0664	0.2645	0.2455	0.284	0.9251
Lysophosphatidylethanolamine (20:5)	0.0444	0.0236	0.059	-0.2574	0.2381	0.2825	0.9251
Dehydroepiandrosterone	-0.0045	0.0405	-0.036	-0.2422	0.2223	0.2787	0.9251
TG(56:0)	0.0431	0.1093	-0.0029	-0.2421	0.2217	0.2776	0.9251
1-Methylhistidine	-0.1092	-0.0813	-0.1289	-0.2449	0.2205	0.2695	0.9251
5-HETE	0.0555	-0.0043	0.0973	0.256	0.2224	0.2526	0.9251
Ethanolamine	-0.1058	-0.0116	-0.1724	0.254	0.2203	0.2519	0.9251
S-Methylcysteine	0.2226	0.3429	0.1376	-0.2994	0.2594	0.2514	0.9251
L-Leucine	0.0195	-0.0964	0.1015	0.256	0.2199	0.2472	0.9251
Sphingomyelin(d18:1/15:0)	-0.1779	-0.183	-0.1743	-0.2816	0.2412	0.246	0.9251
Cer(d18:1/23:0)	-0.1842	-0.0651	-0.2672	-0.2844	0.2427	0.2444	0.9251
TG(57:1)	-0.0022	0.0976	-0.0717	-0.273	0.2314	0.2411	0.9251
Tiglylcarnitine	0.1416	0.2026	0.0989	-0.2845	0.2401	0.2388	0.9251
Sphingomyelin(d18:1/23:1)	-0.06	0.0408	-0.1303	-0.2839	0.2379	0.2359	0.9251
TG(54:0)	0.0458	-0.0994	0.1468	0.3036	0.2527	0.2327	0.9251
Serotonine	0.5697	0.6624	0.5041	-0.1519	0.1241	0.224	0.9251
Sphingomyelin(d18:1/22:1)	-0.009	-0.0776	0.0388	0.3135	0.2559	0.2237	0.9251
Pimelylcarnitine	0.0591	0.2571	-0.0794	-0.2796	0.2271	0.2213	0.9251
Lysophosphatidylinositol 18(2)	0.0356	0.0484	0.0267	0.3199	0.2513	0.206	0.9251
Saccharopine	0.0314	-0.112	0.1327	0.3078	0.2414	0.2054	0.9251
Gamma-aminobutyric acid	0.0579	0.1742	-0.0242	-0.2191	0.1713	0.204	0.9251
TG(56:3)	-0.276	-0.4295	-0.1691	0.3026	0.2347	0.2005	0.9251
Glycodeoxycholic acid	-0.0874	-0.0902	-0.0853	0.292	0.2244	0.1963	0.9251
Phosphatidylcholine(32:1)	-0.2907	-0.4707	-0.1653	0.2969	0.2275	0.1952	0.9251
L-Alanine	0.0027	-0.2539	0.1841	0.3029	0.2309	0.1926	0.9251
9-HOTrE	0.1577	0.1605	0.1557	0.2981	0.2265	0.1912	0.9251
Phosphatidylcholine(34:1)	0.005	-0.0258	0.0265	0.0837	0.0632	0.1891	0.9251

Cer(d18:1/22:0)	-0.1202	0.1051	-0.2771	-0.4195	0.3156	0.1871	0.9251
Glutathione	0.3128	0.546	0.1479	-0.226	0.1701	0.1871	0.9251
Homocitrulline	-0.0503	-0.0316	-0.0635	-0.3298	0.2426	0.1771	0.9251
O-Acetyl-L-serine	0.1606	0.0505	0.2385	-0.3153	0.2311	0.1757	0.9251
Cer(d18:0/22:0)	-0.1285	0.0288	-0.2381	-0.3376	0.2401	0.1632	0.9251
TG(56:5)	-0.1459	-0.3072	-0.0336	0.3628	0.2562	0.1601	0.9251
Phosphatidylcholine(38:3)	-0.253	-0.4027	-0.1487	0.3384	0.2386	0.1595	0.9251
Cystathionine	-0.146	-0.4798	0.0899	0.251	0.1753	0.1554	0.9251
Lysophosphatidylcholine(16:1)	-0.1928	-0.26	-0.1461	0.3385	0.2328	0.1493	0.9251
Phosphatidylethanolamine(36:4)	-0.1801	-0.3052	-0.093	0.3601	0.2456	0.1461	0.9251
Lysophosphatidic acid 14(0)	-0.2558	-0.2808	-0.2384	-0.1703	0.2037	0.4053	0.9264
L-Glutamic acid	0.2497	-0.0163	0.4378	0.1564	0.193	0.4197	0.9314
TG(52:3)	-0.1342	-0.1805	-0.102	0.2088	0.2565	0.4177	0.9314
Phosphatidylcholine(O-44:5)	0.0675	-0.0457	0.1464	0.2146	0.2621	0.4151	0.9314
9-HODE	0.3243	0.3703	0.2922	0.1793	0.2179	0.4125	0.9314
TG(52:1)	-0.2857	-0.4291	-0.1858	0.1946	0.244	0.4271	0.934
Phosphatidylethanolamine(34:2)	-0.1916	-0.1973	-0.1876	0.1982	0.2476	0.4255	0.934
Propionylcarnitine	-0.08	-0.2893	0.0666	0.1266	0.2053	0.5387	0.9412
12-HETE	0.6894	0.6984	0.6831	0.0517	0.0836	0.538	0.9412
Decenoylcarnitine	0.1133	0.2103	0.0454	-0.1563	0.2527	0.5377	0.9412
TG(52:4)	-0.0023	0.0161	-0.015	0.1583	0.255	0.5363	0.9412
Phosphatidylcholine(38:7)	0.0531	0.0596	0.0486	0.1536	0.2454	0.5329	0.9412
TG(55:1)	-0.1499	-0.1025	-0.1829	-0.1561	0.2463	0.5278	0.9412
Isovalerylcarnitine	0.0434	-0.0497	0.1085	0.1347	0.2096	0.5219	0.9412
Lysophosphatidylcholine(18:2)	0.0809	0.2391	-0.0293	0.161	0.2493	0.52	0.9412
Nonaylcarnitine	-0.0193	-0.0708	0.0168	-0.1405	0.2171	0.5191	0.9412
12,13-DiHODE	0.0719	0.2112	-0.0256	-0.1553	0.2372	0.5143	0.9412
Lysophosphatidylcholine(18:3)	-0.0991	-0.169	-0.0504	0.2099	0.3204	0.5141	0.9412
Lysophosphatidic acid 18(2)	-0.1169	0.1319	-0.2911	-0.1515	0.2275	0.507	0.9412
Phosphatidylethanolamine(38:6)	-0.0144	-0.0846	0.0345	0.1554	0.2326	0.5058	0.9412
O-Anandamide	-0.1835	-0.2055	-0.1682	0.1667	0.2478	0.5027	0.9412

Lysophosphatidylethanolamine (20:4)	-0.1518	-0.2152	-0.1074	0.1466	0.2171	0.5011	0.9412
17,18-DiHETE	0.0613	0.0015	0.1032	-0.1562	0.2305	0.4996	0.9412
Phosphatidylethanolamine(38:4)	-0.0883	-0.2083	-0.0047	0.1498	0.2175	0.4925	0.9412
Phosphatidylcholine(40:5)	-0.071	-0.1362	-0.0256	0.1822	0.2621	0.4887	0.9412
Lauroylcarnitine	-0.1611	-0.1005	-0.2036	-0.1546	0.2219	0.4877	0.9412
Cholic acid	-0.0347	-0.0811	-0.0022	-0.165	0.2352	0.4845	0.9412
TG(54:5)	0.0043	0.0144	-0.0028	0.1776	0.2523	0.4832	0.9412
CE(18:2)	0.0444	0.2209	-0.0785	-0.1826	0.2583	0.4815	0.9412
Phosphatidylcholine(O-38:5)	-0.0289	0.1557	-0.1574	-0.1882	0.266	0.481	0.9412
TG(50:1)	-0.2831	-0.3989	-0.2024	0.1696	0.2373	0.4768	0.9412
8,9-DiHETrE	-0.035	-0.0691	-0.0112	0.1742	0.2433	0.4759	0.9412
13-HODE	0.4321	0.4992	0.3852	0.1403	0.1932	0.4694	0.9412
Sphingomyelin(d18:1/18:2)	0.0312	0.0825	-0.0046	-0.1975	0.2695	0.4654	0.9412
TG(48:3)	-0.172	-0.1372	-0.1963	-0.1549	0.2083	0.459	0.9412
TG(54:4)	-0.0397	-0.0458	-0.0355	0.1924	0.2577	0.4571	0.9412
L-4-hydroxy-proline	-0.1413	-0.2971	-0.0311	0.159	0.2129	0.457	0.9412
DG(36:2)	-0.0704	-0.0337	-0.0959	-0.1571	0.2056	0.4468	0.9412
Lysophosphatidylethanolamine(18:1)	-0.0379	-0.0646	-0.0193	0.1435	0.1868	0.4443	0.9412
TG(42:1)	-0.0643	0.0163	-0.1205	-0.1493	0.1936	0.4426	0.9412
Taurochenodeoxycholic acid	-0.1376	-0.1333	-0.1407	0.1765	0.2274	0.4396	0.9412
Octanoylcarnitine	-0.099	-0.0688	-0.1202	-0.1903	0.2422	0.4338	0.9412
2-Methylbutyroylcarnitine	0.0299	-0.1854	0.1806	0.1359	0.2228	0.5433	0.9437
Sphingomyelin(d18:1/14:0)	-0.1338	-0.2964	-0.0206	0.1502	0.2526	0.5535	0.9454
Phosphatidylcholine(34:4)	-0.0657	-0.1679	0.0054	0.1442	0.2419	0.5526	0.9454
TG(58:2)	-0.0793	-0.0126	-0.1257	-0.1509	0.2513	0.5495	0.9454
L-Phenylalanine	0.0028	-0.0258	0.0231	0.1346	0.2315	0.5625	0.95
Lysophosphatidylcholine(16:0)	-0.0035	0.1082	-0.0814	0.1445	0.2468	0.5596	0.95
Anandamide	-0.2039	-0.2176	-0.1943	0.1364	0.2434	0.5765	0.9524
Phosphatidylethanolamine(O-38:7)	0.0369	0.0879	0.0015	-0.1355	0.241	0.5753	0.9524
FA 18(1)	-0.0621	-0.1729	0.0155	0.13	0.2286	0.571	0.9524
Phosphatidylcholine(38:4)	0.0506	0.0476	0.0526	0.1457	0.2553	0.5697	0.9524

Sphingosine 1-phosphate 18(0)	0.6904	0.6951	0.6871	-0.0442	0.0826	0.5936	0.9547
Sphingomyelin(d18:1/25:0)	-0.0936	-0.0224	-0.1432	-0.1202	0.2232	0.5916	0.9547
Phosphatidylcholine(36:2)	-0.1002	0.0133	-0.1792	0.1487	0.2753	0.5906	0.9547
14,15-DiHETrE	-0.0208	-0.0095	-0.0288	0.1323	0.2448	0.59	0.9547
Tetradecadienylcarnitine	0.1049	0.2475	0.005	-0.1221	0.2235	0.5862	0.9547
Phosphatidylcholine(O-36:4)	-0.1441	0.0484	-0.2782	-0.1357	0.2572	0.599	0.9585
Sphingomyelin(d18:1/23:0)	-0.1225	-0.0163	-0.1964	-0.1337	0.256	0.6027	0.9593
L-Lysine	0.0486	-0.0559	0.1225	0.1279	0.2609	0.6251	0.9598
Malic acid	0.2395	0.0654	0.3613	0.1009	0.2058	0.6251	0.9598
Phosphatidylcholine(40:6)	0.0406	-0.0241	0.0857	-0.1113	0.2249	0.6219	0.9598
TG(54:1)	-0.2146	-0.2576	-0.1846	-0.1257	0.2533	0.6209	0.9598
TG(42:2)	-0.0286	0.0153	-0.0592	-0.1047	0.2071	0.6142	0.9598
Tetradecenoylcarnitine	-0.127	-0.0668	-0.1691	-0.1106	0.2185	0.6138	0.9598
Phosphatidylcholine(O-34:2)	-0.1394	-0.1581	-0.1264	0.1688	0.3292	0.6094	0.9598
Phosphatidylethanolamine(O-36:5)	0.1142	0.2675	0.0075	-0.1151	0.2393	0.6318	0.9651
Phosphatidylcholine(36:5)	0.0644	-0.0225	0.1249	-0.1052	0.2221	0.6369	0.9681
TG(46:2)	-0.1745	-0.1792	-0.1713	-0.0852	0.1872	0.6502	0.9785
Palmitoylcarnitine	-0.1274	-0.2958	-0.0096	0.0975	0.2134	0.6487	0.9785
Sphingomyelin(d18:1/24:2)	0.059	0.105	0.027	0.1035	0.2499	0.6796	0.9791
LPS 18(1)	0.6504	0.7011	0.615	-0.0505	0.1216	0.6792	0.9791
Phosphatidylcholine(34:2)	-0.0546	0.0534	-0.1298	0.1107	0.266	0.6783	0.9791
Dodecenoylcarnitine	-0.1314	-0.0884	-0.1615	-0.0927	0.2223	0.6777	0.9791
Linoleoyl ethanolamide	0.1047	0.1722	0.0574	0.0969	0.2287	0.6726	0.9791
TG(51:1)	-0.3146	-0.4604	-0.2131	0.0905	0.2095	0.6667	0.9791
Malonylcarnitine	-0.0363	0.0281	-0.0814	-0.1218	0.2766	0.6606	0.9791
CE(18:1)	-0.1569	-0.1408	-0.1681	0.1143	0.2581	0.6589	0.9791
Trimethylamine N-oxide	-0.0711	-0.3266	0.1077	0.1114	0.2503	0.6573	0.9791
(+/-) 8-HDoHE	0.0582	-0.0687	0.1471	0.1019	0.2532	0.6881	0.982
TG(48:2)	-0.2508	-0.3452	-0.185	0.0898	0.2218	0.6865	0.982
Sphingosine 1-phosphate 18(1)	0.6906	0.6918	0.6898	0.0137	0.0836	0.8703	0.9872
Symmetric dimethylarginine	-0.0246	-0.1061	0.033	0.038	0.2292	0.8685	0.9872

TG(44:1)	-0.1393	-0.1571	-0.1269	-0.031	0.1834	0.8662	0.9872
L-Arginine	-0.2671	-0.3054	-0.24	-0.0407	0.238	0.8647	0.9872
Phosphatidylcholine(O-34:3)	0.0946	0.2315	-0.0008	0.0451	0.2552	0.86	0.9872
TG(51:2)	-0.3256	-0.4583	-0.2331	0.0402	0.2254	0.8588	0.9872
Lysophosphatidic acid 16(1)	-0.3048	-0.2937	-0.3126	-0.0363	0.2007	0.8567	0.9872
TG(44:0)	-0.0898	-0.1069	-0.0779	-0.0317	0.1738	0.8558	0.9872
1-AG/2-AG	-0.0825	-0.1394	-0.0427	-0.0428	0.2318	0.8539	0.9872
TG(45:0)	-0.043	-0.0854	-0.0135	0.0389	0.2086	0.8524	0.9872
9,10,13-TriHOME	-0.3355	-0.4923	-0.2258	0.0465	0.2478	0.8514	0.9872
Lysophosphatidylcholine(20:5)	0.1485	0.1057	0.1784	-0.0369	0.194	0.8495	0.9872
O-Phosphoethanolamine	0.6632	0.696	0.64	-0.0138	0.072	0.8486	0.9872
Sphingomyelin(d18:1/21:0)	-0.1292	-0.1434	-0.1193	-0.0475	0.2446	0.8464	0.9872
Phosphatidylcholine(O-34:1)	-0.0854	-0.0069	-0.1401	-0.0497	0.2485	0.8419	0.9872
TG(60:3)	-0.0803	-0.1039	-0.0638	0.0464	0.2317	0.8418	0.9872
TG(46:0)	-0.102	-0.1652	-0.058	0.0395	0.192	0.8376	0.9872
Lysophosphatidylcholine(22:6)	0.1531	0.188	0.1288	-0.0498	0.2369	0.8339	0.9872
Sphingomyelin(d18:1/25:1)	-0.1376	-0.0804	-0.1773	-0.0513	0.2305	0.8244	0.9872
11,12-DiHETrE	0.0038	0.1316	-0.0857	-0.0571	0.2499	0.8199	0.9872
Cer(d18:1/24:1)	-0.1312	-0.0443	-0.1917	0.0496	0.2162	0.8191	0.9872
Hydroxylysine	-0.1407	-0.3013	-0.0271	0.0558	0.2364	0.8138	0.9872
(+/-) 14-HDoHE	0.6853	0.6896	0.6823	-0.0175	0.074	0.8137	0.9872
Linoleylcarnitine	0.3991	0.4735	0.347	-0.0455	0.192	0.8133	0.9872
Phosphatidylethanolamine(O-38:5)	0.043	0.1352	-0.0213	-0.0579	0.2433	0.8126	0.9872
Butyrylcarnitine	-0.1569	-0.1662	-0.1505	-0.0545	0.2283	0.8118	0.9872
Phosphatidylcholine(O-36:5)	0.0126	0.1342	-0.0721	-0.0668	0.27	0.8051	0.9872
TG(58:9)	0.2166	0.1438	0.2673	-0.0643	0.2462	0.7945	0.9872
Phosphatidylcholine(38:2)	-0.2275	-0.206	-0.2424	0.0687	0.2547	0.7878	0.9872
TG(51:3)	-0.179	-0.2694	-0.116	0.0611	0.2218	0.7836	0.9872
Lysophosphatidylethanolamine(18:0)	-0.1069	-0.1062	-0.1074	-0.066	0.2375	0.7818	0.9872
Octenoylcarnitine	0.0131	0.1545	-0.0858	-0.0676	0.2392	0.778	0.9872
L-Glutamine	-0.042	-0.1433	0.0295	0.0724	0.2509	0.7734	0.9872

Pyroglutamic acid	0.0512	0.0417	0.0578	-0.0581	0.2009	0.773	0.9872
CE(20:4)	0.0266	0.0731	-0.0058	-0.0722	0.2491	0.7726	0.9872
(+/-) 10-HDoHE	0.6583	0.6632	0.655	-0.0349	0.1193	0.7706	0.9872
L-2-aminoadipic acid	0.0991	-0.0948	0.2362	0.0669	0.2194	0.7612	0.9872
1-LG/2-LG	-0.1297	-0.1523	-0.1139	0.0709	0.2288	0.7574	0.9872
Phosphatidylethanolamine(38:2)	-0.0998	-0.0754	-0.1167	-0.0746	0.238	0.7548	0.9872
Lysophosphatidic acid 18(1)	-0.1915	-0.0876	-0.2643	-0.0682	0.211	0.7471	0.9872
TG(50:4)	-0.0951	-0.1188	-0.0786	0.0765	0.2364	0.7471	0.9872
Hexanoylcarnitine	-0.1553	-0.2185	-0.1111	-0.0769	0.2364	0.7456	0.9872
DG(36:3)	-0.0026	0.0165	-0.0159	-0.07	0.2104	0.74	0.9872
Sphingomyelin(d18:1/24:1)	-0.0097	0.0997	-0.0859	0.0872	0.2614	0.7395	0.9872
Phosphatidylcholine(34:3)	-0.1627	-0.1157	-0.1955	0.0803	0.2371	0.7355	0.9872
11-HETE	0.3397	0.3533	0.3302	0.0769	0.2257	0.734	0.9872
Phosphatidylcholine(38:5)	0.0541	0.11	0.0151	-0.0787	0.2303	0.7333	0.9872
Lysophosphatidylcholine(18:0)	-0.0094	0.1097	-0.0924	0.089	0.2574	0.7302	0.9872
Valerylcarnitine	-0.1459	-0.269	-0.0598	-0.079	0.2239	0.7252	0.9872
Choline	0.0183	-0.1258	0.1192	-0.0845	0.2365	0.7216	0.9872
L-Kynurenine	-0.1618	-0.2276	-0.1154	-0.0823	0.2281	0.7191	0.9872
Oleoylcarnitine	-0.0459	-0.0752	-0.0254	-0.0787	0.2181	0.7188	0.9872
TG(48:1)	-0.2803	-0.3934	-0.2015	0.0824	0.2253	0.7154	0.9872
TG(44:2)	-0.0921	-0.0763	-0.1031	-0.0703	0.1891	0.7108	0.9872
Cortisol	-0.2443	-0.1709	-0.2956	0.097	0.2556	0.7051	0.9872
TG(46:1)	-0.2307	-0.2895	-0.1897	0.0293	0.1998	0.8837	0.9877
Cysteine	-0.2264	-0.5047	-0.0297	-0.0327	0.2223	0.8833	0.9877
TG(60:2)	-0.1163	-0.1073	-0.1226	-0.0367	0.2423	0.8799	0.9877
Phosphatidylcholine(O-36:3)	-0.0362	-0.0883	0.0001	0.0428	0.2782	0.8781	0.9877
Lysophosphatidylcholine(O-16:1)	0.1063	0.2635	-0.0032	-0.0313	0.2511	0.9011	0.9925
L-Alpha-aminobutyric acid	0.1426	0.164	0.1275	-0.0301	0.2364	0.8988	0.9925
Sphingomyelin(d18:1/16:0)	0.0423	0.1423	-0.0275	0.0349	0.2694	0.8971	0.9925
2-Hydroxybutyric acid	0.1591	0.1031	0.1982	0.0337	0.2491	0.8926	0.9925
(+/-) 11-HDoHE	0.6408	0.6238	0.6527	-0.0011	0.1174	0.9924	0.9957

L-Histidine	0.0289	0.1627	-0.0656	-0.0024	0.2422	0.9921	0.9957
Phosphatidylcholine(32:0)	-0.041	-0.0267	-0.0509	-0.0027	0.2403	0.9912	0.9957
Hexadecenoylcarnitine	-0.1857	-0.2891	-0.1134	-0.0038	0.2106	0.9855	0.9957
Carnitine	-0.1224	-0.2609	-0.0254	0.0044	0.2157	0.9837	0.9957
FA 18(2)	0.4044	0.4658	0.3615	0.0044	0.2061	0.9832	0.9957
TG(58:8)	0.0635	-0.0812	0.1642	0.0055	0.2536	0.9829	0.9957
L-Asparagine	0.1631	0.255	0.0981	-0.007	0.2386	0.9765	0.9957
Myristoilcarnitine	-0.2238	-0.3531	-0.1333	-0.0068	0.2074	0.974	0.9957
Lysophosphatidylinositol 18(0)	-0.2656	-0.4671	-0.1246	0.0075	0.22	0.9728	0.9957
Sarcosine	-0.355	-0.3564	-0.3541	0.0069	0.1916	0.9715	0.9957
Stearoylcarnitine	-0.1348	-0.2406	-0.0607	-0.0072	0.1798	0.9682	0.9957
FA 22(5) w6	0.0784	0.1632	0.019	-0.0098	0.2316	0.9664	0.9957
DL-3-aminoisobutyric acid	-0.1381	-0.1606	-0.1221	0.01	0.2284	0.9651	0.9957
12(S)-HEPE	0.6821	0.6831	0.6814	-0.0037	0.0786	0.9628	0.9957
Lysophosphatidylethanolamine(22:6)	0.0583	-0.0057	0.1029	-0.0106	0.2266	0.9627	0.9957
Lysophosphatidylethanolamine (16:0)	0.0341	0.0399	0.03	0.0118	0.2451	0.9616	0.9957
Isocitrate	0.4208	0.2294	0.5547	-0.009	0.1788	0.96	0.9957
Taurine	0.6374	0.6652	0.6177	-0.0055	0.0945	0.9533	0.9957
DGLEA	-0.2119	-0.1844	-0.2311	0.0153	0.2423	0.9499	0.9957
TG(51:4)	-0.1107	-0.1818	-0.0612	0.0163	0.2288	0.9432	0.9957
TG(54:7)	0.1806	0.1531	0.1997	-0.0192	0.2588	0.9409	0.9957
Taurolithocholic acid	-0.0226	0.0309	-0.0601	0.0179	0.2375	0.9401	0.9957
TG(56:7)	0.0608	0.0119	0.0949	-0.0208	0.2548	0.935	0.9957
L-Methionine	0.0227	0.0649	-0.0071	-0.019	0.211	0.9284	0.9957
9,12,13-TriHOME	-0.2889	-0.3845	-0.222	0.0258	0.2378	0.9138	0.9957
Cer(d18:0/23:0)	-0.1058	-0.0823	-0.1222	-0.0272	0.233	0.9074	0.9957
TG(59:1)	-0.1564	-0.139	-0.1686	-0.0005	0.2945	0.9987	0.9987

Table S7. Cohort-specific results (UL) for the comparison of all metabolites differently expressed in L-Dopa^{positive} IPD patients compared to healthy controls.

Metabolite	Mean All	Mean HC	Mean L-Dopa^{positive}	Beta	Standard error	Nominal *p*	Adjusted *p*
3-Methoxytyrosine	0.2878	-1.076	0.9001	20.205	0.0581	0	0
Methyldopa	0.0266	-0.8807	0.434	14.321	0.2211	0	0
(+/-) 16-HDoHE	0.0609	-0.7874	0.4341	1.218	0.2389	0	0.0003
Putrescine	-0.3271	-10.056	-0.0224	0.9844	0.2032	0	0.0006
Phosphatidylcholine(38:4)	-0.059	0.7363	-0.3572	-0.9828	0.276	0.0007	0.0436
Ornithine	-0.1877	-0.6056	-0.0001	0.6153	0.1843	0.0014	0.0483
TG(50:3)	0.211	0.8925	-0.0445	-0.9191	0.2753	0.0014	0.0483
TG(50:4)	0.1328	0.836	-0.131	-0.9643	0.2823	0.0011	0.0483
TG(54:7)	-0.1469	0.5119	-0.394	-0.8648	0.2565	0.0013	0.0483
TG(54:5)	0.0595	0.7138	-0.1859	-0.9365	0.2836	0.0016	0.0485
Phosphatidylcholine(38:7)	-0.084	0.6776	-0.3696	-0.9301	0.29	0.0021	0.0542
TG(56:6)	0.1543	0.8093	-0.0913	-0.8475	0.2645	0.0021	0.0542
TG(51:4)	0.16	0.8919	-0.1144	-10.086	0.3178	0.0023	0.0548
2-Hydroxybutyric acid	-0.2958	0.1759	-0.5034	-0.7751	0.2549	0.0034	0.0577
Phosphatidylcholine(36:4)	-0.0444	0.6824	-0.3169	-0.9012	0.2886	0.0027	0.0577
TG(48:2)	0.2904	0.9137	0.0567	-0.8448	0.2815	0.0039	0.0577
TG(50:2)	0.3215	0.9105	0.1007	-0.7837	0.26	0.0037	0.0577
TG(51:1)	0.4619	11.636	0.1987	-0.9311	0.3113	0.004	0.0577
TG(51:3)	0.291	10.272	0.0149	-0.9634	0.3195	0.0037	0.0577
TG(52:4)	0.0274	0.6582	-0.2092	-0.9048	0.2918	0.0029	0.0577
TG(56:5)	0.2097	0.8568	-0.033	-0.8403	0.2802	0.0039	0.0577
Phosphatidylcholine(40:5)	-0.0489	0.5397	-0.2697	-0.6795	0.2309	0.0046	0.0586
TG(51:2)	0.4308	11.171	0.1735	-0.8516	0.2898	0.0046	0.0586
TG(52:1)	0.4306	0.947	0.2369	-0.7452	0.2521	0.0044	0.0586
TG(48:1)	0.3489	0.915	0.1367	-0.7969	0.2757	0.0053	0.0644
TG(54:2)	0.4932	10.167	0.2969	-0.763	0.2652	0.0055	0.0644
TG(52:3)	0.1817	0.7478	-0.0306	-0.8083	0.2829	0.0058	0.0655
TG(50:1)	0.3487	0.8682	0.1539	-0.743	0.2629	0.0063	0.0687

TG(60:3)	0.1772	0.8135	-0.0614	-0.9055	0.3221	0.0066	0.0691
Phosphatidylcholine(34:4)	-0.0129	0.6405	-0.2579	-0.7711	0.2761	0.007	0.0704
Phosphatidylethanolamine(38:4)	0.2359	0.9716	-0.04	-0.7785	0.2806	0.0073	0.0716
Phosphatidylcholine(38:3)	0.1389	0.7052	-0.0734	-0.7056	0.2565	0.0078	0.074
TG(54:1)	0.4147	0.7873	0.2749	-0.5741	0.211	0.0085	0.0779
TG(56:3)	0.4539	10.222	0.2408	-0.8195	0.3038	0.009	0.0804
TG(56:2)	0.3195	10.288	0.0535	-0.9929	0.37	0.0093	0.0811
L-Alanine	-0.1957	0.3062	-0.421	-0.7408	0.2846	0.0114	0.0893
Methylmalonylcarnitine	-0.2026	0.1787	-0.3703	-0.6245	0.2403	0.0115	0.0893
TG(52:2)	0.369	0.8618	0.1842	-0.6693	0.2546	0.0108	0.0893
TG(53:1)	0.4145	11.136	0.1524	-0.9142	0.3491	0.0111	0.0893
Lactic acid	-0.1099	0.3918	-0.3306	-0.7616	0.2942	0.0118	0.0894
TG(54:4)	0.1498	0.6451	-0.0359	-0.7249	0.2918	0.0157	0.1165
Phosphatidylethanolamine(O-38:5)	0.0982	0.6632	-0.1137	-0.7698	0.3112	0.0161	0.1168
L-Threonine	-0.2033	-0.6475	-0.0038	0.5702	0.2329	0.017	0.1201
TG(54:3)	0.3676	0.8501	0.1867	-0.6718	0.282	0.0203	0.14
Decanoylcarnitine	0.2303	-0.031	0.3453	0.5877	0.2488	0.0211	0.1423
TG(48:3)	0.1645	0.7034	-0.0376	-0.736	0.3165	0.0234	0.1543
1-AG/2-AG	0.2478	0.6343	0.0778	-0.5827	0.2546	0.0252	0.1564
CE(20:4)	-0.0591	0.5692	-0.2948	-0.6773	0.2937	0.0245	0.1564
FA 22(5) w6	-0.3243	0.1482	-0.5323	-0.5852	0.2565	0.0256	0.1564
L-2-aminoadipic acid	-0.0669	0.1531	-0.1656	-0.5634	0.2478	0.0262	0.1564
Phosphatidylcholine(O-36:5)	0.0947	0.6134	-0.0998	-0.6209	0.2719	0.0258	0.1564
Linoleoyl ethanolamide	-0.2901	0.0516	-0.4404	-0.5593	0.2524	0.0301	0.1757
Gamma-Glutamylglutamine	-0.2024	-0.5639	-0.0401	0.5091	0.2341	0.0332	0.1813
Phosphatidylcholine(38:2)	0.1234	0.6913	-0.0896	-0.5776	0.265	0.0331	0.1813
TG(46:1)	0.2819	0.7519	0.1056	-0.6775	0.3114	0.0334	0.1813
TG(56:1)	0.1433	0.803	-0.1041	-0.9131	0.4168	0.0322	0.1813
Octanoylcarnitine	0.1793	-0.0424	0.2768	0.5373	0.2491	0.0345	0.1842
Phosphatidylcholine(38:5)	-0.1359	0.4413	-0.3523	-0.5608	0.2607	0.0354	0.1853
TG(56:7)	-0.0628	0.426	-0.2462	-0.591	0.2767	0.0366	0.1887

2-Ketoglutaric acid	-0.1207	0.292	-0.3023	-0.5115	0.2462	0.0415	0.2103
L-Tyrosine	0.2558	0.0047	0.3685	0.4674	0.2271	0.0434	0.213
TG(44:1)	0.1767	0.6576	-0.0037	-0.6988	0.3378	0.0427	0.213
1-LG/2-LG	0.2767	0.6047	0.1324	-0.5287	0.2592	0.0453	0.2184
Lauroylcarnitine	0.2749	-0.0316	0.4098	0.565	0.278	0.046	0.2186
Phosphatidylcholine(36:3)	0.1563	0.6061	-0.0124	-0.5137	0.2538	0.0473	0.2211
Cystathionine	0.2592	0.0299	0.3621	0.449	0.2281	0.0532	0.2272
Gamma-L-glutamyl-L-alanine	-0.1385	0.1839	-0.2832	-0.5129	0.2588	0.0516	0.2272
Phosphatidylcholine(O-36:4)	0.218	0.6566	0.0535	-0.5979	0.3036	0.0534	0.2272
Phosphatidylcholine(O-38:5)	0.0782	0.5338	-0.0927	-0.6147	0.3096	0.0515	0.2272
Phosphatidylethanolamine(36:4)	0.3258	0.7807	0.1552	-0.4489	0.2288	0.0542	0.2272
Phosphatidylethanolamine(O-36:5)	0.002	0.4755	-0.1756	-0.6231	0.3159	0.053	0.2272
TG(46:2)	0.1592	0.637	-0.0199	-0.6498	0.3316	0.0545	0.2272
TG(58:2)	0.1106	0.5113	-0.0396	-0.6513	0.3288	0.0521	0.2272
17,18-DiHETE	-0.0879	-0.3519	0.0282	0.4664	0.2417	0.0579	0.2345
TG(56:4)	0.344	0.7018	0.2098	-0.5829	0.3016	0.0578	0.2345
L-Leucine	-0.0279	0.1112	-0.0904	-0.419	0.2179	0.0588	0.2351
Sphingomyelin(d18:1/22:0)	-0.054	0.2644	-0.1733	-0.5025	0.2663	0.0639	0.2522
Isocitrate	-0.6488	-0.3647	-0.7738	-0.4037	0.2176	0.0679	0.2647
Lysophosphatidylethanolamine (20:5)	-0.0843	-0.2868	0.0048	0.4395	0.243	0.0749	0.2884
Lysophosphatidylcholine(20:4)	-0.0787	0.1736	-0.1734	-0.4983	0.2779	0.0778	0.2956
Choline	-0.0302	0.1916	-0.1278	-0.4417	0.2546	0.0873	0.3125
L-Methionine	-0.2789	-0.1364	-0.3429	-0.3601	0.2082	0.0884	0.3125
L-Tryptophan	-0.0953	0.0959	-0.1812	-0.403	0.2329	0.0883	0.3125
Phosphatidylcholine(32:2)	0.0308	0.4854	-0.1396	-0.4661	0.2658	0.0844	0.3125
Phosphatidylcholine(40:6)	-0.1515	0.3152	-0.3266	-0.4144	0.2365	0.0847	0.3125
TG(46:0)	0.1436	0.5315	-0.0018	-0.6038	0.3455	0.0855	0.3125
Hexanoylcarnitine	0.2465	0.0796	0.32	0.4487	0.2629	0.0924	0.3182
Phosphatidylcholine(34:3)	0.0722	0.5524	-0.1079	-0.4636	0.2727	0.0942	0.3182
Phosphatidylcholine(40:8)	-0.1471	0.2228	-0.2858	-0.389	0.2285	0.0937	0.3182
TG(54:0)	-0.0408	0.284	-0.1626	-0.5039	0.2955	0.0932	0.3182

8,12-iPF2a IV	-0.1787	0.0171	-0.2649	-0.3271	0.1969	0.1012	0.338
Homocysteine	0.2049	-0.0944	0.3393	0.3439	0.2092	0.1049	0.3467
Propionylcarnitine	0.2703	0.4691	0.1828	-0.39	0.2382	0.1062	0.3471
Saccharopine	-0.2186	-0.5773	-0.0575	0.4022	0.2469	0.1081	0.3479
Sphingomyelin(d18:1/20:0)	0.0015	0.3698	-0.1365	-0.4507	0.277	0.1087	0.3479
Phosphatidylcholine(32:0)	0.0452	0.5011	-0.1258	-0.4627	0.2863	0.1111	0.3517
Phosphatidylcholine(O-38:6)	-0.0468	0.3913	-0.211	-0.4229	0.2648	0.1154	0.3596
Serotonine	-0.9166	-0.7388	-0.9964	-0.3882	0.2446	0.1171	0.3596
Tiglylcarnitine	0.0627	0.3024	-0.0427	-0.3907	0.246	0.117	0.3596
Lysophosphatidylcholine(14:0)	0.1278	0.4846	-0.006	-0.4347	0.2754	0.1196	0.3636
11-HETE	-0.5245	-0.8405	-0.3855	0.3451	0.2209	0.1228	0.366
Dodecenoylcarnitine	0.1004	-0.0985	0.188	0.4136	0.2643	0.1222	0.366
L-Valine	-0.0156	0.1495	-0.0897	-0.3708	0.2436	0.1327	0.3841
Sphingomyelin(d18:1/18:2)	-0.0685	0.2155	-0.175	-0.5034	0.3284	0.1304	0.3841
Tetradecenoylcarnitine	0.1194	-0.12	0.2248	0.4438	0.2914	0.1323	0.3841
Sphingomyelin(d18:1/24:0)	-0.0975	0.1024	-0.1725	-0.4094	0.2696	0.1341	0.3845
Sphingomyelin(d18:1/23:0)	0.1249	0.4937	-0.0134	-0.3884	0.2573	0.1362	0.387
8-HETE	-0.9123	-0.7831	-0.9692	-0.2767	0.188	0.1457	0.3956
Phosphatidylethanolamine(38:2)	0.2669	0.7018	0.1038	-0.3637	0.2441	0.1414	0.3956
Phosphatidylethanolamine(38:6)	0.1467	0.5444	-0.0024	-0.31	0.2098	0.1446	0.3956
TG(44:0)	0.096	0.4513	-0.0373	-0.5495	0.3706	0.1433	0.3956
TG(60:2)	0.0933	0.4418	-0.0375	-0.4597	0.3121	0.1458	0.3956
Phosphatidylethanolamine(O-38:7)	-0.0083	0.3695	-0.1499	-0.3794	0.2612	0.1515	0.4075
5,6-DiHETrE	0.1139	0.2757	0.0427	-0.4087	0.2844	0.1553	0.4108
Cer(d18:0/22:0)	0.1246	0.4735	-0.0063	-0.3517	0.2446	0.1554	0.4108
O-Anandamide	-0.0484	0.191	-0.1538	-0.3892	0.2761	0.1632	0.4276
TG(57:1)	-0.1305	0.2097	-0.258	-0.4406	0.317	0.1695	0.4404
8,9-DiHETrE	0.0407	0.1854	-0.0229	-0.2889	0.2096	0.1727	0.4449
11,12-DiHETrE	-0.1194	-0.0038	-0.1703	-0.3111	0.2295	0.1797	0.4547
DGLEA	0.0843	0.3556	-0.0351	-0.3584	0.2633	0.178	0.4547
Myristoilcarnitine	0.3237	0.1688	0.3918	0.4088	0.3025	0.181	0.4547

2-Methylbutyroylcarnitine	0.2041	0.3093	0.1579	-0.2987	0.2229	0.1847	0.4603
Palmitoylcarnitine	0.0577	-0.1379	0.1438	0.3967	0.2988	0.1888	0.4628
Phosphatidylcholine(36:2)	0.1625	0.4881	0.0404	-0.3365	0.2525	0.1875	0.4628
L-Glutamic acid	-0.2902	-0.1627	-0.3474	-0.37	0.2799	0.1908	0.464
Cortisol	0.3431	0.6717	0.1985	-0.3041	0.2331	0.1964	0.469
Glycodeoxycholic acid	0.0693	0.2556	-0.0127	-0.3364	0.2571	0.1952	0.469
TG(44:2)	0.0712	0.4005	-0.0522	-0.4535	0.3481	0.1975	0.469
L-Isoleucine	0.0587	0.1176	0.0322	-0.2864	0.2217	0.2008	0.4733
FA 22(6)	-0.3767	-0.0676	-0.5127	-0.31	0.2452	0.2103	0.4828
Glycolithocholic acid	-0.0785	0.2502	-0.2231	-0.3627	0.2874	0.2112	0.4828
Hexadecenoylcarnitine	0.0958	-0.0378	0.1546	0.3935	0.3116	0.2108	0.4828
Lysophosphatidylcholine(18:0)	0.073	0.3868	-0.0446	-0.3659	0.2867	0.2067	0.4828
Lysophosphatidic acid 18(1)	0.2307	0.138	0.2715	0.2903	0.2349	0.2207	0.5008
3-Hydroxypropionic acid	-0.2774	-0.1764	-0.3219	-0.2143	0.1776	0.2318	0.5219
Lysophosphatidylcholine(20:3)	0.0499	0.1378	0.0169	-0.3313	0.2801	0.2414	0.528
Lysophosphatidylethanolamine(20:4)	0.1474	0.4163	0.0465	-0.3699	0.311	0.2387	0.528
Sphingomyelin(d18:1/21:0)	0.1679	0.5395	0.0286	-0.3448	0.2915	0.2414	0.528
Sphingomyelin(d18:1/22:1)	-0.0447	0.2951	-0.1721	-0.3359	0.2812	0.2369	0.528
(+/-) 11-HDoHE	-10.286	-0.8562	-11.045	-0.2643	0.2296	0.2537	0.551
Phosphatidylethanolamine(34:2)	0.3449	0.6424	0.2333	-0.2873	0.252	0.2585	0.5573
Cer(d18:0/23:0)	0.1623	0.5293	0.0247	-0.3348	0.3017	0.2714	0.581
Anandamide	-0.074	0.119	-0.1589	-0.3011	0.2749	0.2774	0.5858
Phosphatidylcholine(36:1)	0.3289	0.6636	0.2033	-0.2808	0.2563	0.2775	0.5858
Sphingomyelin(d18:1/14:0)	0.2253	0.5458	0.1052	-0.2983	0.2744	0.2813	0.5879
Sphingomyelin(d18:1/16:1)	-0.0128	0.31	-0.1339	-0.3101	0.2859	0.2823	0.5879
DG(36:2)	0.0312	0.3616	-0.0927	-0.4083	0.3793	0.2859	0.5912
Lysophosphatidylcholine(16:0)	0.0486	0.2717	-0.0351	-0.3017	0.2857	0.2951	0.5933
Malic acid	-0.4488	-0.3007	-0.514	-0.276	0.2602	0.2925	0.5933
Prostaglandin F2α	-0.5032	-0.6532	-0.4372	0.188	0.1762	0.2898	0.5933
TG(58:9)	-0.2372	0.0639	-0.3501	-0.2594	0.2462	0.2962	0.5933
Taurolithocholic acid	0.0053	0.1867	-0.0745	-0.2918	0.2774	0.2967	0.5933

Phosphatidylcholine(O-38:4)	0.4073	0.6136	0.33	-0.2758	0.2644	0.301	0.5943
TG(58:8)	-0.1048	0.2044	-0.2207	-0.2657	0.2541	0.2997	0.5943
Decenoylcarnitine	-0.0711	-0.1663	-0.0293	0.2511	0.2452	0.3094	0.5982
Phosphatidylcholine(38:6)	-0.2226	0.119	-0.3507	-0.2354	0.2304	0.3108	0.5982
Sphingomyelin(d18:1/15:0)	0.3061	0.6635	0.1721	-0.2765	0.2707	0.3109	0.5982
TG(60:1)	0.1038	0.3288	0.0194	-0.344	0.3326	0.305	0.5982
Lysophosphatidic acid 18(2)	0.208	0.1489	0.234	0.2335	0.2317	0.3171	0.6025
TG(42:2)	-0.0278	0.2192	-0.1204	-0.3426	0.3384	0.3153	0.6025
DL-3-aminoisobutyric acid	0.2375	0.0822	0.3073	0.2656	0.2666	0.3228	0.6058
TG(55:1)	0.1635	0.3661	0.0876	-0.3318	0.3329	0.3227	0.6058
9-HOTrE	-0.1884	-0.3398	-0.1218	0.2798	0.2868	0.3328	0.6199
Sphingomyelin(d18:1/25:1)	0.1837	0.4801	0.0725	-0.234	0.2405	0.3344	0.6199
14,15-DiHETE	-0.2791	-0.4007	-0.2256	0.2178	0.2256	0.3376	0.622
Isovalerylcarnitine	0.044	0.0996	0.0196	-0.222	0.2316	0.3412	0.6249
L-Proline	-0.0426	0.0449	-0.0819	-0.2604	0.2749	0.3469	0.6303
Lysophosphatidylcholine(O-16:1)	-0.1324	0.0656	-0.2066	-0.2876	0.3043	0.3483	0.6303
DG(36:3)	-0.043	0.2579	-0.1558	-0.3382	0.3646	0.3573	0.6426
Pyroglutamic acid	-0.0761	0.0717	-0.141	-0.2754	0.3012	0.3637	0.6504
CE(22:6)	-0.2439	-0.184	-0.2664	0.2014	0.2227	0.3693	0.6565
Taurine	-0.9718	-0.8675	-10.187	-0.1791	0.1993	0.3719	0.6573
13-HODE	-0.681	-0.5847	-0.7233	-0.1931	0.2203	0.3837	0.6743
N6,N6,N6-Trimethyl-L-lysine	0.0518	-0.2384	0.1821	0.2352	0.2706	0.3877	0.6774
TG(45:0)	-0.0348	0.2123	-0.1275	-0.3257	0.3787	0.3932	0.683
12-HETE	-10.887	-10.402	-1.11	-0.1635	0.1916	0.3966	0.685
L-Glutamine	0.0979	-0.0698	0.1732	0.2107	0.2499	0.4022	0.6908
Oleoylcarnitine	-0.1027	-0.2148	-0.0534	0.248	0.296	0.405	0.6917
Phosphatidylcholine(40:7)	-0.1472	0.1744	-0.2677	-0.187	0.2278	0.415	0.7016
Pimelylcarnitine	-0.1108	-0.2876	-0.0331	0.2027	0.2474	0.4154	0.7016
1-Methylhistidine	0.2519	0.3609	0.203	-0.2134	0.2716	0.4349	0.7079
19,20-DiHDPA	-0.2123	-0.2438	-0.1984	0.1586	0.2106	0.454	0.7079
8(9)-EpETrE	-0.9269	-0.8689	-0.9525	-0.1816	0.2381	0.4482	0.7079

CE(18:1)	0.1307	0.4395	0.0149	-0.2115	0.2639	0.4259	0.7079
Ethanolamine	-0.0402	-0.1817	0.0233	0.1587	0.2114	0.4553	0.7079
Isobutyrylcarnitine	0.1916	0.3197	0.1353	-0.2211	0.2919	0.4515	0.7079
L-Arginine	0.0849	0.2373	0.0165	-0.1988	0.2607	0.4485	0.7079
Lysophosphatidic acid 14(0)	0.2196	0.5367	0.0801	-0.1671	0.223	0.4563	0.7079
Lysophosphatidylethanolamine (20:4)	0.2711	0.3957	0.2162	-0.1753	0.2272	0.4431	0.7079
Lysophosphatidylethanolamine(18:0)	0.2663	0.5169	0.1724	-0.2056	0.2714	0.4516	0.7079
Phosphatidylcholine(34:1)	0.106	0.1796	0.0784	-0.0521	0.0695	0.4564	0.7079
Prostaglandin E2	-0.4642	-0.6049	-0.4023	0.1906	0.2447	0.4387	0.7079
Symmetric dimethylarginine	0.1868	0.1013	0.2252	0.182	0.2393	0.4495	0.7079
Sphingomyelin(d18:1/16:0)	0.0237	0.1626	-0.0284	-0.2373	0.2978	0.4285	0.7079
Sphingomyelin(d18:1/24:1)	-0.0759	0.0639	-0.1284	-0.2151	0.276	0.4387	0.7079
Tetradecadienylcarnitine	-0.1118	-0.2593	-0.0469	0.2226	0.2847	0.4369	0.7079
Glycine	-0.088	-0.1379	-0.0656	0.1965	0.2646	0.4604	0.7105
L-Alpha-aminobutyric acid	-0.0889	0.0133	-0.1348	-0.1854	0.2547	0.4692	0.7204
Glycochenodeoxycholic acid	0.1364	0.1004	0.1522	-0.1736	0.2431	0.4777	0.726
TG(42:1)	0.0347	0.1607	-0.0125	-0.2492	0.3473	0.4758	0.726
Gamma-aminobutyric acid	-0.3601	-0.0601	-0.4948	-0.2062	0.2942	0.4859	0.7262
L-Histidine	-0.1792	-0.1022	-0.2137	-0.1784	0.2548	0.4861	0.7262
Nonaylcarnitine	0.2613	0.389	0.2052	-0.1669	0.239	0.4873	0.7262
Taurodeoxycholic acid	0.2309	-0.0276	0.3447	0.1729	0.2452	0.4831	0.7262
O-Phosphoethanolamine	-1.034	-0.9736	-10.611	-0.1505	0.2185	0.4934	0.7317
Cer(d18:1/23:0)	0.29	0.5063	0.209	-0.1705	0.2516	0.5005	0.7387
14,15-DiHETrE	-0.0576	-0.0204	-0.074	-0.1559	0.2411	0.5201	0.7572
Deoxycholic acid	0.0718	0.1881	0.0206	-0.1743	0.27	0.5206	0.7572
Phosphatidylcholine(34:2)	0.0806	0.266	0.011	-0.1693	0.2591	0.5159	0.7572
Lysophosphatidylethanolamine (16:0)	0.0106	0.0037	0.0136	0.1599	0.2516	0.5272	0.7632
Citrulline	0.0713	0.2024	0.0125	-0.1718	0.2754	0.535	0.7708
Lysophosphatidic acid 16(1)	0.119	0.1994	0.0836	0.1317	0.215	0.5422	0.7751
Stearoylcarnitine	0.2874	0.2326	0.3116	0.1744	0.2853	0.5431	0.7751
Phosphatidylcholine(32:1)	0.1614	0.3977	0.0727	-0.1615	0.2706	0.5528	0.7853

Palmitoyl ethanolamide	0.014	0.2129	-0.0736	-0.1529	0.2612	0.5601	0.792
Phosphatidylcholine(36:5)	-0.1604	0.1348	-0.2711	-0.148	0.2589	0.5695	0.8015
Carnitine	0.2227	0.1203	0.2678	0.1399	0.2553	0.5854	0.8067
FA 18(2)	-0.4552	-0.4183	-0.4714	-0.1377	0.2524	0.5871	0.8067
Lysophosphatidylcholine(18:3)	0.0741	0.1319	0.0523	-0.1336	0.2449	0.5874	0.8067
Lysophosphatidylethanolamine(18:1)	0.0339	-0.1613	0.1071	0.2018	0.3669	0.5842	0.8067
Lysophosphatidylinositol 18(1)	0.4058	0.211	0.4915	0.1587	0.2836	0.5776	0.8067
Trimethylamine N-oxide	0.0829	0.0205	0.1103	0.1395	0.2571	0.5891	0.8067
TG(42:0)	0.0421	0.1763	-0.0082	-0.2113	0.3924	0.5922	0.8074
9,10-DiHOME	-0.4625	-0.3998	-0.4901	-0.1328	0.2531	0.6014	0.8143
Taurocholic acid	0.0921	-0.1246	0.1875	0.1232	0.2355	0.6027	0.8143
Lysophosphatidylethanolamine (16:1)	0.2258	0.1692	0.2507	0.1221	0.2388	0.6108	0.8215
12(S)-HEPE	-1.071	-10.429	-10.833	-0.1023	0.2033	0.6165	0.822
L-Phenylalanine	-0.108	-0.0958	-0.1135	-0.1175	0.2333	0.6162	0.822
Butyrylcarnitine	0.0235	0.0951	-0.008	-0.1077	0.2191	0.6247	0.8243
Glycocholic acid	-0.0509	-0.131	-0.0156	-0.1062	0.2171	0.6263	0.8243
Sarcosine	0.3331	0.1953	0.395	0.1236	0.2521	0.6255	0.8243
(+/-) 14-HDoHE	-10.543	-10.161	-1.071	-0.0985	0.2038	0.6306	0.8253
Cysteine	0.2298	0.3564	0.1729	-0.1054	0.223	0.638	0.8253
L-Asparagine	-0.3231	-0.2872	-0.3392	-0.1172	0.2478	0.6378	0.8253
L-Lysine	-0.2146	-0.0832	-0.2736	-0.1114	0.2342	0.6358	0.8253
CE(18:2)	-0.109	0.0899	-0.1836	-0.1172	0.2567	0.6497	0.8264
L-Homoserine	-0.1142	-0.2394	-0.058	0.1096	0.2364	0.6443	0.8264
Lysophosphatidylinositol 20(4)	0.134	0.0917	0.1527	-0.1062	0.2288	0.6439	0.8264
Sphingomyelin(d18:1/18:0)	0.0985	0.3182	0.016	-0.1152	0.2507	0.6475	0.8264
Lysophosphatidylinositol 16(1)	0.1305	-0.0037	0.1896	0.1176	0.2658	0.6596	0.8354
L-Kynurenine	0.1563	0.2478	0.1153	-0.1252	0.2868	0.6637	0.8367
Lysophosphatidylcholine(20:5)	-0.1783	-0.2015	-0.1696	0.1014	0.2338	0.666	0.8367
Lysophosphatidylethanolamine(22:6)	-0.1078	0.1128	-0.1905	-0.1133	0.2651	0.6707	0.839
Phosphatidylcholine(O-34:3)	-0.0037	0.151	-0.0617	-0.1073	0.2694	0.6917	0.8618
Citric acid	-0.0384	0.1141	-0.1055	-0.0882	0.2262	0.6978	0.8659

15(S)-HETrE	-0.794	-10.128	-0.6977	0.0807	0.2357	0.7332	0.8741
5-HETE	-0.3028	-0.1829	-0.3555	-0.0911	0.251	0.7179	0.8741
Glutathione	-0.71	-0.6959	-0.7164	-0.0877	0.2591	0.7361	0.8741
Glycoursodeoxycholic acid	0.2633	0.2253	0.28	-0.1064	0.2975	0.7218	0.8741
L-4-hydroxy-proline	0.1087	-0.0453	0.1778	0.0937	0.2753	0.7348	0.8741
Lysophosphatidylcholine(O-18:1)	-0.5235	-0.5557	-0.5114	-0.1021	0.2939	0.7295	0.8741
Lysophosphatidylinositol 18(2)	-0.0846	-0.1483	-0.0566	-0.0893	0.2518	0.724	0.8741
Octenoylcarnitine	0.0018	-0.0881	0.0414	0.0892	0.2613	0.734	0.8741
Phosphatidylcholine(O-34:1)	0.1517	0.2135	0.1285	0.0932	0.2727	0.7337	0.8741
Phosphatidylcholine(O-36:3)	0.0871	0.1994	0.0449	-0.0794	0.2168	0.7155	0.8741
TG(59:1)	-0.0251	0.1098	-0.0758	-0.0986	0.2632	0.7092	0.8741
Betaine	-0.0982	-0.1411	-0.0794	-0.0689	0.2084	0.7419	0.8776
Sphingosine 1-phosphate 18(0)	-10.768	-10.546	-10.865	-0.0601	0.1955	0.7597	0.8924
Sphingomyelin(d18:1/18:1)	-0.048	0.1006	-0.1037	0.0784	0.259	0.7632	0.8924
Sphingomyelin(d18:1/25:0)	0.1335	0.1777	0.1169	-0.0988	0.3256	0.7627	0.8924
Sphingomyelin(d18:1/20:1)	-0.2104	-0.1821	-0.221	0.0818	0.28	0.7712	0.8982
Cer(d18:1/24:1)	0.0672	0.2108	0.0134	-0.0854	0.2993	0.7764	0.9008
Lysophosphatidylcholine(16:1)	0.0727	0.1795	0.0327	-0.0799	0.2906	0.7843	0.9066
12,13-DiHOME	-0.1143	-0.1381	-0.1038	0.0756	0.2875	0.7932	0.9134
Lysophosphatidylinositol 18(0)	0.228	0.3393	0.179	-0.0581	0.2345	0.8052	0.9168
LPS 18(1)	-0.9754	-10.108	-0.9598	-0.0542	0.2161	0.8026	0.9168
O-Acetyl-L-serine	-0.1701	-0.0502	-0.2239	-0.0569	0.2253	0.8015	0.9168
(+/-) 8-HDoHE	-0.2765	-0.1277	-0.3419	-0.0657	0.2767	0.813	0.9204
Sphingosine 1-phosphate 18(2)	-0.9801	-0.9897	-0.9758	0.0415	0.176	0.8145	0.9204
Deoxycarnitine	-0.0502	-0.1663	0.0008	0.0608	0.2852	0.8318	0.9284
Dehydroepiandrosterone	-0.0518	0.0262	-0.0862	-0.0549	0.2608	0.8338	0.9284
Lysophosphatidylcholine(O-16:0)	-0.1532	-0.1751	-0.145	-0.0708	0.3214	0.8262	0.9284
TG(56:0)	-0.064	-0.0596	-0.0656	0.0812	0.3721	0.828	0.9284
Sphingomyelin(d18:1/23:1)	0.0556	0.2902	-0.0323	-0.0525	0.2723	0.8477	0.937
Thromboxane B2	-0.4723	-0.5612	-0.4332	0.0463	0.2365	0.8454	0.937
9,10,13-TriHOME	0.4223	0.3968	0.4335	0.0404	0.2267	0.8592	0.946

9,12,13-TriHOME	0.318	0.2918	0.3295	0.0429	0.2457	0.862	0.946
Glycylglycine	-0.0935	-0.0791	-0.0999	0.0426	0.264	0.8724	0.9539
Hydroxylysine	0.097	0.1179	0.0876	0.0442	0.2891	0.879	0.9578
L-Serine	-0.1882	-0.1423	-0.2087	0.0318	0.2243	0.8878	0.9608
Phosphatidylcholine(O-34:2)	0.1798	0.218	0.1655	0.0301	0.213	0.8881	0.9608
Methionine sulfone	0.1264	0.3092	0.0443	-0.0359	0.2773	0.8974	0.9675
Sphingomyelin(d18:1/24:2)	-0.1762	-0.0513	-0.2231	-0.0352	0.2903	0.9038	0.9709
9-HODE	-0.4205	-0.4457	-0.4094	-0.0271	0.2476	0.9133	0.9742
Linoleylcarnitine	-0.5231	-0.5618	-0.506	-0.0282	0.2496	0.9104	0.9742
(+/-) 10-HDoHE	-10.272	-10.335	-10.244	-0.0115	0.1839	0.9501	0.9759
12,13-EpOME	0.0361	0.0315	0.0381	0.0247	0.3392	0.9421	0.9759
Acetylcarnitine	0.0766	0.123	0.0561	-0.015	0.2554	0.9535	0.9759
Cholic acid	0.0115	-0.1204	0.0695	0.0227	0.2693	0.9331	0.9759
Homocitrulline	0.1085	0.1918	0.071	-0.0245	0.2689	0.9276	0.9759
Lysophosphatidic acid 16(0)	0.1487	0.3953	0.0402	0.0232	0.2398	0.9231	0.9759
Lysophosphatidylcholine(18:2)	0.0601	-0.1007	0.1204	0.0229	0.2835	0.9359	0.9759
Lysophosphatidylcholine(22:6)	-0.3121	-0.2974	-0.3176	0.0141	0.2391	0.9532	0.9759
Malonylcarnitine	-0.0179	0.0062	-0.0286	0.0153	0.2206	0.9449	0.9759
Phosphatidylcholine(O-44:5)	-0.053	-0.1509	-0.0163	-0.0166	0.2785	0.9527	0.9759
S-Methylcysteine	-0.2455	-0.1905	-0.2702	-0.0148	0.2525	0.9533	0.9759
Valerylcarnitine	0.2163	0.2367	0.2073	0.0197	0.2435	0.9357	0.9759
3-Hydroxybutyric acid	-0.0436	-0.0558	-0.0382	0.0104	0.2673	0.9692	0.9887
FA 18(1)	-0.1758	-0.0733	-0.2209	-0.0076	0.2634	0.9772	0.9902
Taurochenodeoxycholic acid	0.2256	0.1765	0.2472	-0.0077	0.2556	0.9759	0.9902
Cer(d18:1/22:0)	0.1629	0.2763	0.1204	-0.0052	0.2106	0.9805	0.9903
12,13-DiHODE	-0.0224	-0.0298	-0.0192	-0.0033	0.2685	0.9902	0.9967
Lysophosphatidylcholine(18:1)	0.1816	0.142	0.1964	0.0009	0.2943	0.9975	0.9983
Sphingosine 1-phosphate 18(1)	-10.832	-11.179	-10.679	0.0004	0.1904	0.9983	0.9983

Table S8. Results of the discovery/replication design analysis for all metabolites differently expressed in L-Dopa^{positive} IPD patients compared to healthy controls

metabolite	Discovery beta	Discovery p-value	Replication beta	Replication p-value	Replication p-value adjusted
3-Methoxytyramine	1.8922	<0.0001	2.0205	<0.0001	<0.0001
Methyldopa	1.6281	<0.0001	1.4321	<0.0001	<0.0001
N6.n6.n6.trimethyl_lysine	0.6416	0.0024	0.2352	0.3877	0.7270
DCA	0.5925	0.0074	-0.1743	0.5206	0.7471
GCA	-0.5072	0.0138	-0.1062	0.6263	0.7471
Deoxycarnitine	-0.3449	0.0157	0.0608	0.8318	0.8318
Gamma_glutamyl_alanine	0.5425	0.0209	-0.5129	0.0516	0.1547
TCA	-0.4905	0.0255	0.1232	0.6027	0.7471
GCDCA	-0.4779	0.0280	-0.1736	0.4777	0.7471
Ornithine	0.5273	0.0288	0.6153	0.0014	0.0052
SM_d18.1.18.0	0.5050	0.0360	-0.1152	0.6475	0.7471
GUDCA	-0.3586	0.0397	-0.1064	0.7218	0.7734
Homocysteine	0.4759	0.0431	0.3439	0.1049	0.2330
Putrescine	0.3053	0.0459	0.9844	<0.0001	<0.0001
SM-d18.1.20.0	0.5068	0.0489	-0.4507	0.1087	0.2330

Table S9. Metabolites differently expressed in IPD patients without L-Dopa treatment (L-Dopa^{negative}, n=30) compared to controls (n=64)

Name	z-value	Nominal *p*	Adjusted *p*	Beta (CI)
Lysophosphatidic acid 16(0)	-3.418	0.000631	0.0959	-0.685 (-1.078;-0.292)
1-AG/2-AG	-3.589	0.000332	0.0959	-0.762 (-1.178;-0.346)
Glutathione	2.751	0.00594	0.301	0.432 (0.124;0.74)
1-LG/2-LG	-2.766	0.00567	0.301	-0.573 (-0.978;-0.167)
Phosphatidylcholine(36:4)	-2.858	0.00426	0.301	-0.634 (-1.069;-0.199)
Phosphatidylcholine(38:7)	-2.764	0.0057	0.301	-0.571 (-0.975;-0.166)
Citric acid	2.618	0.00885	0.384	0.57 (0.143;0.997)
Hexadecenoylcarnitine	2.532	0.0114	0.432	0.533 (0.12;0.946)
Phosphatidylethanolamine(34:2)	-2.458	0.014	0.449	-0.606 (-1.089;-0.123)
Phosphatidylethanolamine(36:4)	-2.438	0.0148	0.449	-0.58 (-1.046;-0.114)
Oleoylcarnitine	2.383	0.0172	0.474	0.505 (0.09;0.92)
Tiglylcarnitine	-2.321	0.0203	0.514	-0.61 (-1.126;-0.095)
Putrescine	2.227	0.026	0.56	0.386 (0.046;0.725)
Lysophosphatidic acid 14(0)	-2.107	0.0351	0.56	-0.398 (-0.768;-0.028)
Glycoursodeoxycholic acid	-2.117	0.0342	0.56	-0.449 (-0.865;-0.033)
Thromboxane-B2	-2.132	0.033	0.56	-0.374 (-0.718;-0.03)
Phosphatidylcholine(O-36:5)	-2.088	0.0368	0.56	-0.505 (-0.978;-0.031)
TG(50:1)	-2.195	0.0282	0.56	-0.49 (-0.928;-0.052)
TG(52:1)	-2.176	0.0296	0.56	-0.49 (-0.932;-0.049)
TG(54:7)	-2.154	0.0313	0.56	-0.515 (-0.984;-0.046)
FA 18(1)	2.024	0.043	0.623	0.486 (0.015;0.956)
L-2-aminoadipic acid	-1.745	0.0811	0.653	-0.315 (-0.669;0.039)
L-Arginine	1.799	0.072	0.653	0.407 (-0.036;0.851)
L-Serine	01. Aug	0.0719	0.653	0.47 (-0.042;0.982)
Tetradecenoylcarnitine	1.963	0.0497	0.653	0.445 (0.001;0.89)
Lysophosphatidic acid 18(2)	-1.882	0.0598	0.653	-0.411 (-0.84;0.017)
LPS 18(1)	-1.81	0.0704	0.653	-0.257 (-0.536;0.021)
(+/-) 11-HDoHE	1.717	0.086	0.653	0.234 (-0.033;0.501)
19,20-DiHDPA	1.721	0.0853	0.653	0.458 (-0.064;0.979)
(+/-) 8-HDoHE	Jan 72	0.0855	0.653	0.443 (-0.062;0.949)
Prostaglandin E2	-1.744	0.0812	0.653	-0.305 (-0.648;0.038)
Phosphatidylcholine(38:4)	-1.723	0.0848	0.653	-0.402 (-0.86;0.055)
Phosphatidylethanolamine(O-36:5)	-1.855	0.0636	0.653	-0.543 (-1.117;0.031)
TG(50:2)	-1.805	0.0711	0.653	-0.405 (-0.844;0.035)
TG(50:3)	-1.773	0.0762	0.653	-0.42 (-0.885;0.044)
TG(50:4)	-1.797	0.0724	0.653	-0.449 (-0.938;0.041)
TG(52:4)	-1.726	0.0844	0.653	-0.414 (-0.885;0.056)
TG(54:1)	-1.928	0.0538	0.653	-0.522 (-1.053;0.009)
TG(54:5)	-1.855	0.0635	0.653	-0.46 (-0.946;0.026)
TG(56:6)	-1.968	0.049	0.653	-0.478 (-0.955;-0.002)

13-HODE	1.693	0.0904	0.67	0.34 (-0.054;0.734)
Cer(d18:1/22:0)	-1.657	0.0975	0.674	-0.334 (-0.73;0.061)
DG(36:2)	-1.659	0.097	0.674	-0.14 (-0.305;0.025)
TG(48:1)	-1.67	0.0949	0.674	-0.391 (-0.849;0.068)
Citrulline	1.345	0.178	0.711	0.243 (-0.111;0.596)
Gamma-Glutamylglutamine	1.221	0.222	0.711	0.287 (-0.174;0.748)
L-Glutamic acid	-1.449	0.147	0.711	-0.322 (-0.757;0.114)
L-Kynurenine	-1.269	0.204	0.711	-0.313 (-0.796;0.17)
Saccharopine	1.342	0.18	0.711	0.335 (-0.154;0.823)
Sarcosine	1.241	0.215	0.711	0.27 (-0.156;0.697)
Serotonine	1.145	0.252	0.711	0.14 (-0.099;0.378)
L-Threonine	1.394	0.163	0.711	0.363 (-0.147;0.873)
2-Methylbutyroylcarnitine	-1.371	0.17	0.711	-0.323 (-0.784;0.139)
Acetylcarnitine	1.171	0.241	0.711	0.277 (-0.186;0.74)
Betaine	1.196	0.232	0.711	0.311 (-0.199;0.821)
Dodecenoylcarnitine	1.595	0.111	0.711	0.365 (-0.084;0.814)
Isobutyrylcarnitine	-1.275	0.202	0.711	-0.302 (-0.766;0.162)
Myristoilcarnitine	1.278	0.201	0.711	0.269 (-0.144;0.682)
Octanoylcarnitine	1.133	0.257	0.711	0.279 (-0.204;0.763)
Palmitoylcarnitine	Jan 33	0.183	0.711	0.269 (-0.127;0.665)
Propionylcarnitine	-1.37	0.171	0.711	-0.321 (-0.78;0.138)
Tetradecadienylcarnitine	Jan 17	0.242	0.711	0.269 (-0.182;0.719)
2-Ketoglutaric acid	-1.198	0.231	0.711	-0.267 (-0.705;0.17)
FA 22(6)	1.155	0.248	0.711	0.282 (-0.197;0.761)
Lysophosphatidic acid 16(1)	-1.544	0.123	0.711	-0.302 (-0.686;0.081)
Lysophosphatidic acid 18(1)	-1.296	0.195	0.711	-0.283 (-0.711;0.145)
Lysophosphatidylethanolamine (20:4)	-1.184	0.237	0.711	-0.311 (-0.825;0.204)
(+/-) 10-HDoHE	1.331	0.183	0.711	0.183 (-0.086;0.452)
12(S)-HEPE	1.161	0.246	0.711	0.117 (-0.081;0.315)
14,15-DiHETE	1.198	0.231	0.711	0.308 (-0.196;0.813)
(+/-) 14-HDoHE	1.433	0.152	0.711	0.133 (-0.049;0.316)
17,18-DiHETE	1.367	0.172	0.711	0.35 (-0.152;0.852)
5,6-DiHETrE	-1.174	0.24	0.711	-0.292 (-0.78;0.196)
9,10,13-TriHOME	1.218	0.223	0.711	0.228 (-0.139;0.594)
9,12,13-TriHOME	1.257	0.209	0.711	0.295 (-0.165;0.756)
Cortisol	1.232	0.218	0.711	0.306 (-0.181;0.792)
Dehydroepiandrosterone	1.533	0.125	0.711	0.394 (-0.11;0.897)
Glycochenodeoxycholic acid	-1.593	0.111	0.711	-0.329 (-0.733;0.076)
Linoleoyl ethanolamide	1.143	0.253	0.711	0.271 (-0.194;0.736)
Palmitoyl ethanolamide	Jan 23	0.219	0.711	0.309 (-0.183;0.801)
CE(20:4)	-1.564	0.118	0.711	-0.384 (-0.866;0.097)
CE(22:6)	1.231	0.218	0.711	0.322 (-0.19;0.834)
Cer(d18:0/22:0)	-1.207	0.228	0.711	-0.319 (-0.837;0.199)

Lysophosphatidylcholine(16:0)	-1.613	0.107	0.711	-0.393 (-0.87;0.084)
Lysophosphatidylcholine(22:6)	1.469	0.142	0.711	0.42 (-0.14;0.981)
Lysophosphatidylethanolamine(18:1)	-1.146	0.252	0.711	-0.082 (-0.222;0.058)
Phosphatidylcholine(36:3)	-1.318	0.188	0.711	-0.286 (-0.712;0.139)
Phosphatidylcholine(38:3)	-1.267	0.205	0.711	-0.286 (-0.729;0.157)
Phosphatidylcholine(40:5)	-1.26	0.208	0.711	-0.315 (-0.805;0.175)
Phosphatidylcholine(O-36:3)	1.217	0.224	0.711	0.208 (-0.127;0.543)
Phosphatidylethanolamine(O-38:5)	-1.466	0.143	0.711	-0.452 (-1.055;0.152)
Sphingomyelin(d18:1/16:0)	-1.446	0.148	0.711	-0.333 (-0.785;0.119)
Sphingomyelin(d18:1/16:1)	-1.231	0.218	0.711	-0.273 (-0.708;0.162)
Sphingomyelin(d18:1/25:1)	01. Apr	0.162	0.711	0.362 (-0.145;0.87)
TG(45:0)	1.509	0.131	0.711	0.183 (-0.055;0.421)
TG(46:1)	-1.138	0.255	0.711	-0.238 (-0.648;0.172)
TG(48:2)	-1.573	0.116	0.711	-0.37 (-0.831;0.091)
TG(48:3)	-1.211	0.226	0.711	-0.285 (-0.745;0.176)
TG(51:1)	-1.188	0.235	0.711	-0.26 (-0.688;0.169)
TG(51:2)	-1.135	0.256	0.711	-0.26 (-0.71;0.189)
TG(51:3)	-1.337	0.181	0.711	-0.315 (-0.776;0.147)
TG(52:2)	-1.337	0.181	0.711	-0.299 (-0.737;0.139)
TG(52:3)	-1.576	0.115	0.711	-0.371 (-0.832;0.09)
TG(54:2)	-1.175	0.24	0.711	-0.253 (-0.675;0.169)
TG(54:4)	-1.268	0.205	0.711	-0.32 (-0.815;0.175)
TG(56:0)	1.161	0.246	0.711	0.177 (-0.122;0.476)
TG(56:5)	-1.466	0.143	0.711	-0.349 (-0.817;0.118)
TG(56:7)	-1.16	0.246	0.711	-0.291 (-0.783;0.201)
TG(57:1)	1.558	0.119	0.711	0.278 (-0.072;0.627)
TG(59:1)	1.501	0.133	0.711	0.29 (-0.089;0.669)
Phosphatidylcholine(34:1)	-1.104	0.27	0.738	-0.06 (-0.167;0.047)
DL-3-aminoisobutyric acid	-1.073	0.283	0.769	-0.263 (-0.743;0.217)
Decanoylcarnitine	1.053	0.292	0.78	0.251 (-0.217;0.719)
Methylmalonylcarnitine	-1.057	0.291	0.78	-0.275 (-0.784;0.235)
Pimelylcarnitine	1.044	0.297	0.784	0.229 (-0.201;0.659)
Phosphatidylcholine(34:4)	-1.026	0.305	0.792	-0.246 (-0.717;0.224)
TG(46:0)	-1.031	0.302	0.792	-0.182 (-0.528;0.164)
Ethanolamine	1.011	0.312	0.792	0.229 (-0.215;0.674)
Pyroglutamic acid	-1.009	0.313	0.792	-0.269 (-0.792;0.253)
Lysophosphatidylcholine(20:5)	1.018	0.309	0.792	0.227 (-0.21;0.664)
9-HOTrE	0.995	0.32	0.797	0.224 (-0.217;0.664)
Phosphatidylcholine(O-44:5)	0.999	0.318	0.797	0.263 (-0.253;0.78)
Lauroylcarnitine	0.988	0.323	0.798	0.229 (-0.225;0.682)
3-Hydroxybutyric acid	0.953	0.34	0.803	0.249 (-0.263;0.762)
Lysophosphatidylinositol 16(1)	-0.96	0.337	0.803	-0.221 (-0.671;0.23)
Prostaglandin F2α	0.965	0.335	0.803	0.173 (-0.179;0.525)

Phosphatidylethanolamine(38:4)	-0.977	0.329	0.803	-0.23 (-0.692;0.231)
TG(44:1)	-0.969	0.333	0.803	-0.167 (-0.504;0.171)
TG(51:4)	-0.953	0.341	0.803	-0.219 (-0.67;0.232)
L-Homoserine	0.907	0.364	0.822	0.173 (-0.201;0.547)
Sphingosine 1-phosphate 18(0)	-0.895	0.371	0.822	-0.093 (-0.298;0.111)
12,13-DiHOME	0.899	0.369	0.822	0.228 (-0.269;0.724)
8(9)-EpETrE	0.891	0.373	0.822	0.122 (-0.146;0.39)
Lysophosphatidylcholine(20:3)	-0.911	0.362	0.822	-0.217 (-0.685;0.25)
Sphingomyelin(d18:1/18:2)	-0.895	0.371	0.822	-0.288 (-0.918;0.342)
TG(42:0)	-0.892	0.372	0.822	-0.084 (-0.267;0.1)
TG(42:1)	-0.893	0.372	0.822	-0.162 (-0.517;0.193)
TG(46:2)	-0.915	0.36	0.822	-0.171 (-0.536;0.195)
Malonylcarnitine	0.88	0.379	0.829	0.199 (-0.244;0.642)
TG(56:1)	0.869	0.385	0.835	0.116 (-0.146;0.378)
Nonaylcarnitine	-0.839	0.401	0.859	-0.217 (-0.723;0.29)
Sphingomyelin(d18:1/20:1)	0.843	0.399	0.859	0.191 (-0.253;0.635)
Lysophosphatidylcholine(20:4)	-0.832	0.405	0.862	-0.212 (-0.712;0.288)
Glycine	0.755	0.45	0.864	0.166 (-0.265;0.597)
Hydroxylysine	-0.691	0.49	0.864	-0.19 (-0.729;0.349)
L-Lysine	0.739	0.46	0.864	0.2 (-0.33;0.729)
O-Acetyl-L-serine	-0.746	0.456	0.864	-0.174 (-0.63;0.283)
L-Proline	-0.761	0.447	0.864	-0.187 (-0.667;0.294)
L-Tyrosine	-0.692	0.489	0.864	-0.147 (-0.564;0.27)
Stearoylcarnitine	0.795	0.426	0.864	0.18 (-0.264;0.624)
Trimethylamine N-oxide	0.767	0.443	0.864	0.147 (-0.228;0.522)
Lactic acid	-0.688	0.491	0.864	-0.145 (-0.558;0.268)
Lysophosphatidylethanolamine (16:0)	-0.683	0.494	0.864	-0.184 (-0.714;0.345)
Lysophosphatidylethanolamine (16:1)	-0.693	0.488	0.864	-0.161 (-0.615;0.294)
Lysophosphatidylinositol 18(1)	-0.739	0.46	0.864	-0.171 (-0.626;0.283)
12,13-DiHODE	0.823	0.41	0.864	0.161 (-0.222;0.544)
8,12-iPF2a IV	-0.685	0.493	0.864	-0.148 (-0.571;0.275)
Anandamide	0.728	0.466	0.864	0.184 (-0.312;0.681)
Cholic acid	-0.719	0.472	0.864	-0.142 (-0.53;0.245)
O-Anandamide	0.805	0.421	0.864	0.198 (-0.285;0.681)
CE(18:2)	0.779	0.436	0.864	0.196 (-0.296;0.688)
Lysophosphatidylcholine(O-16:1)	-0.688	0.492	0.864	-0.165 (-0.633;0.304)
Phosphatidylcholine(34:2)	-0.732	0.464	0.864	-0.176 (-0.647;0.295)
Phosphatidylcholine(40:6)	0.753	0.452	0.864	0.183 (-0.293;0.658)
Phosphatidylcholine(40:7)	0.706	0.48	0.864	0.165 (-0.293;0.624)
Phosphatidylcholine(O-36:4)	-0.697	0.486	0.864	-0.188 (-0.718;0.341)
Phosphatidylethanolamine(38:6)	-0.732	0.464	0.864	-0.191 (-0.703;0.321)
Sphingomyelin(d18:1/23:0)	-0.683	0.495	0.864	-0.175 (-0.678;0.328)
TG(42:2)	-0.794	0.427	0.864	-0.166 (-0.575;0.244)

TG(44:0)	-0.812	0.417	0.864	-0.1 (-0.341;0.141)
TG(54:3)	-0.792	0.429	0.864	-0.183 (-0.635;0.269)
TG(56:3)	-0.797	0.425	0.864	-0.169 (-0.586;0.247)
TG(56:4)	-0.692	0.489	0.864	-0.166 (-0.634;0.303)
TG(60:2)	0.753	0.451	0.864	0.162 (-0.259;0.582)
Linoleylcarnitine	0.643	0.52	0.888	0.127 (-0.26;0.514)
Malic acid	0.656	0.512	0.888	0.128 (-0.255;0.511)
Taurodeoxycholic acid	-0.647	0.518	0.888	-0.166 (-0.669;0.337)
TG(58:9)	-0.65	0.516	0.888	-0.152 (-0.61;0.306)
Gamma-aminobutyric acid	-0.633	0.526	0.889	-0.125 (-0.511;0.261)
Hexanoylcarnitine	0.635	0.525	0.889	0.155 (-0.323;0.634)
Phosphatidylcholine(O-34:1)	0.626	0.531	0.889	0.131 (-0.279;0.54)
Phosphatidylcholine(O-38:6)	0.624	0.532	0.889	0.177 (-0.38;0.735)
Cysteine	0.523	0.601	0.894	0.115 (-0.315;0.545)
O-Phosphoethanolamine	0.524	0.6	0.894	0.048 (-0.132;0.228)
Ornithine	0.6	0.549	0.894	0.139 (-0.316;0.595)
L-Phenylalanine	-0.581	0.561	0.894	-0.151 (-0.661;0.358)
S-Methylcysteine	-0.52	0.603	0.894	-0.12 (-0.57;0.331)
Taurine	0.549	0.583	0.894	0.069 (-0.178;0.316)
Carnitine	-0.572	0.567	0.894	-0.149 (-0.658;0.361)
Isocitrate	-0.576	0.565	0.894	-0.107 (-0.47;0.256)
11,12-DiHETrE	0.531	0.596	0.894	0.132 (-0.356;0.621)
12-HETE	0.53	0.596	0.894	0.053 (-0.144;0.25)
15(S)-HETrE	0.578	0.563	0.894	0.114 (-0.273;0.501)
8-HETE	0.574	0.566	0.894	0.106 (-0.255;0.467)
9,10-DiHOME	0.528	0.598	0.894	0.124 (-0.338;0.587)
9-HODE	0.524	0.6	0.894	0.107 (-0.292;0.506)
Glycodeoxycholic acid	-0.535	0.593	0.894	-0.136 (-0.634;0.362)
DG(36:3)	-0.543	0.587	0.894	-0.059 (-0.271;0.154)
Lysophosphatidylcholine(O-18:1)	-0.547	0.584	0.894	-0.128 (-0.588;0.331)
Lysophosphatidylethanolamine(20:4)	-0.576	0.564	0.894	-0.131 (-0.575;0.314)
Phosphatidylcholine(38:6)	0.552	0.581	0.894	0.141 (-0.36;0.642)
Sphingomyelin(d18:1/15:0)	-0.603	0.546	0.894	-0.144 (-0.611;0.323)
TG(44:2)	-0.591	0.555	0.894	-0.104 (-0.451;0.242)
TG(53:1)	-0.571	0.568	0.894	-0.137 (-0.609;0.334)
TG(60:1)	0.56	0.576	0.894	0.12 (-0.3;0.539)
Lysophosphatidylethanolamine(22:6)	0.511	0.609	0.899	0.123 (-0.349;0.595)
L-Methionine	0.506	0.613	0.9	0.117 (-0.335;0.568)
14,15-DiHETrE	0.492	0.622	0.91	0.119 (-0.356;0.594)
FA 22(5) w6	0.486	0.627	0.911	0.109 (-0.331;0.549)
Lysophosphatidylinositol 18(0)	-0.483	0.629	0.911	-0.113 (-0.574;0.347)
12,13-EpOME	-0.474	0.635	0.911	-0.129 (-0.664;0.405)
Lysophosphatidylcholine(14:0)	-0.477	0.633	0.911	-0.124 (-0.635;0.386)

Hydroxybutyric acid	-0.422	0.673	0.935	-0.088 (-0.499;0.322)
Taurocholic acid	-0.441	0.659	0.935	-0.115 (-0.627;0.396)
11-HETE	-0.417	0.676	0.935	-0.09 (-0.514;0.334)
8,9-DiHETrE	-0.428	0.669	0.935	-0.098 (-0.548;0.351)
Glycocholic acid	-0.432	0.666	0.935	-0.11 (-0.607;0.388)
Glycolithocholic acid	-0.426	0.67	0.935	-0.103 (-0.579;0.372)
Sphingomyelin(d18:1/14:0)	-0.436	0.663	0.935	-0.106 (-0.584;0.371)
TG(58:8)	0.42	0.674	0.935	0.102 (-0.375;0.58)
Lysophosphatidylethanolamine(18:0)	-0.409	0.682	0.938	-0.102 (-0.591;0.387)
Sphingomyelin(d18:1/24:2)	0.403	0.687	0.94	0.091 (-0.352;0.534)
L-Isoleucine	-0.393	0.695	0.947	-0.086 (-0.517;0.344)
Cystathionine	0.336	0.737	0.96	0.063 (-0.307;0.434)
Decenoylcarnitine	0.342	0.732	0.96	0.084 (-0.399;0.568)
Lysophosphatidylethanolamine (20:5)	0.334	0.739	0.96	0.083 (-0.402;0.567)
Lysophosphatidylcholine(18:0)	-0.339	0.735	0.96	-0.082 (-0.554;0.391)
Lysophosphatidylcholine(18:1)	0.347	0.728	0.96	0.086 (-0.399;0.571)
Lysophosphatidylcholine(O-16:0)	-0.37	0.711	0.96	-0.101 (-0.634;0.433)
Phosphatidylcholine(36:1)	-0.361	0.718	0.96	-0.075 (-0.484;0.334)
Phosphatidylcholine(36:2)	-0.336	0.737	0.96	-0.069 (-0.468;0.331)
Phosphatidylcholine(O-38:5)	-0.357	0.721	0.96	-0.096 (-0.622;0.43)
TG(58:2)	0.354	0.723	0.96	0.06 (-0.272;0.392)
TG(60:3)	0.333	0.739	0.96	0.088 (-0.431;0.608)
Homocysteine	0.294	0.769	0.973	0.066 (-0.375;0.508)
L-Valine	0.291	0.771	0.973	0.065 (-0.374;0.504)
Valerylcarnitine	-0.295	0.768	0.973	-0.072 (-0.551;0.407)
3-Hydroxypropionic Acid	-0.281	0.779	0.973	-0.062 (-0.493;0.369)
Lysophosphatidylinositol 18(2)	-0.287	0.774	0.973	-0.068 (-0.536;0.399)
5-HETE	-0.273	0.785	0.973	-0.063 (-0.514;0.389)
Cer(d18:0/23:0)	-0.274	0.784	0.973	-0.068 (-0.556;0.42)
Phosphatidylcholine(34:3)	-0.27	0.787	0.973	-0.072 (-0.595;0.451)
Phosphatidylcholine(40:8)	-0.285	0.775	0.973	-0.062 (-0.49;0.365)
Sphingomyelin(d18:1/20:0)	0.294	0.769	0.973	0.071 (-0.4;0.542)
Sphingomyelin(d18:1/21:0)	-0.29	0.772	0.973	-0.074 (-0.574;0.426)
Sphingomyelin(d18:1/22:0)	-0.274	0.784	0.973	-0.068 (-0.552;0.416)
Phosphatidylcholine(O-38:4)	-0.264	0.792	0.975	-0.062 (-0.525;0.401)
Symmetric dimethylarginine	-0.251	0.802	0.975	-0.064 (-0.566;0.438)
L-Tryptophan	-0.254	0.8	0.975	-0.057 (-0.495;0.381)
TG(54:0)	0.252	0.801	0.975	0.069 (-0.465;0.602)
Sphingomyelin(d18:1/24:0)	-0.245	0.807	0.977	-0.061 (-0.548;0.426)
Sphingomyelin(d18:1/23:1)	-0.24	0.811	0.978	-0.056 (-0.518;0.405)
L-Alpha-aminobutyric acid	-0.235	0.814	0.978	-0.059 (-0.551;0.433)
L-4-hydroxy-proline	-0.228	0.819	0.981	-0.055 (-0.526;0.416)
Methyldopa	-0.212	0.832	0.992	-0.018 (-0.187;0.15)

1-Methylhistidine	0.027	0.978	0.992	0.006 (-0.457;0.469)
3-Methoxytyrosine	0.113	0.91	0.992	0.004 (-0.071;0.079)
L-Alanine	-0.178	0.859	0.992	-0.043 (-0.514;0.428)
L-Asparagine	0.068	0.946	0.992	0.015 (-0.422;0.452)
Gamma-L-glutamyl-L-alanine	-0.023	0.982	0.992	-0.005 (-0.419;0.409)
L-Glutamine	-0.022	0.982	0.992	-0.005 (-0.454;0.444)
Glycylglycine	-0.099	0.921	0.992	-0.024 (-0.491;0.444)
L-Histidine	-0.032	0.975	0.992	-0.008 (-0.478;0.462)
Homocitrulline	-0.04	0.968	0.992	-0.01 (-0.504;0.484)
L-Leucine	-0.085	0.933	0.992	-0.019 (-0.448;0.411)
Methionine sulfone	-0.145	0.885	0.992	-0.033 (-0.485;0.419)
N6,N6,N6-Trimethyl-L-lysine	-0.035	0.972	0.992	-0.007 (-0.396;0.382)
Butyrylcarnitine	0.104	0.917	0.992	0.028 (-0.506;0.563)
Choline	-0.155	0.877	0.992	-0.038 (-0.523;0.446)
Deoxycarnitine	-0.104	0.917	0.992	-0.016 (-0.321;0.288)
Isovalerylcarnitine	-0.075	0.94	0.992	-0.018 (-0.487;0.451)
Octenoylcarnitine	0.049	0.961	0.992	0.011 (-0.446;0.469)
FA 18(2)	-0.077	0.939	0.992	-0.018 (-0.48;0.443)
Lysophosphatidylinositol 20(4)	0.075	0.94	0.992	0.018 (-0.453;0.488)
Sphingosine 1-phosphate 18(1)	-0.147	0.883	0.992	-0.014 (-0.203;0.175)
Taurochenodeoxycholic acid	-0.044	0.965	0.992	-0.011 (-0.525;0.502)
Taurolithocholic acid	-0.146	0.884	0.992	-0.036 (-0.515;0.444)
Deoxycholic acid	0.054	0.957	0.992	0.012 (-0.432;0.456)
DGLEA	0.161	0.872	0.992	0.034 (-0.379;0.447)
CE(18:1)	0.032	0.975	0.992	0.007 (-0.433;0.448)
Cer(d18:1/23:0)	0.121	0.904	0.992	0.033 (-0.503;0.569)
Cer(d18:1/24:1)	0.051	0.959	0.992	0.016 (-0.59;0.621)
Lysophosphatidylcholine(16:1)	0.163	0.87	0.992	0.04 (-0.444;0.525)
Lysophosphatidylcholine(18:2)	0.171	0.865	0.992	0.041 (-0.431;0.513)
Lysophosphatidylcholine(18:3)	-0.165	0.869	0.992	-0.056 (-0.721;0.609)
Phosphatidylcholine(32:0)	-0.194	0.846	0.992	-0.043 (-0.476;0.39)
Phosphatidylcholine(32:1)	0.133	0.894	0.992	0.031 (-0.428;0.49)
Phosphatidylcholine(32:2)	-0.157	0.875	0.992	-0.038 (-0.512;0.436)
Phosphatidylcholine(36:5)	0.146	0.884	0.992	0.038 (-0.467;0.542)
Phosphatidylcholine(38:2)	-0.137	0.891	0.992	-0.026 (-0.396;0.344)
Phosphatidylcholine(38:5)	-0.192	0.848	0.992	-0.047 (-0.528;0.433)
Phosphatidylcholine(O-34:2)	-0.045	0.964	0.992	-0.013 (-0.578;0.552)
Phosphatidylcholine(O-34:3)	-0.089	0.929	0.992	-0.021 (-0.493;0.45)
Phosphatidylethanolamine(O-38:7)	-0.154	0.878	0.992	-0.044 (-0.612;0.523)
Sphingomyelin(d18:1/18:0)	0.073	0.942	0.992	0.016 (-0.416;0.449)
Sphingomyelin(d18:1/18:1)	0.181	0.856	0.992	0.035 (-0.345;0.415)
Sphingomyelin(d18:1/22:1)	-0.164	0.87	0.992	-0.037 (-0.479;0.405)
Sphingomyelin(d18:1/24:1)	0.129	0.897	0.992	0.03 (-0.425;0.485)

Sphingomyelin(d18:1/25:0)	0.131	0.896	0.992	0.031 (-0.427;0.488)
TG(55:1)	0.106	0.916	0.992	0.029 (-0.503;0.56)
TG(56:2)	-0.06	0.952	0.992	-0.013 (-0.427;0.402)
Phosphatidylethanolamine(38:2)	-0.017	0.986	0.993	-0.004 (-0.487;0.478)
(+/-) 16-HDoHE	0.008	0.993	0.997	0.001 (-0.296;0.298)
Sphingosine 1-phosphate 18(2)	0.002	0.999	0.999	0 (-0.281;0.281)

Shown are the results of the fixed effect meta-analysis: regression coefficient (CI: 95% confidence interval), nominal and adjusted p-values.

Table S10. Associations between metabolite levels in idiopathic Parkinson's disease patients not treated with L-Dopa (L-Dopa^{negative}, n=30) and disease duration

**Name**	**z-value**	**Nominal *p***	**Adjusted *p***	**Beta (CI)**
Hexadecenoylcarnitine	2.529	0.011	0.145	0.068 (0.015;0.12)
Phosphatidylcholine(36.4)	2.169	0.03	0.277	0.075 (0.007;0.143)
Phosphatidylcholine(38.7)	2.027	0.043	0.324	0.067 (0.002;0.131)
TG(56.6)	1.926	0.054	0.357	0.053 (-0.001;0.106)
Tetradecenoylcarnitine	1.688	0.091	0.415	0.058 (-0.009;0.125)
FA18(1)	Jan 64	0.101	0.44	0.059 (-0.011;0.129)
Oleoylcarnitine	1.542	0.123	0.445	0.045 (-0.012;0.102)
TG(50.1)	1.288	0.198	0.573	0.029 (-0.015;0.073)
Glutathione	1.249	0.211	0.582	0.032 (-0.018;0.082)
Phosphatidylcholine(O.36.5)	1.211	0.226	0.613	0.035 (-0.021;0.091)
TG(52.1)	1.021	0.307	0.683	0.025 (-0.023;0.072)
TG(54.7)	0.755	0.45	0.821	0.029 (-0.046;0.104)
Glycoursodeoxycholic acid	0.726	0.468	0.838	0.018 (-0.03;0.065)
1-AG/2-AG	-0.715	0.475	0.839	-0.026 (-0.098;0.046)
1-LG/2-LG	-0.67	0.503	0.86	-0.021 (-0.083;0.041)
Lysophosphatidic acid 16(0)	0.578	0.563	0.86	0.017 (-0.041;0.075)
Lysophosphatidic acid 14(0)	-0.479	0.632	0.877	-0.016 (-0.084;0.051)
Phosphatidylethanolamine(36:4)	0.463	0.643	0.877	0.012 (-0.037;0.06)
Citric acid	0.399	0.69	0.882	0.013 (-0.05;0.075)
Putrescine	0.168	0.867	0.951	0.004 (-0.044;0.053)
Tiglylcarnitine	0.096	0.924	0.965	0.004 (-0.081;0.089)
Phosphatidylethanolamine(34:2)	-0.044	0.965	0.991	-0.001 (-0.062;0.059)
Thromboxane B2	0.019	0.985	0.995	0.001 (-0.054;0.055)

Shown are the results of association analyses: regression coefficient (CI: 95% confidence interval), nominal and adjusted p-values.

Table S11. Associations between metabolite levels in idiopathic Parkinson's disease patients not treated with L-Dopa (L-Dopa^{negative}, n=30) and Hoehn and Yahr scores

Name	z-value	Nominal *p*	Adjusted *p*	Beta (CI)
Glutathione	-2.192	0.028	0.583	-0.475 (-0.9;-0.05)
Putrescine	-2.17	0.03	0.583	-0.49 (-0.932;-0.047)
1-AG/2-AG	1.423	0.155	0.936	0.391 (-0.148;0.93)
Tiglylcarnitine	1.366	0.172	0.936	0.397 (-0.173;0.966)
Phosphatidylethanolamine(36:4)	-1.189	0.235	0.936	-0.34 (-0.899;0.22)
Thromboxane B2	1.121	0.262	0.936	0.255 (-0.191;0.702)
1-LG/2-LG	1.043	0.297	0.936	0.223 (-0.196;0.641)
FA18(1)	-0.981	0.327	0.936	-0.308 (-0.925;0.308)
TG(56.6)	0.818	0.414	0.936	0.228 (-0.319;0.775)
Phosphatidylcholine(O.36.5)	0.732	0.464	0.936	0.184 (-0.309;0.677)
Glycoursodeoxycholic acid	0.665	0.506	0.936	0.156 (-0.303;0.615)
Tetradecenoylcarnitine	-0.659	0.51	0.936	-0.198 (-0.786;0.39)
Hexadecenoylcarnitine	-0.43	0.667	0.936	-0.115 (-0.638;0.409)
Lysophosphatidic acid 16(0)	-0.407	0.684	0.936	-0.108 (-0.63;0.413)
TG(54.7)	0.403	0.687	0.936	0.135 (-0.522;0.792)
TG(52.1)	-0.387	0.699	0.936	-0.083 (-0.505;0.339)
Phosphatidylethanolamine(34:2)	-0.335	0.737	0.947	-0.094 (-0.643;0.455)
Phosphatidylcholine(38.7)	0.334	0.738	0.947	0.109 (-0.532;0.75)
Lysophosphatidic acid 14(0)	-0.157	0.875	0.969	-0.046 (-0.615;0.524)
Phosphatidylethanolamine(36:4)	0.138	0.89	0.969	0.044 (-0.586;0.674)
Oleoylcarnitine	-0.11	0.912	0.969	-0.028 (-0.531;0.474)
TG(50.1)	0.065	0.948	0.98	0.013 (-0.382;0.408)
Citric acid	-0.036	0.971	0.986	-0.011 (-0.611;0.589)

Shown are the results of association analyses: regression coefficient (CI: 95% confidence interval), nominal and adjusted p-values.

Table S12. Metabolites differently expressed in mitoPD patients with L-Dopa treatment (n=16) compared to healthy controls (n=64)

Name	z-value	Nominal p	Adjusted p	Beta (CI)
3-Methoxytyrosine	1.879	4.62e-144	1.41e-141	1.95 (1.873;2.026)
Methyldopa	1.348	2.28e-26	3.47e-24	1.571 (1.339;1.804)
(+/-) 16-HDoHE	0.546	0.00177	0.108	0.809 (0.474;1.144)
5-HETE	0.913	0.00123	0.108	0.547 (0.309;0.784)
8-HETE	0.691	0.00156	0.108	0.582 (0.297;0.868)
11-HETE	0.79	0.0057	0.26	0.493 (0.171;0.814)
15(S)-HETrE	0.684	0.006	0.26	0.486 (0.158;0.813)
L-Tyrosine	0.743	0.007	0.266	0.403 (0.099;0.708)
L-4-hydroxy-proline	0.741	0.0103	0.312	0.416 (0.099;0.734)
Putrescine	0.619	0.00959	0.312	-0.424 (-0.742;-0.105)
Sphingosine 1-phosphate 18(1)	0.327	0.0182	0.426	-0.446 (-0.803;-0.089)
Prostaglandin F2α	0.634	0.0179	0.426	-0.39 (-0.708;-0.073)
TG(60:2)	0.593	0.0164	0.426	-0.367 (-0.67;-0.064)
Ornithine	0.721	0.0285	0.54	0.324 (0.052;0.597)
3-Hydroxypropionic Acid	0.659	0.0302	0.54	0.377 (0.052;0.703)
8,12-iPF2a IV	0.543	0.0253	0.54	-0.321 (-0.611;-0.031)
8(9)-EpETrE	0.331	0.03	0.54	-0.345 (-0.66;-0.03)
Lysophosphatidic acid 16(1)	0.557	0.0351	0.575	-0.266 (-0.513;-0.019)
Lysophosphatidylinositol 20(4)	0.573	0.036	0.575	0.354 (0.016;0.692)
Gamma-aminobutyric acid	0.501	0.0621	0.589	-0.361 (-0.711;-0.012)
L-Glutamic acid	0.554	0.0433	0.589	-0.295 (-0.587;-0.004)
Glycylglycine	0.562	0.0533	0.589	-0.345 (-0.68;-0.009)
Sarcosine	0.469	0.0771	0.589	-0.375 (-0.742;-0.007)
Trimethylamine N-oxide	0.369	0.0835	0.589	-0.336 (-0.673;0)
Sphingosine 1-phosphate 18(0)	0.244	0.0827	0.589	-0.352 (-0.708;0.003)
Sphingosine 1-phosphate 18(2)	0.38	0.0522	0.589	-0.232 (-0.568;0.103)
12-HETE	0.258	0.0547	0.589	0.204 (-0.095;0.503)
14,15-DiHETrE	0.565	0.0497	0.589	-0.216 (-0.506;0.074)
Anandamide	0.699	0.0485	0.589	0.322 (-0.015;0.659)
DGLEA	0.541	0.0654	0.589	0.222 (-0.096;0.54)
DG(36:2)	0.213	0.0764	0.589	0.249 (-0.108;0.606)
Lysophosphatidylcholine(16:0)	-0.543	0.0563	0.589	-0.265 (-0.619;0.09)
Lysophosphatidylcholine(18:0)	-0.528	0.0872	0.589	-0.2 (-0.417;0.017)
Lysophosphatidylcholine(18:2)	-0.616	0.0504	0.589	-0.221 (-0.524;0.082)
Lysophosphatidylethanolamine(18:0)	-0.584	0.0668	0.589	-0.25 (-0.59;0.09)
Lysophosphatidylethanolamine(18:1)	-0.195	0.0577	0.589	-0.229 (-0.523;0.066)
Phosphatidylcholine(36:1)	-0.432	0.0716	0.589	-0.268 (-0.605;0.069)
Phosphatidylcholine(38:5)	-0.434	0.0617	0.589	-0.256 (-0.58;0.068)
Phosphatidylcholine(40:8)	-0.458	0.0831	0.589	0.249 (-0.067;0.566)
Phosphatidylethanolamine(38:2)	-0.462	0.0693	0.589	0.25 (-0.095;0.595)

Sphingomyelin(d18:1/16:0)	-0.516	0.0837	0.589	0.148 (-0.063;0.358)
Sphingomyelin(d18:1/16:1)	-0.483	0.078	0.589	-0.21 (-0.522;0.102)
TG(56:2)	0.544	0.0832	0.589	-0.287 (-0.623;0.049)
TG(58:2)	0.458	0.0662	0.589	0.214 (-0.095;0.523)
TG(59:1)	0.372	0.0856	0.589	0.199 (-0.07;0.467)
Lysophosphatidylcholine(O-16:1)	-0.517	0.0898	0.593	0.291 (-0.057;0.639)
Lysophosphatidylcholine(20:5)	-0.424	0.093	0.602	0.292 (-0.039;0.623)
DG(36:3)	0.224	0.0973	0.616	-0.325 (-0.698;0.047)
TG(56:4)	0.528	0.103	0.638	-0.229 (-0.572;0.113)
Sphingomyelin(d18:1/22:1)	-0.454	0.108	0.656	-0.253 (-0.61;0.103)
Hydroxylysine	0.52	0.12	0.658	-0.256 (-0.622;0.109)
Serotonine	-0.3	0.122	0.658	-0.242 (-0.601;0.118)
Lysophosphatidic acid 14(0)	0.383	0.122	0.658	-0.29 (-0.628;0.048)
Lysophosphatidylethanolamine (20:4)	0.473	0.125	0.658	-0.218 (-0.534;0.098)
O-Anandamide	0.519	0.128	0.658	-0.299 (-0.666;0.069)
Phosphatidylcholine(36:5)	-0.432	0.127	0.658	-0.303 (-0.643;0.037)
Phosphatidylcholine(38:6)	-0.44	0.114	0.658	-0.255 (-0.575;0.064)
Phosphatidylcholine(O-34:3)	-0.508	0.123	0.658	-0.267 (-0.585;0.051)
TG(54:0)	-0.472	0.126	0.658	-0.329 (-0.713;0.056)
L-2-aminoadipic acid	0.332	0.172	0.682	-0.342 (-0.717;0.034)
Cystathionine	0.317	0.16	0.682	-0.369 (-0.765;0.026)
O-Acetyl-L-serine	0.394	0.175	0.682	-0.338 (-0.694;0.018)
L-Phenylalanine	0.482	0.156	0.682	-0.3 (-0.674;0.074)
Saccharopine	0.422	0.152	0.682	-0.328 (-0.704;0.048)
Citric acid	0.38	0.181	0.682	-0.248 (-0.595;0.1)
FA 18(1)	0.447	0.152	0.682	-0.279 (-0.632;0.074)
Lysophosphatidylinositol 18(1)	-0.374	0.171	0.682	0.272 (-0.063;0.607)
Palmitoyl ethanolamide	0.445	0.163	0.682	-0.321 (-0.729;0.088)
CE(18:2)	-0.438	0.161	0.682	0.253 (-0.106;0.612)
CE(20:4)	-0.398	0.143	0.682	-0.26 (-0.616;0.095)
CE(22:6)	-0.434	0.154	0.682	-0.222 (-0.541;0.098)
Lysophosphatidylcholine(18:1)	-0.405	0.182	0.682	-0.27 (-0.612;0.072)
Phosphatidylcholine(32:0)	-0.379	0.175	0.682	-0.268 (-0.61;0.073)
Phosphatidylcholine(40:6)	-0.363	0.181	0.682	-0.331 (-0.672;0.011)
Phosphatidylcholine(40:7)	-0.39	0.136	0.682	-0.24 (-0.586;0.105)
Phosphatidylethanolamine(38:4)	-0.407	0.155	0.682	-0.277 (-0.634;0.08)
Phosphatidylethanolamine(38:6)	-0.396	0.162	0.682	-0.228 (-0.569;0.113)
Phosphatidylethanolamine(O-38:7)	-0.446	0.151	0.682	-0.296 (-0.645;0.053)
Sphingomyelin(d18:1/15:0)	-0.419	0.178	0.682	-0.272 (-0.629;0.085)
TG(56:1)	0.283	0.17	0.682	-0.334 (-0.698;0.03)
TG(56:3)	0.435	0.159	0.682	-0.26 (-0.603;0.084)
9,10,13-TriHOME	0.314	0.187	0.693	-0.25 (-0.623;0.122)
9-HODE	0.384	0.189	0.693	-0.302 (-0.678;0.074)

Ethanolamine	0.353	0.2	0.722	-0.315 (-0.684;0.055)
FA 22(5) w6	0.371	0.215	0.76	-0.301 (-0.627;0.025)
TG(54:7)	-0.369	0.215	0.76	-0.295 (-0.66;0.07)
N6,N6,N6-Trimethyl-L-lysine	0.303	0.258	0.824	-0.282 (-0.65;0.085)
Choline	0.404	0.25	0.824	-0.331 (-0.698;0.035)
Tiglylcarnitine	-0.331	0.255	0.824	-0.335 (-0.727;0.056)
Malic acid	0.294	0.24	0.824	-0.256 (-0.628;0.116)
Lysophosphatidylethanolamine(22:6)	-0.31	0.258	0.824	-0.278 (-0.647;0.09)
Sphingomyelin(d18:1/23:0)	-0.359	0.255	0.824	-0.184 (-0.463;0.094)
Sphingomyelin(d18:1/23:1)	-0.342	0.256	0.824	-0.192 (-0.482;0.099)
Sphingomyelin(d18:1/24:2)	-0.314	0.253	0.824	-0.21 (-0.532;0.112)
TG(44:2)	0.31	0.242	0.824	-0.215 (-0.549;0.119)
Glycodeoxycholic acid	-0.302	0.265	0.838	-0.22 (-0.562;0.122)
O-Phosphoethanolamine	0.133	0.294	0.843	-0.212 (-0.55;0.126)
Isobutyrylcarnitine	-0.311	0.308	0.843	0.212 (-0.126;0.55)
Oleoylcarnitine	0.29	0.29	0.843	-0.168 (-0.439;0.103)
Isocitrate	0.253	0.276	0.843	-0.205 (-0.546;0.135)
Lysophosphatidic acid 16(0)	0.292	0.292	0.843	0.111 (-0.072;0.294)
11,12-DiHETrE	0.319	0.3	0.843	-0.214 (-0.568;0.14)
13-HODE	0.28	0.294	0.843	-0.193 (-0.512;0.127)
Glycoursodeoxycholic acid	0.366	0.275	0.843	-0.211 (-0.558;0.137)
CE(18:1)	-0.285	0.277	0.843	-0.199 (-0.531;0.133)
Lysophosphatidylcholine(14:0)	-0.304	0.307	0.843	-0.183 (-0.499;0.133)
Lysophosphatidylcholine(22:6)	-0.324	0.306	0.843	0.199 (-0.142;0.539)
Phosphatidylcholine(34:2)	-0.294	0.287	0.843	-0.169 (-0.464;0.126)
Sphingomyelin(d18:1/14:0)	-0.305	0.301	0.843	0.213 (-0.15;0.575)
TG(53:1)	0.376	0.286	0.843	-0.192 (-0.528;0.144)
TG(55:1)	0.315	0.293	0.843	-0.195 (-0.526;0.136)
Lysophosphatidylcholine(O-16:0)	-0.347	0.311	0.844	0.179 (-0.134;0.493)
Hexadecenoylcarnitine	0.265	0.315	0.846	-0.198 (-0.535;0.138)
(+/-) 8-HDoHE	0.289	0.324	0.864	-0.183 (-0.499;0.133)
L-Glutamine	-0.306	0.331	0.875	-0.202 (-0.547;0.144)
FA 22(6)	-0.285	0.335	0.879	-0.218 (-0.606;0.17)
Sphingomyelin(d18:1/22:0)	-0.311	0.339	0.881	-0.209 (-0.588;0.169)
TG(52:1)	-0.261	0.343	0.884	-0.195 (-0.552;0.161)
L-Alpha-aminobutyric acid	-0.286	0.381	0.895	-0.193 (-0.546;0.16)
Gamma-Glutamylglutamine	0.243	0.407	0.895	-0.193 (-0.554;0.168)
L-Serine	0.245	0.459	0.895	-0.177 (-0.507;0.153)
Taurine	0.113	0.473	0.895	-0.182 (-0.523;0.16)
Betaine	-0.225	0.463	0.895	-0.184 (-0.535;0.167)
Dodecenoylcarnitine	0.235	0.412	0.895	-0.173 (-0.504;0.158)
Hydroxybutyric acid	0.182	0.471	0.895	-0.196 (-0.571;0.179)
3-Hydroxybutyric acid	0.242	0.432	0.895	0.179 (-0.168;0.525)

Lactic acid	0.228	0.424	0.895	-0.158 (-0.464;0.148)
Lysophosphatidic acid 18(1)	0.227	0.387	0.895	0.163 (-0.158;0.485)
Lysophosphatidylethanolamine (16:0)	-0.265	0.471	0.895	0.166 (-0.168;0.499)
Lysophosphatidylethanolamine (16:1)	0.242	0.394	0.895	-0.152 (-0.467;0.163)
LPS 18(1)	0.138	0.472	0.895	-0.163 (-0.495;0.168)
Taurochenodeoxycholic acid	-0.293	0.364	0.895	0.175 (-0.181;0.531)
Taurolithocholic acid	-0.227	0.477	0.895	-0.17 (-0.516;0.177)
(+/-) 10-HDoHE	0.135	0.459	0.895	-0.158 (-0.483;0.168)
(+/-) 11-HDoHE	0.145	0.419	0.895	-0.177 (-0.544;0.189)
12,13-DiHODE	-0.301	0.42	0.895	-0.185 (-0.556;0.186)
12(S)-HEPE	0.097	0.473	0.895	0.163 (-0.177;0.504)
17,18-DiHETE	-0.228	0.413	0.895	0.156 (-0.171;0.482)
9,12,13-TriHOME	0.234	0.445	0.895	-0.162 (-0.503;0.179)
CHOLIC ACID	0.22	0.405	0.895	0.182 (-0.208;0.572)
Lysophosphatidylcholine(18:3)	-0.268	0.437	0.895	-0.17 (-0.538;0.197)
Lysophosphatidylcholine(20:4)	-0.259	0.4	0.895	0.158 (-0.186;0.503)
Phosphatidylcholine(32:1)	0.249	0.431	0.895	-0.146 (-0.47;0.177)
Phosphatidylcholine(36:2)	-0.16	0.443	0.895	-0.156 (-0.506;0.193)
Phosphatidylcholine(36:4)	-0.246	0.353	0.895	-0.162 (-0.532;0.207)
Phosphatidylcholine(38:2)	-0.185	0.383	0.895	-0.154 (-0.504;0.197)
Phosphatidylcholine(38:7)	-0.211	0.372	0.895	-0.153 (-0.508;0.201)
Phosphatidylcholine(O-36:3)	-0.164	0.452	0.895	0.129 (-0.175;0.432)
Phosphatidylethanolamine(36:4)	-0.211	0.457	0.895	0.14 (-0.187;0.467)
Phosphatidylethanolamine(O-36:5)	-0.239	0.477	0.895	-0.146 (-0.488;0.197)
Sphingomyelin(d18:1/18:1)	-0.19	0.448	0.895	-0.139 (-0.465;0.187)
Sphingomyelin(d18:1/18:2)	-0.156	0.452	0.895	-0.157 (-0.531;0.216)
Sphingomyelin(d18:1/21:0)	-0.266	0.406	0.895	-0.15 (-0.507;0.207)
Sphingomyelin(d18:1/24:1)	-0.221	0.452	0.895	0.135 (-0.196;0.465)
Sphingomyelin(d18:1/25:0)	-0.224	0.394	0.895	0.142 (-0.205;0.489)
TG(42:0)	0.154	0.366	0.895	-0.125 (-0.433;0.183)
TG(45:0)	0.141	0.357	0.895	-0.142 (-0.503;0.219)
TG(46:2)	0.236	0.411	0.895	0.117 (-0.216;0.45)
TG(48:3)	0.246	0.466	0.895	0.121 (-0.221;0.463)
TG(50:1)	-0.236	0.372	0.895	0.125 (-0.214;0.465)
TG(54:3)	0.244	0.423	0.895	0.126 (-0.209;0.461)
TG(56:0)	0.14	0.44	0.895	0.125 (-0.226;0.477)
L-Isoleucine	0.18	0.501	0.918	-0.133 (-0.488;0.222)
L-Threonine	0.209	0.507	0.918	-0.125 (-0.453;0.203)
L-Tryptophan	-0.197	0.499	0.918	-0.108 (-0.404;0.187)
Taurocholic acid	-0.228	0.497	0.918	-0.122 (-0.46;0.216)
14,15-DiHETE	-0.187	0.506	0.918	-0.133 (-0.476;0.211)
TG(56:5)	0.185	0.5	0.918	-0.137 (-0.494;0.22)
Dehydroepiandrosterone	-0.208	0.52	0.932	0.129 (-0.236;0.494)

Sphingomyelin(d18:1/24:0)	-0.202	0.521	0.932	-0.135 (-0.515;0.245)
Glycine	-0.169	0.583	0.936	-0.127 (-0.477;0.224)
Homocitrulline	0.176	0.573	0.936	-0.132 (-0.488;0.224)
L-Lysine	0.19	0.562	0.936	-0.123 (-0.454;0.207)
S-Methylcysteine	-0.192	0.542	0.936	-0.13 (-0.482;0.222)
L-Valine	-0.147	0.58	0.936	0.118 (-0.222;0.458)
Decenoylcarnitine	0.149	0.597	0.936	-0.111 (-0.463;0.24)
Tetradecenoylcarnitine	0.162	0.537	0.936	-0.113 (-0.457;0.232)
Lysophosphatidylethanolamine (20:5)	-0.165	0.594	0.936	0.107 (-0.217;0.431)
12,13-DiHOME	0.176	0.601	0.936	0.116 (-0.233;0.464)
8,9-DiHETrE	0.144	0.581	0.936	-0.047 (-0.196;0.102)
Cortisol	0.219	0.546	0.936	-0.113 (-0.467;0.241)
Glycolithocholic acid	-0.174	0.561	0.936	0.103 (-0.223;0.43)
Cer(d18:1/24:1)	0.185	0.574	0.936	-0.104 (-0.411;0.203)
Lysophosphatidylcholine(O-18:1)	-0.171	0.549	0.936	0.107 (-0.234;0.448)
Phosphatidylcholine(32:2)	-0.156	0.597	0.936	-0.115 (-0.48;0.25)
Phosphatidylcholine(38:4)	-0.141	0.551	0.936	-0.101 (-0.407;0.204)
Phosphatidylcholine(O-34:2)	-0.185	0.592	0.936	-0.113 (-0.474;0.248)
Phosphatidylcholine(O-36:4)	-0.175	0.599	0.936	-0.112 (-0.441;0.217)
Phosphatidylcholine(O-36:5)	-0.183	0.547	0.936	-0.117 (-0.481;0.247)
Phosphatidylethanolamine(34:2)	-0.148	0.559	0.936	-0.094 (-0.398;0.211)
Sphingomyelin(d18:1/20:0)	-0.157	0.596	0.936	-0.102 (-0.442;0.238)
TG(42:2)	0.173	0.551	0.936	-0.099 (-0.449;0.251)
TG(46:1)	0.18	0.553	0.936	-0.085 (-0.388;0.219)
TG(51:2)	-0.152	0.599	0.936	-0.089 (-0.407;0.229)
TG(56:7)	-0.181	0.529	0.936	0.092 (-0.216;0.4)
Nonaylcarnitine	-0.161	0.608	0.939	0.095 (-0.238;0.428)
9-HOTrE	0.156	0.608	0.939	-0.082 (-0.364;0.2)
TG(54:4)	0.163	0.612	0.939	-0.092 (-0.403;0.22)
Butyrylcarnitine	0.143	0.629	0.953	0.089 (-0.231;0.41)
TG(42:1)	0.113	0.629	0.953	-0.114 (-0.489;0.261)
TG(46:0)	-0.126	0.63	0.953	-0.108 (-0.472;0.256)
Phosphatidylcholine(34:3)	-0.135	0.638	0.955	0.106 (-0.268;0.48)
TG(58:8)	-0.117	0.636	0.955	-0.109 (-0.484;0.265)
Pimelylcarnitine	0.131	0.641	0.956	0.101 (-0.266;0.468)
Octanoylcarnitine	0.123	0.65	0.959	-0.056 (-0.261;0.149)
Glycocholic acid	-0.141	0.656	0.959	-0.081 (-0.39;0.228)
TG(50:2)	-0.123	0.648	0.959	-0.082 (-0.392;0.228)
TG(56:6)	-0.116	0.656	0.959	0.089 (-0.254;0.432)
9,10-DiHOME	0.128	0.666	0.969	0.084 (-0.249;0.417)
DL-3-aminoisobutyric acid	0.136	0.671	0.971	-0.089 (-0.437;0.26)
Glutathione	0.1	0.691	0.971	0.089 (-0.261;0.438)
L-Histidine	0.105	0.733	0.971	-0.089 (-0.434;0.256)

L-Leucine	0.096	0.722	0.971	-0.089 (-0.432;0.254)
Methionine sulfone	-0.124	0.692	0.971	-0.086 (-0.43;0.258)
2-Methylbutyroylcarnitine	-0.104	0.73	0.971	0.084 (-0.26;0.428)
Linoleylcarnitine	-0.095	0.739	0.971	-0.051 (-0.259;0.156)
Malonylcarnitine	0.114	0.686	0.971	-0.084 (-0.431;0.263)
Propionylcarnitine	0.108	0.722	0.971	0.022 (-0.069;0.114)
Tetradecadienylcarnitine	0.09	0.738	0.971	-0.069 (-0.377;0.24)
Valerylcarnitine	0.11	0.716	0.971	0.075 (-0.263;0.412)
Pyroglutamic acid	0.097	0.734	0.971	-0.037 (-0.205;0.13)
FA 18(2)	-0.109	0.74	0.971	-0.074 (-0.392;0.243)
Lysophosphatidic acid 18(2)	0.092	0.72	0.971	-0.081 (-0.443;0.28)
Taurodeoxycholic acid	-0.114	0.741	0.971	0.078 (-0.259;0.414)
Glycochenodeoxycholic acid	-0.112	0.705	0.971	-0.067 (-0.358;0.224)
Linoleoyl ethanolamide	0.12	0.711	0.971	-0.073 (-0.401;0.255)
Thromboxane-B2	-0.086	0.733	0.971	-0.088 (-0.479;0.304)
Phosphatidylcholine(O-34:1)	-0.095	0.738	0.971	0.071 (-0.267;0.409)
Phosphatidylcholine(O-38:6)	-0.117	0.717	0.971	-0.027 (-0.161;0.107)
Phosphatidylcholine(O-44:5)	-0.13	0.699	0.971	-0.075 (-0.445;0.295)
Phosphatidylethanolamine(O-38:5)	-0.134	0.703	0.971	0.071 (-0.28;0.422)
TG(48:1)	-0.107	0.722	0.971	-0.027 (-0.163;0.109)
Phosphatidylcholine(34:1)	-0.025	0.746	0.974	-0.069 (-0.419;0.281)
Citrulline	-0.075	0.753	0.975	-0.062 (-0.39;0.267)
TG(54:2)	0.092	0.754	0.975	0.061 (-0.262;0.384)
Homocysteine	-0.088	0.759	0.975	0.07 (-0.297;0.436)
Stearoylcarnitine	0.082	0.761	0.975	-0.06 (-0.397;0.277)
1-Methylhistidine	-0.044	0.883	0.982	-0.059 (-0.387;0.269)
L-Alanine	-0.042	0.889	0.982	0.049 (-0.232;0.329)
L-Arginine	0.057	0.841	0.982	0.05 (-0.249;0.349)
Cysteine	-0.069	0.831	0.982	-0.052 (-0.365;0.26)
Gamma-L-glutamyl-L-alanine	0.054	0.84	0.982	0.051 (-0.308;0.41)
L-Kynurenine	-0.074	0.81	0.982	0.054 (-0.291;0.399)
L-Methionine	0.049	0.857	0.982	0.044 (-0.254;0.343)
L-Proline	0.066	0.853	0.982	0.042 (-0.246;0.329)
Symmetric dimethylarginine	-0.04	0.904	0.982	-0.028 (-0.224;0.168)
Carnitine	0.091	0.807	0.982	-0.056 (-0.413;0.301)
Decanoylcarnitine	0.047	0.859	0.982	-0.047 (-0.364;0.27)
Deoxycarnitine	-0.033	0.866	0.982	-0.054 (-0.392;0.284)
Hexanoylcarnitine	0.057	0.836	0.982	-0.056 (-0.42;0.309)
Lauroylcarnitine	-0.036	0.89	0.982	-0.056 (-0.428;0.316)
Methylmalonylcarnitine	-0.044	0.883	0.982	-0.055 (-0.44;0.33)
Palmitoylcarnitine	-0.073	0.771	0.982	-0.044 (-0.361;0.272)
Lysophosphatidylinositol 16(1)	0.052	0.867	0.982	-0.039 (-0.337;0.259)
Lysophosphatidylinositol 18(2)	0.073	0.791	0.982	-0.038 (-0.376;0.3)

1-LG/2-LG	-0.043	0.877	0.982	0.037 (-0.281;0.356)
(+/-) 14-HDoHE	0.036	0.779	0.982	-0.016 (-0.16;0.127)
Prostaglandin E2	0.045	0.857	0.982	0.017 (-0.133;0.167)
Cer(d18:0/22:0)	-0.037	0.899	0.982	0.043 (-0.285;0.371)
Cer(d18:1/22:0)	0.048	0.85	0.982	0.041 (-0.318;0.399)
Cer(d18:1/23:0)	0.095	0.774	0.982	-0.045 (-0.407;0.317)
Lysophosphatidylcholine(16:1)	0.074	0.793	0.982	-0.046 (-0.412;0.32)
Lysophosphatidylethanolamine(20:4)	-0.064	0.818	0.982	-0.04 (-0.386;0.306)
Phosphatidylcholine(34:4)	-0.049	0.874	0.982	0.044 (-0.327;0.416)
Phosphatidylcholine(36:3)	-0.08	0.784	0.982	-0.046 (-0.404;0.312)
Phosphatidylcholine(38:3)	0.067	0.811	0.982	0.035 (-0.3;0.371)
Phosphatidylcholine(O-38:4)	0.04	0.895	0.982	0.036 (-0.308;0.38)
Phosphatidylcholine(O-38:5)	-0.073	0.825	0.982	-0.034 (-0.357;0.289)
Sphingomyelin(d18:1/18:0)	0.06	0.812	0.982	0.034 (-0.301;0.369)
TG(44:0)	-0.029	0.896	0.982	-0.033 (-0.368;0.302)
TG(44:1)	0.06	0.825	0.982	-0.037 (-0.414;0.339)
TG(48:2)	0.034	0.911	0.982	-0.033 (-0.375;0.31)
TG(51:1)	0.051	0.871	0.982	-0.022 (-0.267;0.223)
TG(51:4)	0.089	0.776	0.982	-0.033 (-0.397;0.331)
TG(52:2)	-0.029	0.91	0.982	-0.026 (-0.331;0.279)
TG(52:3)	0.073	0.796	0.982	-0.028 (-0.361;0.306)
TG(54:1)	-0.031	0.909	0.982	0.023 (-0.295;0.341)
TG(54:5)	0.038	0.904	0.982	-0.023 (-0.338;0.291)
TG(57:1)	-0.056	0.806	0.982	0.012 (-0.139;0.162)
TG(58:9)	-0.042	0.865	0.982	-0.027 (-0.39;0.336)
TG(60:1)	-0.078	0.796	0.982	0.026 (-0.341;0.392)
TG(60:3)	0.065	0.837	0.982	0.019 (-0.273;0.312)
Acetylcarnitine	0.023	0.939	0.989	0.02 (-0.311;0.352)
Isovalerylcarnitine	-0.026	0.929	0.989	-0.018 (-0.348;0.312)
Myristoilcarnitine	-0.018	0.939	0.989	0.019 (-0.344;0.381)
19,20-DiHDPA	0.022	0.942	0.989	-0.02 (-0.385;0.344)
Lysophosphatidylcholine(20:3)	0.02	0.939	0.989	0.019 (-0.352;0.39)
Phosphatidylcholine(40:5)	0.021	0.938	0.989	-0.018 (-0.395;0.358)
Sphingomyelin(d18:1/25:1)	-0.022	0.943	0.989	-0.013 (-0.325;0.298)
TG(50:4)	0.031	0.923	0.989	-0.014 (-0.351;0.323)
L-Asparagine	0.018	0.954	0.989	0.015 (-0.345;0.375)
Lysophosphatidylinositol 18(0)	-0.017	0.953	0.989	-0.005 (-0.346;0.337)
12,13-EpOME	0.022	0.947	0.989	0.01 (-0.313;0.332)
Sphingomyelin(d18:1/20:1)	0.013	0.957	0.989	0.004 (-0.342;0.35)
TG(52:4)	-0.014	0.962	0.991	0.009 (-0.335;0.354)
L-Homoserine	-0.004	0.987	0.994	-0.007 (-0.315;0.301)
Octenoylcarnitine	0.011	0.97	0.994	-0.005 (-0.289;0.28)
2-Ketoglutaric acid	0.008	0.977	0.994	0.005 (-0.323;0.332)

Deoxycholic acid	0.005	0.984	0.994	-0.004 (-0.321;0.313)
Cer(d18:0/23:0)	0.006	0.986	0.994	-0.003 (-0.344;0.337)
TG(50:3)	0.01	0.973	0.994	0.003 (-0.34;0.347)
TG(51:3)	-0.007	0.982	0.994	-0.007 (-0.388;0.374)
1-AG/2-AG	0.002	0.994	0.997	-0.007 (-0.366;0.351)
5,6-DiHETrE	0	0.999	0.999	0.007 (-0.358;0.372)

Shown are the results of the fixed effect meta-analysis: regression coefficient (CI: 95% confidence interval), nominal and adjusted p-values.

Table S13. Details on the method, participants and covariates included in each analysis

Analysis	Method	Dependent variable and covariates	Individuals and metabolites
Site differences	Linear regression for each metabolite separately<br><br>Adjustment of P-values using Benjamini-Hochberg approach	Dependent variable: Transformed metabolite intensity<br>Independent variables: age at examination, sex, Diagnosis.group	204 controls and IPD patients<br><br>304 metabolites
Association with disease status	For each metabolite and pairwise comparison:<br><br>Analysis separately for each site:<br>- linear regression<br><br>Fixed effect meta-analysis and adjustment of P-values using Benjamini-Hochberg approach	Dependent variable: Transformed metabolite intensity, ratios or sums<br><br>Independent variables: Diagnosis.group, Sex, AAE	64 controls<br>140 IPD patients<br><br>304 metabolites
Comparison control vs IPD patients with and without Levodopa	For each metabolite and pairwise comparison:<br><br>Analysis separately for each site:<br>- linear regression<br><br>Fixed effect meta-analysis and adjustment of P-values using Benjamini-Hochberg approach	Dependent variable: Transformed metabolite intensity, ratios or sums<br><br>Independent variables: treatment_group, sex, age at examination	30 No levodopa<br>110 Levodopa<br>64 HC<br><br>304 metabolites
Association with age (In cases and controls)	For each metabolite analysis separately for each site:<br>Linear regression<br><br>Fixed effect meta-analysis of interaction term and adjustment of P-values using Benjamini-Hochberg approach	Dependent variable: Transformed metabolite intensity, ratios or sums<br><br>Independent variables: Age at examination, Sex, Diagnosis.group	204 controls and IPD patients<br><br>304 metabolites
Association with UPDRS III total score (IPD patients without Levodopa)	For each metabolite and pairwise comparison:<br><br>Analysis separately for each site:<br>Linear regression<br><br>Fixed effect meta-analysis and adjustment of P-values using Benjamini-Hochberg approach	Dependent variable: Transformed metabolite intensity, ratios or sums<br><br>Independent variables: MDS-UPDRS III Total Score, Sex	29 IPD patients without Levodopa<br><br>304 metabolites

Analysis	Method	Dependent variable and covariates	Individuals and metabolites
Association with disease duration (IPD patients without Levodopa)	For each metabolite and pairwise comparison:<br><br>Analysis separately for each site: Linear regression<br><br>Fixed effect meta-analysis and adjustment of P-values using Benjamini-Hochberg approach	Dependent variable: Transformed metabolite intensity, ratios or sums<br><br>Independent variables: Disease Duration, Sex, age at examination	29 IPD patients without Levodopa<br><br>304 metabolites
Association with Hoehn and Yahr staging (IPD patients without Levodopa)	For each metabolite and pairwise comparison:<br><br>Analysis separately for each site: Linear regression<br><br>Fixed effect meta-analysis and adjustment of P-values using Benjamini-Hochberg approach	Dependent variable: Transformed metabolite intensity, ratios or sums<br><br>Independent variables: HoehnandYahrStaging, Sex.	29 IPD patients without Levodopa<br><br>304 metabolites

Figure S1. Forrest Plots to display differences in both study sites regarding comparing L-Dopa^{positive} IPD patients to healthy controls for five metabolites.

A

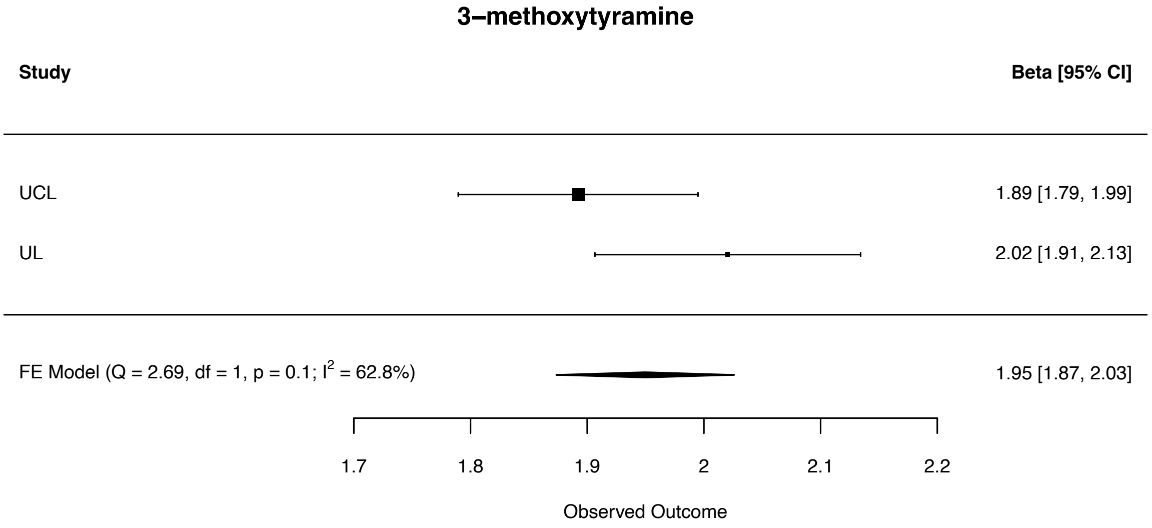

B

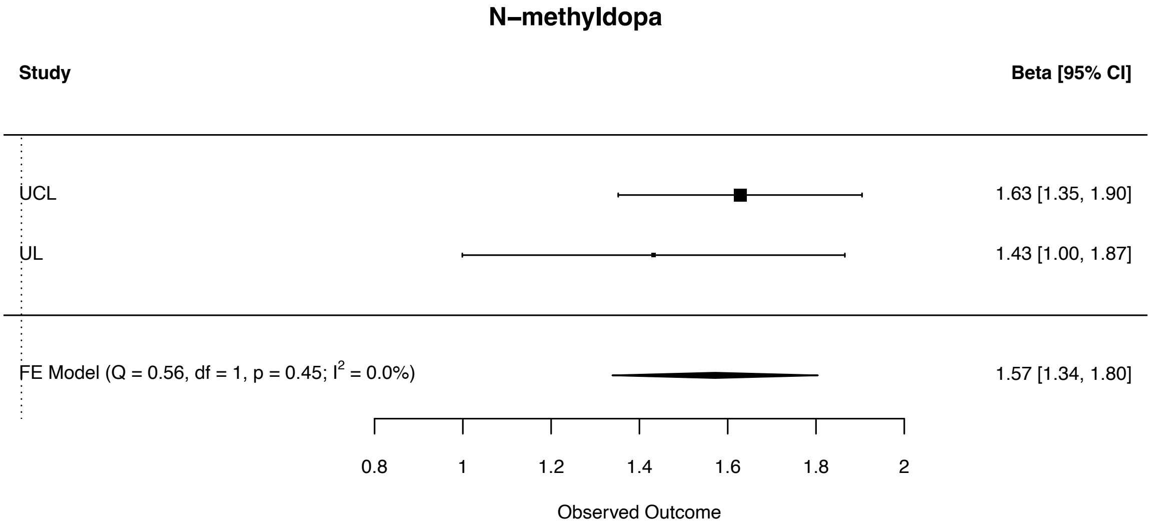

C

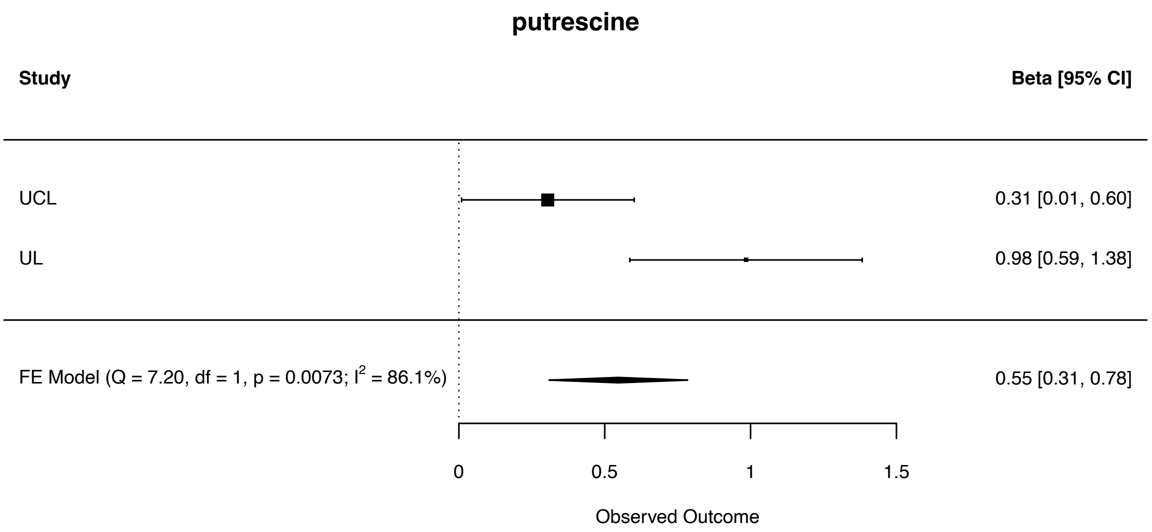
putrescine
Study
Beta [95% CI]
UCL
0.31 [0.01, 0.60]
UL
0.98 [0.59, 1.38]
FE Model (Q = 7.20, df = 1, p = 0.0073; I2 = 86.1%)
0.55 [0.31, 0.78]
0
0.5
1
1.5
Observed Outcome

D

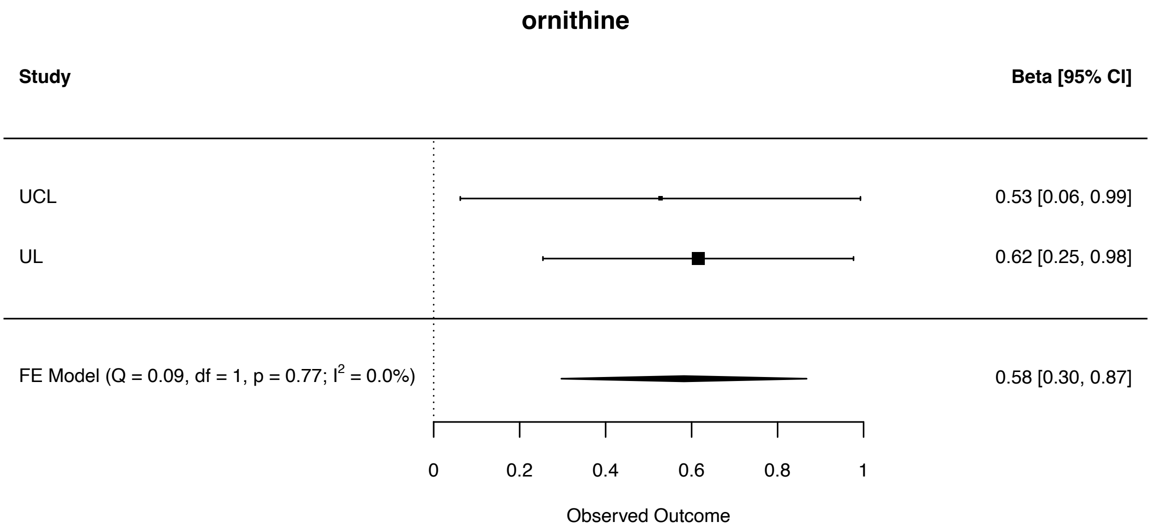
ornithine
Study
Beta [95% CI]
UCL
0.53 [0.06, 0.99]
UL
0.62 [0.25, 0.98]
FE Model (Q = 0.09, df = 1, p = 0.77; I2 = 0.0%)
0.58 [0.30, 0.87]
0
0.2
0.4
0.6
0.8
1
Observed Outcome

E

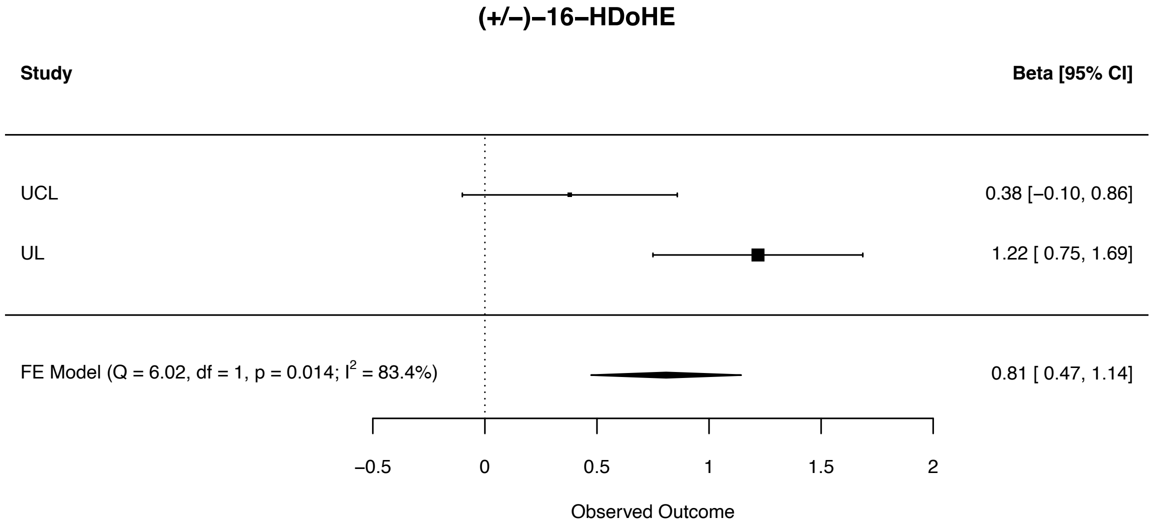

UL= University of Lübeck; UCL= University College London; CI= Confidence Interval; FE = fixed-effects model; Q, df and p = test statistic, degrees of freedom and P-value of Cochran's Q-test of heterogeneity; I^2 = heterogeneity statistic
